# Improved Access to Chiral Tetranaphthoazepinium-Based Organocatalysts Using Aqueous Ammonia as Nitrogen Source

**DOI:** 10.3390/molecules24213844

**Published:** 2019-10-25

**Authors:** Auraya Manaprasertsak, Sorachat Tharamak, Christina Schedl, Alexander Roller, Michael Widhalm

**Affiliations:** 1 Department of Chemistry, Faculty of Science, Kasetsart University, Bangkok 10900, Thailand; auraya.ma@ku.th (A.M.); sorachat.th@ku.th (S.T.); 2 Institute of Organic Chemistry, University of Vienna, Währinger Straße 38, 1090 Wien, Austria; christina.schedl@univie.ac.at; 3 Institute of Inorganic Chemistry, University of Vienna, Währinger Straße 42, Wien 1090, Austria; alexander.roller@univie.ac.at; 4 Institute of Chemical Catalysis, University of Vienna, Währinger Straße 38, 1090 Wien, Austria

**Keywords:** asymmetric phase transfer catalysis, organo catalysis, 1,1′-binaphthyls, optical resolution, chiral catalyst synthesis

## Abstract

The class of 3,3′-diaryl substituted tetranaphthobisazepinium bromides has found wide application as highly efficient *C*_2_-symmetrical phase-transfer catalysts (PTCs, Maruoka type catalysts). Unfortunately, the synthesis requires a large number of steps and hampers the build-up of catalyst libraries which are often desired for screening experiments. Here, we present a more economic strategy using dinaphthoazepine **7** as the common key intermediate. Only at this stage various aryl substituents are introduced, and only two individual steps are required to access target structures. This protocol was applied to synthesize ten tetranaphthobisazepinium compounds **1a**–**1j**. Their efficiency as PTCs was tested in the asymmetric substitution of *tert*-butyl 2-((diphenylmethylene)amino)acetate. Enantioselectivities up to 92% have been observed with new catalysts.

## 1. Introduction

In many asymmetric transformations the atropisomeric 1,1’-binaphthyl moiety serves as a highly efficient chiral backbone [1]. The possibility to introduce suitable functional groups, particularly at C-2 and C-2’, as well as at C-3 and C-3’ has made the binaphthyl group an indispensable chiral modifyer in stoichiometric and catalytic asymmetric synthesis [2,3,4,5,6,7,8]. While functionalities at C-2 and C-2′ serve particularly as sites for primary interaction in organocatalysis or coordination in transition metal catalysis, substituents at C-3 and C-3′ are introduced for secondary substrate or reagent activation or to tune steric interaction.

**Figure 1:**
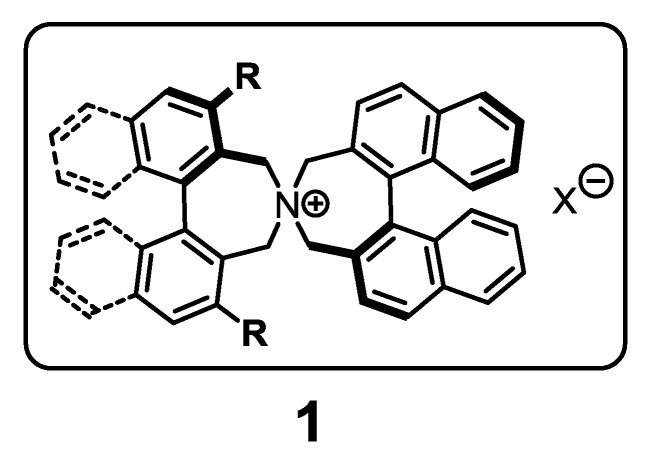
*N-spiro*-di- and tetranaphthoazepinium salts (R = aryl).

One particularly successful subgroup are *N-spiro*-di- and tetranaphthoazepinium salts **1** (Figure 1) which found application as efficient phase-transfer catalysts (PTCs) [9,10,11,12,13,14,15,16,17,18,19,20,21,22,23,24]. Several strategies for their synthetic access have been developed, particularly by Maruoka’s group. All are based on non-racemic 2,2’-dihydroxy-1,1’-binaphthol or 1,1’-binaphthyl-2,2’-dicarboxylic acid or corresponding biphenyl precursors and comprise *ortho*-metallation steps, introduction of aryl groups at C-3 and C-3’, and manipulation of functional groups at C-2/C-2’ to end up with 2,2′-bromomethyl groups suitable for cyclization with allylamine. Cleavage of the *N*-allyl substituent is required before dibenzylation with a second 2,2’-bis(bromo)methyl-1,1’-binaphthyl moiety forms the ammonium salts of general structure **1**. When starting from non-racemic binaphthol, typically 13 to 15 steps are required to obtain the ammonium salt [25] or in an alternative approach six steps from diacid **2** to obtain the 3,3’ substituted subunit [26]. Although the yields are satisfying, the expenditure of time is still high. 

**Scheme 1:**
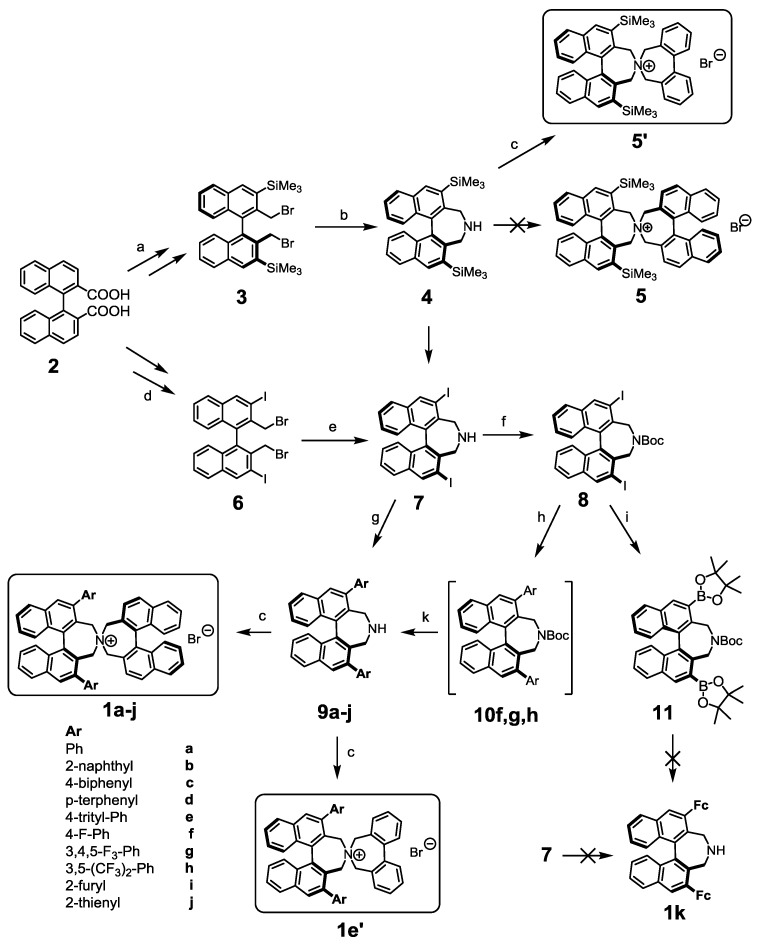
Synthesis of *spiro*-azepinium catalysts **1a**–**j**. *Legend*: a. [39]; b. NH_3_, MeCN, 65–70 °C, overnight (85%); c. (*S*)-2,2′-bisbromomethyl-1,1′-binaphthyl or 2,2′-bisbromomethyl-1,1′-biphenyl (**1e’**, **5′**), K_2_CO_3_, MeCN, 85–90 °C, overnight (50%–88%); d. [39]; e. [29]; f. Boc_2_O, Amberlyst-15, EtOH, 10 min (84%); g. ArB(OH)_2_, Na_2_CO_3_, Pd(Ph_3_P)_4_ or (dppf)PdCl_2_ (**9d**), toluene, 12–24 h, (55%–98%); h. ArB(OH)_2_, Na_2_CO_3_, Pd(OAc)_2_, tol_3_P, toluene, 12–24 h; i. B_2_Pin_2_, KOAc, Pd(OAc)_2_, DMF, 100 °C, 20 h (66%); k. trifluoroacetic acid (TFA), dichloromethane (DCM), r.t., 2 h (60%–80% over 2 steps).

With the aim to facilitate the built-up of ligand libraries [27,28] we recently developed a strategy introducing 3,3′-aryl substituents at a late stage of the synthesis, and replacing allylamine with ammonia. Among others spiro-ammonium salts **1a**–**1c** containing electron donating aryl groups were synthesized in good yield (Scheme 1) [29].

In the present paper we further extend this protocol to the introduction of electron withdrawing or more bulky aryl substituents to give amines **9d**–**9j**. Subsequent cyclisation with 2,2’-bis(bromo)methyl-1,1’-binaphthyl afforded seven tetranaphthobisazepinium bromides **1d**–**1j** which were tested in prototypical organocatalyzed reactions. Moreover, an optical resolution procedure of key intermediate **7** is provided.

## 2. Results and Discussion

With the intention to introduce various aryl groups at C-3/3′ of the binaphthyl core but at a late stage of the synthesis, diiodoazepine **7** was chosen as key intermediate. Its synthesis started from 1,1’-binaphthyl-2,2’-dicarboxylic acid **2** which is accessible in enantiomerically pure form on the multigram scale from non-racemic 2,2′-dimethyl-1,1′-binaphthyl [30] or 2,2’-binaphthol in two to four steps [31,32,33], respectively or as a racemate applying various procedures from the literature [34]. As we noticed that **1** (R = H, X = Br) can be conveniently prepared from non-racemic 2,2’-bis(bromomethyl)-1,1’-binaphthyl and aqueous ammonia in 94% yield [35], it was easy to apply similar conditions for 3,3’-substituted substrates with the expectation that steric hindrance might stop the reaction at the stage of the secondary amine. (For use of ammonia in the synthesis of symmetrical *N*-spiroazepine compounds, see [36,37,38].) As precursors **3** and **6** were prepared from **2** in three or four steps (overall yield: 47% for **3**, 48% for **6**) according to literature [39]. Treatment of dibromides **3** and **6** with aqueous ammonia (25%) in acetonitrile at 60 °C overnight afforded **4** and **7** in 85% and 88% [29], respectively. An alternative approach to **7** via the bis(trimethylsilyl)azepine **4** gave lower yield. An attempted *spiro*-cyclisation of **4** to **5** failed; only minor amounts of rearranged products could be identified (see Supplementary Materials). Non-racemic **4** and **7** can be prepared from enantiomerically pure precursors (*R*)- and (*S*)-**2** but requires optical resolution of the diacid. Applying published procedures, this step is quite time consuming and uses toxic or expensive amines as resolving agents, which are often difficult to recycle. Consequently alternatives were considered (see also comments in Supporting Materials of [29]). Instead we found it more economic to work out an optical resolution procedure for the secondary amine **7** preferably using some cheap chiral acid as resolving agent. This seemed most appropriate as it is the common key intermediate for all catalysts and at the end of the synthetic path.

**Figure 2:**
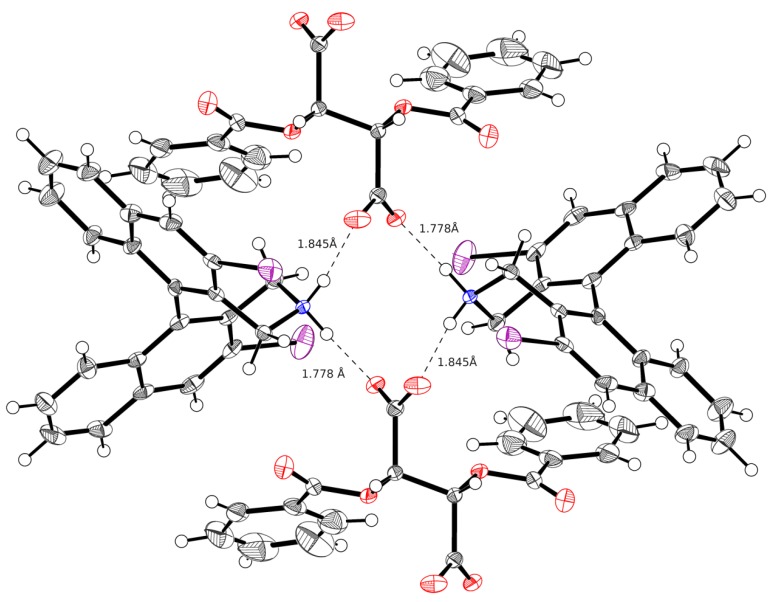
X-ray structure of [(*R*)-**7**]_2_ (*S,S*)-O,O’-Dibenzoyltartrate. Cation [7]^+^ and counter ion form two independent hydrogen bonds constructing a rhombus. Bond length and rhombus visualized.

*Optical resolution of***7**: A procedure similar to that published for the enantioseparation of the unsubstituted analogue 3,5-dihydro-4*H*-dinaphth[2,1-*c*:1’,2’-*e*]azepine was applied [40]. A 2:1 mixture of *rac*-**7** and (*S,S*)-di-*O*-benzoyltartaric acid in dichloromethane (DCM)/MeOH-deposited colourless needles upon standing for several hours at r.t. An X-ray structure analyses of a single crystal showed a di-(*R*)-azepium tartrate with local *C_2_*-symmetry and one molecule of DCM in the asymmetric unit (Figure 2). After removal of the auxiliary and one recrystallisation (*R*)- and (*S*)-**7** were isolated in approximately 40% yield. The enantiomeric purity was determined by chiral high-performance liquid chromatography (HPLC) to be ≥99% (see Experimental section) [41].

For the synthesis of azepinium compounds **1** arylation of **7** followed by reaction with non-racemic 2,2′-bisbromomethyl-1,1′-binaphthyl worked well [42]. A practicable route to target structures **1** might be therefore **2** → **6** → **7** (optical resolution) → **9** → **1**. The general feasability was previously testet in the synthesis of **1a**–**1c** [29] and was now extended to *spiro* ammonium compounds **1d**–**1j** from enantiomerically pure **7**. Suzuki-Miyaura reactions with appropriate boronic acids or tetramethyldioxaborolanes afforded **9d**, **9e**, **9i**, and **9j** in fair to good yield. Only for electron-withdrawing aryl groups the yields for Suzuki coupling were low (49% and 29% for **9f** and **9h**, respectively). In those cases *N*-Boc protected diiodide **8** was a proper intermediate (60%–80% for **9f**–**9h**). In contrast, coupling of **7** or **8** with ferroceneboronic acid or bisborolane **11** with 1-bromoferrocene failed to give **9k**. The final step, the formation of *spiro*-ammonium compounds **1**, proceeded under standard conditions with satisfying yields except for **1e** which gave a bad mixture. The target compound was isolated by only 7% after repeated chromatography. We speculate that, similarly to **4**, steric strain in **1e** might facilitate subsequent Stevens rearrangement under slightly basic conditions. Two side products with correct high-resolution mass spectrometry (HRMS) and ^1^H-nuclear magnetic resonance (NMR) multiplets (two AB and one ABX systems corresponding to Ar-CH_2_-N and Ar-CH_2_-CH(N)-Ar) have been detected in the product mixture (See Supplementary Materials). To lower steric repulsion within the target molecule we cyclized azepines **4** and **9e** with 2,2′-bisbromomethyl-1,1′-biphenyl yielding **5′** and **1e’** in good yield. 

**Scheme 2:**
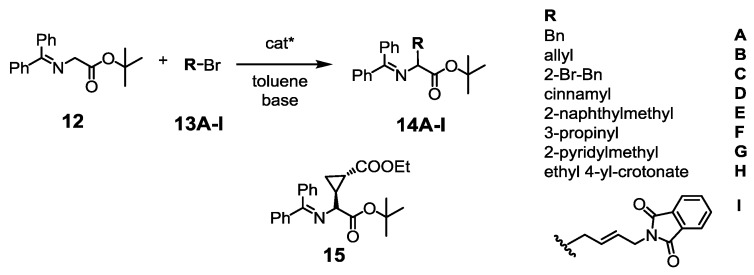
Asymmetric substitution of **12** with **13A**–**I** under phase-transfer catalysis (PTC) conditions.

*Organocatalysis:* With this catalyst library in hand we next wanted to create a reactivity/selectivity profile for application in phase-transfer reactions (PTC) under strictly standardised conditions using the well known α-benzylation of tert-butyl 2-((diphenylmethylene)amino)acetate **12** with benzylbromide **13A**, one of the most popular conversions to test a new PTC (Scheme 2). Further on, with promising catalysts we were also interested to introduce substituents with functional groups to extend the scope for application. 

In a preliminary study new ammonium salts (S,S)-**1a**–**1j**, (S)-**1e’**, and (S)-**5′** were tested in the asymmetric benzylation of *tert*-butyl 2-((diphenylmethylene)amino)acetate **12** under standard PTC conditions (Table 1) [43,44]. For all experiments we compared “expected yields” based on integration and “isolated yields” after chromatography to demonstrate equivalence of methods (Table 1, row 3 and 4). Typically a loss of 1%–5% of product after chromatography was observed. We attribute this to variing quality of column packing, incomplete separation, and changes in adsorbent activity. In addition, interaction of the substrate and product with silicagel resulting in partial cleavage of the imino group might be responsible for reduced isolated yields after purification [45]. Therefore, we considered reporting of the “chemical yield” by comparison of NMR signals of product and added internal standard more reliable and moreover, time-saving than an “isolated yield”. 

**Figure 3:**
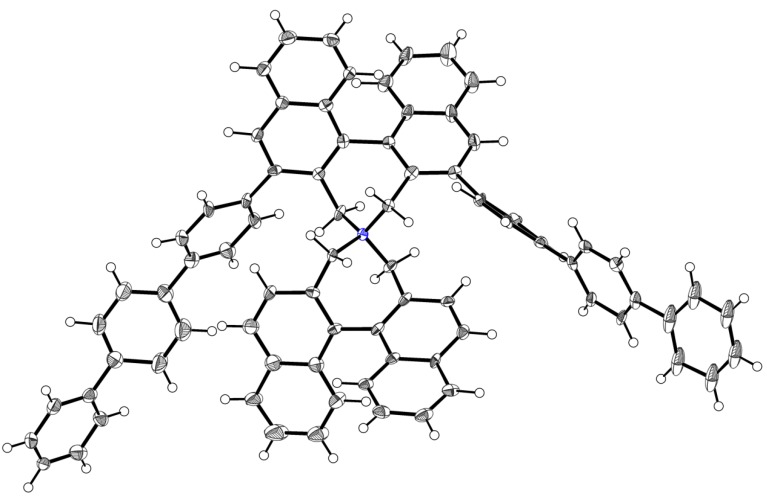
Crystal structure of (*R*)(*R*)-**1d**. Solvent and counter ion omitted for clarity.

**Figure 4:**
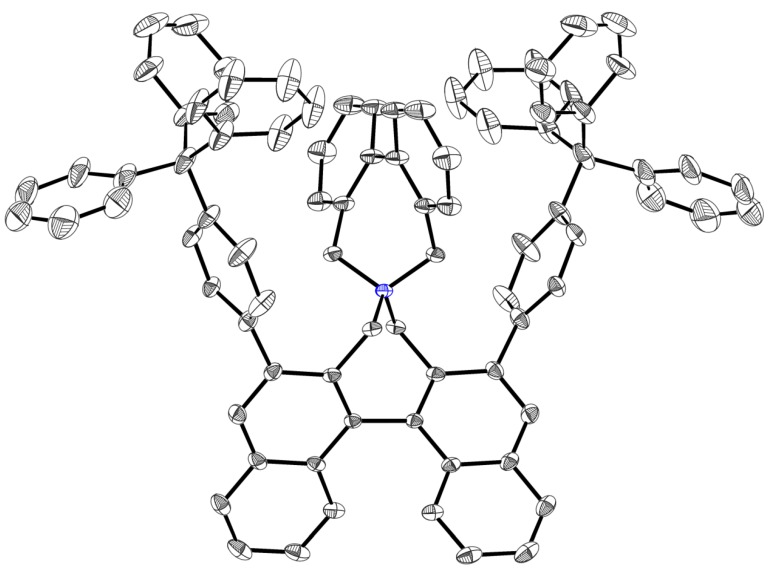
Crystal structure of (R)_bina_(S)_biphe_-**1e**’. Solvent, counter ion and hydrogens omitted for clarity.

In agreement with previous findings **1b**, **1f**, and **1g** were most efficient [25]. Literature results could be reproduced in most cases, although reported conditions were in part different from our’s (see notes in Table 1). Elongation of 3,3′-substituents (**1c**, **1d**, see also X-ray structure Figure 3) did not further increase selectivity or activity, in several cases even reduced the asymmetric induction (entry 3,4). Particularly remarkable was the failure of **1e’** yielding an almost racemic product in moderate yield (entry 5). This is also in line with the low ee obtained with **5′** (entry 11). We suspect that two effects might be responsible for destroying the asymmetric induction: (1) the strong predominance of the biphenyl atropomer with a configuration opposite to that of the binaphthyl as it was also found in the X-ray structure of **1e’** (Figure 4), overriding eventually even a higher reactivity of the minor species with “homo-chirality”; and (2) the presence of more spherical and not distinctly directed substituents which might disguise the *C_2_* symmetry of the catalyst. Also, the introduction of hetero aromates (**1i, 1j**) gave lower ee (entry 9,10).

After this preliminary estimation of reactivity and enantioselectivity of new *spiro*-ammonium catalysts we choose the more promising candidates **1c** and **1d** for next investigations and compared their efficiency with known catalysts with different reactivity **1g** [25] and **1h** [47]. Activated benzylic bromides (**13B**–**F**, Scheme 2) were tested under same conditions as in Table 1 to make results comparable (0 °C, 20 h, 1 mol % of catalyst in toluene with 50% KOH, 0.25 mmol scale). In addition, also electrophiles **13G**–**I** with functional groups, eventually of interest for subsequent transformations, were included. As before, in all cases chemical yields based on NMR integration with IS were slightly higher (1%–4%) than those calculated from weighted products, isolated after chromatography (Table 2). Products **14B**–**F** were formed in good to excellent yield and enantioselectivity particularly with **1g**. Merely, **1h** showed pronounced low reactivity and in two cases unusual poor ee which can be attributed to a significant degree of background reaction. For sterically less demanding electrophiles **13B**, **13C**, and **13F** new ligands **1c** and **1d** were also effective, very similar to each other and also to the known 3,3′-bis(2-naphthyl) substituted analogue. Using more bulky/less reactive bromides **13D**, **13E** yields and/or enantioselectivity were lower in some cases. Also a pyridyl substituent could be introduced (**14G**) with use of catalysts **1c**, **1d**, **1g,** and **1h**. In all cases the reaction proceded smoothly giving comparable yield (81%–91%) and up to 83% ee. A more challenging electrophile was **13H** which formed only 13% of **14H** with 62% ee using the most reactive catalyst **1g** under standard conditions. As a side product cyclopropane **15** was produced as a single stereoisomer from a Michael addition followed by cyclisation. To accelerate the reaction KOH was replaced with CsOH. With these conditions complete conversion was achieved yielding 61% of **14H** (79% ee) and only 8% of **15**. While nearly the same result was obtained with **1h**, the enantioselectivtity with **1c** and **1d** remained low. Also the use of 4-iodocrotonate did not improve the results (for details see Supplementary Materials, Table S2). Finally, we aimed to introduce a *N*-protected alkenylamino substituent to obtain **14I**. In pre-experiments we noticed that use of strongly alkaline media resulted in considerable loss of product through ring opening of the phthalimide. Therefore, solid Cs_2_CO_3_ was used instead (33% yield, 88% ee). But even with the more reactive iodide (**13I** with I replacing Br) the yield was still low (20%–33%) and asymmetric induction moderate (51%–83% ee).

Summarising, we presented an alternative route to non-racemic 3,3′-aryl substituted tetranaphtho-spiro-bisazepinium bromides as demonstrated in the synthesis of **1a**–**1j** in seven to eight steps via 3,3′-substituted dinaphthoazepines **9a**–**9j** [48] using 1,1′-binaphthyl-2,2′-dicarboxylic acid as starting material and 3,3′-diiodo-dinaphthoazepine **7** as the common key intermediate. This scalable synthesis required only two chromatographic purification steps and target structures have been isolated in 23%–25% overall yield. A preliminary study on their use in PTC was conducted using a simplified screening protocol. Moderate to good asymmetric induction of 41%–92% ee has been observed with new catalysts.

## 3. Materials and Methods 

### 3.1. General Information

Melting points: Kofler melting point apparatus (Reichert Thermovar, Reichert Technologies, Depew, NY, USA), uncorrected. NMR: Bruker AV III 400 spectrometer) at 400.27 MHz (^1^H) and 100.66 MHz (^13^C), and Bruker AV III 600 at 600.25 MHz (^1^H) and 150.95 MHz (^13^C), respectively (Bruker Biospin, Billerica, MA, USA); chemical shifts δ are reported in ppm rel. to solvent signals (7.26 and 77.00 ppm for CHCl_3_ and CDCl_3_, respectively). Coupling patterns are designated as s(inglet), d(oublet), t(riplet), q(uartet), m(ultiplet), ps(eudo), and br(oad). ^13^C{^1^H} NMR spectra are recorded in a *J*-modulated mode; signals are assigned as C, CH, CH_2_, and CH_3_. HRMS spectra were obtained on a maXis ESI-Qq-TOF mass spectrometer (Bruker Daltonics, Bremen, Germany) in the positive-ion mode by direct infusion. For HPLC determination of chiral products, an Agilent 1200 chromatograph (Agilent Technologies, Santa Clara, CA, USA) equipped with a diode array detector and autosampler was used. Optical rotations were measured with a Perkin Elmer polarimeter 243 (PerkinElmer, Inc. Waltham, MA, USA) using a 1 dm thermostated cell. Column chromatography (MPLC) was performed on a Isolera One system (Biotage, Uppsala, Sweden) with self-packed columns, SiO_2_, 40–63 µm.

Heptane, dichloromethane (DCM), and ethyl acetate (EtOAc) were distilled, absolute THF from sodium benzophenone ketyl, Et_2_O from LiAlH_4_; acetonitrile, DCM, and triethylamine from CaH_2_; *n*-BuLi was used as 1.6 M solution in *n*-hexane (Aldrich). All the other chemicals were analytical grade and used without further purification.

Reported procedures have been followed to obtain non-racemic 2,2′-bis(bromomethyl)-1,1′-binaphthyl [49], azepine **7** [29], racemic and non-racemic 1,1′-binaphthyl-2,2′-dicarboxylic acid (required for the synthesis of **7**) [31,32,33,50,51], 2-([1,1’:4’,1’’-terphenyl]-4-yl)-4,4,5,5-tetramethyl-1,3,2-dioxaborolane, and 4,4,5,5-tetramethyl-2-(4-tritylphenyl)-1,3,2-dioxaborolane [52]. Syntheses of **9a** [29,53], **9b** [29], **9c** [29], **1a** [25,29,46], **1b** [29,46], and **1c** [25,29] have been published. 

### 3.2. Synthesis

*Spiro cyclisation of* (*S*)-**9a**–**9k** *with (S)-2,2′-bis(bromomethyl)-1,1′-binaphthyl yielding* (*S,S*)-**1a**–**1k**, *respectively.* (*General procedure A):* A Schlenk tube, equipped with magnetic stirring bar and glass stopper, was charged with a solution of substrate (0.3 mmol) in MeCN (6 mL) and K_2_CO_3_ (83 mg, 2 eq) followed by (*S*)-2,2′-bis(bromomethyl)-1,1′-binaphthyl (132 mg, 0.3 mmol) and the mixture was degassed. The reaction was left stirring at 85–90 °C (bath) for 24 h. After cooling to r.t., DCM (30 mL) and H_2_O (30 mL) were added and the phases were separated. The aqueous phase was extracted with DCM (3 × 15 mL). The combined organic extracts were evaporated under reduced pressure and the crude material was purified by MPLC using a solvent gradient (MeOH(0→8%)/DCM).

Synthesis of **1a** (76%), **1b** (88%), and **1c** (81%) was published recently applying the same protocol [29]. 

*(11bS,11b’S)-2,6-Di([1,1’:4’,1’’-terphenyl]-4-yl)-3,3’,5,5’-tetrahydro-4,4’-spirobi[dinaphtho[2,1-c:1’,2’-e]azepin]-4-ium bromide* (**1d**): 83% yield; mp: 255–260 °C (dec.), [α]_D_^20^ = +139 (c: 0.56, DCM). ^1^H-NMR δ: 8.31 (s, 2H); 8.00 (d, *J* = 8.2 Hz, 2H); 7.80–7.83 (m, 7H); 7.73–7.79 (m, 4H); 7.61 (d, *J* = 8.2 Hz, 2H); 7.55 (m, 4H); 7.37–7.46 (m, 4H); 7.31 (br.m, 2H); 7.10–7.20 (m, 6H); 7.08 (d, *J* = 8.6 Hz, 2H); 6.83 (br.m, 2H); 6.47 (d, *J* = 8.6 Hz, 2H); 4.88 (br.d, *J* = 13.9 Hz, 2H); 4.32 (d, *J* = 13.8 Hz, 2H); 4.31 (d, *J* = 13.9 Hz, 2H); 3.43 (br.d, *J* = 14.0 Hz, 2H) ppm. *Note:* in addition a broad band was observed (6.6–8.7 ppm) corresponding to ~9H. ^13^C-NMR δ: 141.03 (C); 140.93 (C); 140.49 (C); 139.15 (C); 138.92 (C); 138.90 (C); 138.33 (C); 136.20 (C); 134.05 (C); 133.78 (C); 132.43 (CH); 131.06 (C); 131.05 (C); 128.96 (CH); 128.67 (CH); 128.64 (CH); 128.10 (CH); 127.90 (2 CH); 127.66 (CH); 127.59 (CH); 127.57 (CH); 127.38 (CH); 127.28 (CH); 127.14 (CH); 127.12 (CH); 126.67 (CH); 125.49 (C); 122.88 (C); 61.88 (CH_2_); 56.89 (CH_2_) ppm. HRMS (ESI) calculated for C_80_H_56_N [M − Br]^+^: 1030.4407, found 1030.4412.

*(11bS,11b’S)-2,6-Bis(4-tritylphenyl)-3,3’,5,5’-tetrahydro-4,4’-spirobi[dinaphtho[2,1-c:1’,2’-e]azepin]-4-ium bromide* (**1e**): 7% yield. ^1^H-NMR δ: 8.23 (s, 2H); 8.00 (d, *J* = 8.2 Hz, 2H); 7.69 (d, *J* = 8.2 Hz, 2H); 7.49–7.55 (m, 4H); 7.41 (d, *J* = 8.4 Hz, 2H); 7.23-7.39 (m, ~40H); 7.17 (br.m, 2H); 7.16 (d, *J* = 8.6 Hz, 2H); 7.04 (d, *J* = 8.6 Hz, 2H); 6.86 (d, *J* = 8.4 Hz, 2H); 4.97 (d, *J* = 13.7 Hz, 2H); 4.42 (d, *J* = 13.6 Hz, 4H); 3.73 (br.d, *J* = 13.6 Hz, 2H) ppm. HRMS (ESI) calculated for C_94_H_68_N [M - Br]^+^: 1210.5347, found 1210.5327.

*(S)-2’,6’-Bis(4-tritylphenyl)-3’,5,5’,7-tetrahydrospiro[dibenzo[c,e]azepine-6,4’-dinaphtho[2,1-c:1’,2’-e]azepin]-6-ium bromide* (**1e’**): 66% yield; mp: 260–270 °C (dec.), [α]_D_^20^ = -130 (c: 0.68, DCM). ^1^H-NMR δ: 8.12 (s, 2H); 8.04 (d, *J* = 8.3 Hz, 2H); 7.81 (d, *J* = 7.6 Hz, 2H); 7.63 (t, *J* = 7.5 Hz, 2H); 7.51–7.56 (m, 4H); 7.46 (t, *J* = 7.5 Hz, 2H); 7.15–7.45 (br.m, ~12H); 7.10–7.18 (m, ~16H); 7.07 (dm, *J* = 8.6 Hz, 4H); 6.95 (dm, *J* = 7.6 Hz, 10H); 4.85 (d, *J* = 13.3 Hz, 2H); 4.65 (d, *J* = 13.3 Hz, 2H); 4.48 (d, *J* = 12.8 Hz, 2H); 2.76 (d, *J* = 12.8 Hz, 2H) ppm. ^13^C-NMR δ: 146.55 (C); 146.23 (C); 140.52 (C); 139.01 (C); 138.47 (C); 136.52 (C); 134.30 (C); 131.85 (CH); 131.16 (CH); 131.10 (C); 131.08 (CH); 130.96 (CH); 130.86 (CH); 128.98 (CH); 128.84 (CH); 128.03 (CH); 127.77 (CH); 127.45 (CH); 127.17 (CH); 127.07 (C); 126.01 (CH); 124.35 (C); 64.52 (C); 62.62 (CH_2_); 59.23 (CH_2_) ppm. HRMS (ESI) calculated for C_86_H_64_N [M − Br]^+^: 1110.5033, found 1110.5033.

*(S,S)-2,6-Bis(4-fluorophenyl)-3,3’,5,5’-tetrahydro-4,4’-spirobi[dinaphtho[2,1-c:1’,2’-e]azepin]-4-ium bromide* (**1f**) [25]: 50% yield. ^1^H-NMR δ: 8.28 (s, 2H); 8.08 (d, *J* = 8.2 Hz, 2H); 7.87 (d, *J* = 8.2 Hz, 2H); 7.59 (ddd, *J* = 8.0, 6.7, 1.1 Hz, 2H); 7.49 (ddd, *J* = 8.1, 6.7, 1.2 Hz, 2H); 7.41 (d, *J* = 8.3 Hz, 2H); 7.28 (ddd, *J* = 8.2, 6.7, 1.2 Hz, 2H); 7.19 (ddd, *J* = 8.2, 6.7, 1.1 Hz, 2H); 7.10 (dd, *J* = 9.0, 0.9 Hz, 2H); 7.08 (dd, *J* = 8.6, 0.9 Hz, 2H); 6.33 (d, *J* = 8.5 Hz, 2H); 4.93 (d, *J* = 14.0 Hz, 2H); 4.42 (dd, *J* = 14.0, 1.4 Hz, 2H); 4.24 (d, *J* = 13.6 Hz, 2H); 3.72 (dd, *J* = 13.5, 1.1 Hz, 2H) ppm; *Note:* in addition a broad band was observed (7.3–8.5 ppm) corresponding to ~8H. ^13^C-NMR δ: 163.25 (d, *J*_CF_ = 249.5 Hz, C); 139.31 (C); 138.05 (C); 136.32 (C); 135.50 (d, *J*_CF_ = 3.5 Hz, C); 133.92 (C); 132.85 (br.d, *J*_CF_ = 6.6 Hz, CH); 132.56 (CH); 131.11 (d, *J*_CF_ = 9.4 Hz, C); 128.70 (CH); 128.49 (CH); 128.37 (CH); 128.28 (CH); 127.54 (CH); 127.52 (CH); 127.41 (CH); 126.84 (CH); 126.67 (CH); 124.91 (C); 122.48 (C); 117.04 (d, *J*_CF_ = 21.7 Hz, CH); 62.46 (CH_2_); 57.42 (CH_2_) ppm. HRMS (ESI) calculated for C_56_H_38_F_2_N [M − Br]^+^: 762.2967, found 762.2951.

*(11bS,11b’S)-2,6-Bis(3,4,5-trifluorophenyl)-3,3’,5,5’-tetrahydro-4,4’-spirobi[dinaphtho[2,1-c:1’,2’-e]azepin]-4-ium bromide* (**1g**) [25]: 66% yield. ^1^H-NMR δ: 8.26 (s, 2H); 8.09 (d, *J* = 8.2 Hz, 2H); 7.93 (d, *J* = 8.2 Hz, 2H); 7.63 (ddd, *J* = 8.0, 6.8, 1.1 Hz, 2H); 7.54 (ddd, *J* = 8.2, 6.8, 1.2 Hz, 2H); 7.53 (d, *J* = 8.7 Hz, 2H); 7.33 (ddd, *J* = 8.4, 6.8, 1.2 Hz, 2H); 7.25 (ddd, *J* = 8.2, 6.7, 1.2 Hz, 2H); 7.13 (d, *J* = 8.7 Hz, 2H); 7.09 (d, *J* = 8.7 Hz, 2H); 6.53 (d, *J* = 8.4 Hz, 2H); 4.81 (d, *J* = 14.0 Hz, 2H); 4.64 (d, *J* = 14.0 Hz, 2H); 4.45 (d, *J* = 13.8 Hz, 2H); 3.73 (d, *J* = 13.7 Hz, 2H) ppm. ^13^C-NMR δ: 151.98 (dm, *J*_CF_ = 251 Hz, C); 140.29 (dt, *J*_CF_ = 257, 15.2 Hz, C); 139.54 (C); 136.65 (C); 136.24 (C); 135.73 (m, C); 134.16 (C); 133.80 (C); 132.99 (CH); 131.62 (C); 131.26 (C); 128.72 (CH); 128.67 (CH); 128.53 (CH); 128.39 (CH); 128.16 (CH); 127.83 (CH); 127.50 (CH); 127.47 (CH); 127.20 (CH); 126.60 (CH); 124.85 (C); 122.11 (C); 115.35 (m, CH); 62.62 (CH_2_); 57.31 (CH_2_) ppm. HRMS (ESI) calculated for C_56_H_34_F_6_N [M − Br]^+^: 834.2590, found 834.2595.

*(S,S)-2,6-Bis(3,5-bis(trifluoromethyl)phenyl)-3,3’,5,5’-tetrahydro-4,4’-spirobi[dinaphtho[2,1-c:1’,2’-e]azepin]-4-ium bromide* (**1h**) [47]: 62% yield. ^1^H-NMR δ: ~8.4–9.4 (br.s, ~2H); 8.33 (s, 2H); 8.25 (s, 2H); 8.16 (d, *J* = 8.3 Hz, 2H); 7.84 (d, *J* = 8.3 Hz, 2H); 7.69 (ps.t, *J* = 7.3 Hz, 2H); 7.51 (ps.t, *J* = 7.7 Hz, 2H); 7.41 (br.ddd, *J* = 8.3, 6.8, 1.0 Hz, 2H); 7.24 (d, *J* = 8.9 Hz, 2H); 7.23 (br.ddd, *J* = 7.9, 6.6, 1.0 Hz, 2H); 7.19 (d, *J* = 8.7 Hz, 2H); 7.08 (d, *J* = 8.5 Hz, 2H); 6.24 (d, *J* = 8.7 Hz, 2H); 4.83 (d, *J* = 13.9 Hz, 2H); 4.64 (d, *J* = 14.1 Hz, 2H); 4.54 (d, *J* = 13.9 Hz, 2H); 3.67 (d, *J* = 13.7 Hz, 2H) ppm. ^13^C-NMR δ: 141.96 (C); 139.76 (C); 136.52 (C); 136.06 (C); 134.00 (CH); 133.92 (C); 133.90 (C); 131.94 (C); 131.11 (C); 128.97 (CH); 128.80 (CH); 128.58 (CH); 128.54 (CH); 128.24 (CH); 127.86 (CH); 127.63 (CH); 127.42 (CH); 127.24 (CH); 125.91 (CH); 124.38 (C); 122.35 (br.CH); 121.76 (C); 62.77 (CH_2_); 57.45 (CH_2_) ppm. HRMS (ESI) calculated for C_60_H_36_F_12_N [M − Br]^+^: 998.2651, found 998.2644.

*(S,S)-2,6-Di(furan-2-yl)-3,3’,5,5’-tetrahydro-4,4’-spirobi[dinaphtho[2,1-c:1’,2’-e]azepin]-4-ium bromide* (**1i**): 81% yield; mp: >200 °C (dec.), [α]_D_^20^ = +288 (c: 0.58, DCM). ^1^H-NMR δ: 8.50 (s, 2H); 8.11 (d, *J* = 8.2 Hz, 2H); 7.95 (d, *J* = 8.2 Hz, 2H), 7.75 (d, *J* = 8.5 Hz, 2H); 7.72 (dd, *J* = 1.8, 0.4 Hz, 2H); 7.62 (ddd, *J* = 8.2, 6.9, 1.2 Hz, 2H); 7.54 (ddd, *J* = 8.0, 6.6, 1.3 Hz, 2H); 7.32 (ddd, *J* = 8.2, 6.7, 1.1 Hz, 2H); 7.25 (m, 4H); 7.19 (br.d, *J* = 8.7 Hz, 2H); 7.10 (br.d, *J* = 8.6 Hz, 2H); 6.90 (dd, *J* = 3.4, 1.8 Hz, 2H); 6.49 (d, *J* = 8.5 Hz, 2H); 5.36 (d, *J* = 14.4 Hz, 2H); 4.20 (d, *J* = 14.4 Hz, 2H); 4.19 (d, *J* = 13.7 Hz, 2H); 3.91 (d, *J* = 13.7 Hz, 2H) ppm. ^13^C-NMR δ: 152.05 (C); 144.12 (CH); 138.96 (C); 136.28 (C); 134.01 (C); 133.88 (C); 131.20 (C); 130.95 (C); 130.85 (CH); 130.14 (CH); 128.72 (CH); 128.56 (CH); 128.32 (CH); 128.22 (C); 127.96 (CH); 127.69 (CH); 127.60 (CH); 127.29 (CH); 126.96 (CH); 126.94 (CH); 125.16 (C); 121.93 (C); 113.15 (CH); 112.11 (CH); 62.35 (CH_2_); 57.13 (CH_2_) ppm. HRMS (ESI) calculated for C_52_H_36_NO_2_ [M − Br]^+^: 706.2741, found: 706.2745.

*(S,S)-2,6-Di(thiophen-2-yl)-3,3’,5,5’-tetrahydro-4,4’-spirobi[dinaphtho[2,1-c:1’,2’-e]azepin]-4-ium bromide* (**1j**): 88% yield; mp: 245–247 °C; [α]_D_^20^ = +124 (c: 0.50, DCM). ^1^H-NMR δ: 8.41 (s, 2H); 8.08 (d, *J* = 8.2 Hz, 2H); 7.89 (d, *J* = 8.2 Hz, 2H); 7.74 (dd, *J* = 5.3, 0.9 Hz, 2H); 7.62 (ddd, *J* = 7.9, 6.7, 0.9 Hz, 2H); 7.59 (d, *J* = 8.5 Hz, 2H); 7.51 (ddd, *J* = 7.9, 6.7, 0.9 Hz, 2H); 7.50 (br.s, 2H); 7.38 (dd, *J* = 5.1, 3.7 Hz, 2H); 7.32 (ddd, *J* = 7.9, 6.7, 1.0 Hz, 2H); 7.22 (ddd, *J* = 7.9, 6.6, 1.0 Hz, 2H); 7.15 (d, *J* = 8.8 Hz, 2H); 7.08 (d, *J* = 8.6 Hz, 2H); 6.47 (d, *J* = 8.5 Hz, 2H); 5.36 (d, *J* = 14.1 Hz, 2H); 4.36 (d, *J* = 14.0 Hz, 2H); 4.22 (d, *J* = 13.6 Hz, 2H); 3.90 (d, *J* = 13.4 Hz, 2H) ppm. ^13^C-NMR δ: 140.98 (C); 139.25 (C); 136.34 (C); 133.99 (C); 133.94 (C); 132.73 (CH); 131.77 (C); 131.12 (C); 131.00 (C); 130.59 (CH); 130.02 (CH); 129.30 (CH); 128.56 (CH); 128.51 (CH); 128.24 (CH); 127.80 (CH); 127.68 (CH); 127.48 (2CH); 127.45 (CH); 126.85 (CH); 126.76 (CH); 125.24 (C); 122.66 (C); 62.66 (CH_2_); 56.92 (CH_2_) ppm. HRMS (ESI) calculated for C_52_H_36_NS_2_ [M − Br]^+^: 738.2284, found: 738.2254.

*(S)-2,6-Bis(trimethylsilyl)-4,5-dihydro-3H-dinaphtho[2,1-c:1′,2′-e]azepine* (**4**): To a suspension of dibromide **3** [39] (380 mg, 0.65 mmol) in acetonitrile (25 mL) was added aqueous ammonia (25%, 10 mL) and the mixture was stirred in a pressure tube at 65–70 °C (bath) overnight. The conversion was followed by TLC. Excess of ammonia was evaporated and DCM (40 mL) was added. The organic phase was washed with water and brine, dried (Na_2_SO_4_), and evaporated. Purification of the residual oil by column chromatography on silica gel (EtOAc (5→20)/heptane) afforded 243 mg (85%) of **4** as a colourless foam; [α]_D_^20^ = +416 (c: 1.00, DCM). ^1^H-NMR δ: 8.15 (s, 2H); 7.93 (br.d, *J* = 8.2 Hz, 2H); 7.43 (ddd, *J* = 8.1, 6.3, 1.2 Hz, 2H); 7.28 (br.d, *J* = 8.1 Hz, 2H); 7.22 (ddd, *J* = 8.1, 6.6, 1.2 Hz, 2H); 4.09 (d, *J* = 12.8 Hz, 2H); 3.47 (d, *J* = 12.8 Hz, 2H); 1.78 (br.s, 1H); 0.50 (s, 18 H) ppm. ^13^C-NMR δ: 138.77 (C); 136.50 (C); 136.11 (CH); 135.00 (C); 131.98 (C); 131.96 (C); 128.35 (CH); 127.31 (CH); 126.07 (CH); 125.20 (CH); 47.43 (CH_2_); 0.55 (CH_3_) ppm. HRMS (ESI) calculated for C_28_H_34_NSi_2_ [M + H]^+^: 440.2224, found: 440.2226.

*(S)-2′,6′-Bis(trimethylsilyl)-3′,5,5′,7-tetrahydrospiro[dibenzo[c,e]azepine-6,4′-dinaphtho[2,1-c:1′,2′-e]azepin]-6-ium* bromide (**5′**): 72% yield; [α]_D_^20^ = +62 (c: 1.00, DCM). ^1^H-NMR δ: 8.29 (s, 2H); 8.02 (dm, *J* = 8.3 Hz, 2H); 7.71–7.76 (m, 4H); 7.57-7.63 (m, 4H); 7.34 (ddd, *J* = 8.5, 6.9, 1.3 Hz, 2H); 7.19 (dm, *J* = 8.4 Hz, 2H); 4.77 (d, *J* = 13.3 Hz, 2H); 4.70 (d, *J* = 13.3 Hz, 2H); 4.39 (d, *J* = 12.7 Hz, 2H); 3.99 (d, *J* = 12.6 Hz, 2H); 0.22 (s, 18H) ppm. ^13^C-NMR δ: 140.94 (C); 138.62 (CH); 138.05 (C); 136.88 (C); 133.42 (C); 131.96 (C); 131.86 (CH); 131.60 (CH); 129.75 (CH); 128.83 (CH); 128.79 (C); 128.75 (CH); 127.93 (CH); 127.78 (CH); 127.31 (CH); 126.72 (C); 63.74 (CH_2_); 63.74 (CH_2_); 1.72 (CH_3_) ppm. HRMS (ESI) calculated for C_42_H_44_NSi_2_ [M − Br]^+^: 618.3007, found: 618.3020.

*Optical resolution of rac*-**7**: To a solution of 547 mg (1.0 mmol) of azepine (±)-**7** [29] in 12 mL of DCM and 12 mL of MeOH was added slowly, with gentle mixing, a solution of 93 mg (0.26 mmol) of (+)-(*S,S*)-*O,O*-dibenzoyltartaric acid monohydrate in 5 mL of MeOH at room temperature. The mixture was left undisturbed for 6–8 h and then kept at +4 °C overnight. The crystalline material containing (*S,S*)-(*R*)_ax_ salt was separated from the mother liquor which was subsequently evaporated. The fractions were stirred in a mixture of DCM/NaOH (2M). The organic phases were separated and dried (Na_2_SO_4_) to yield 236 mg (43%) of (*R*)-**7** with 89% ee from the less soluble salt and 290 mg (53%) of (S)-**7** with 77%ee from the mother liquor. Enantiomerically enriched (*R*)-**7** was recrystallized from DCM (10 mL)/MeOH (6 mL) to yield 210 mg (38%) of (*R*)-**7** with >99%ee Combined mother liquors containing (*S*)-enriched **7** were evaporated and treated with 0.5 equiv. of (-)-(*R,R*)-*O,O*-dibenzoyltartaric acid monohydrate in MeOH/DCM as before to yield 68% of (*S*)-**7** with 89% ee. Recrystallisation afforded enantiopure (*S*)-**7**. The enantiomeric excess was determined by chiral HPLC analysis (Chiralpak ADH, 2-PrOH/heptane (20:80), 20 °C, 0.5 mL min^-1^; 14.3 min (*R*), 21.6 min (*S*)); mp: 261 °C for (*S*)-**7**.

*tert-Butyl (S)-2,6-diiodo-3,5-dihydro-4H-dinaphtho[2,1-c:1’,2’-e]azepine-4-carboxylate* (**8**): A solution of (*S*)-**7** (558 mg, 1 mmol) in DCM (6 mL) was added to a suspension of amberlyst-15 (56 mg) and boc-anhydride (436 mg, 1 mmol) in EtOH (3 mL) and stirred at r.t. for 10 min. The reaction mixture was filtered and evaporated. The remaining solid was repeatedly leached with pentane/ether to remove excess of boc-anhydride leaving **8** as an off-white solid, sufficiently pure for the next step (yield: 546 mg, 84%, purity 98% by NMR). ^1^H-NMR (600 MHz) δ: 8.60 (br.s, 2H); 7.81 (d, *J* = 8.1 Hz, 2H); 7.48 (ps.t, *J* ~7.5 Hz, 2H); 7.20–7.29 (m, 4H); 5.64 (br.d, *J* = 13.1 Hz, 1H); 5.49 (br.d, *J* = 13.5 Hz, 1H); 3.59 (br.d, *J* = 13.8 Hz, 1H); 3.50 (br.d, *J* = 13.8 Hz, 1H); 1.57 (s, 9H) ppm. ^13^C-NMR δ: 153.14 (C); 139.99 (CH); 136.11 (br.C); 136.00 (br.C); 134.98 (br.C); 134.62 (br.C); 134.27 (C); 130.76 (C); 127.38 (CH); 127.13 (CH); 126.84 (br.CH); 126.79 (br.CH); 97.90 (C); 97.63 (C); 80.28 (C); 51.60 (CH_2_); 50.88 (CH_2_); 28.49 (CH_3_) ppm. HRMS (ESI) calculated for C_23_H_16_I_2_NO_2_ [M − C_4_H_8_ + H]^+^: 591.9265, found: 591.9260.

*Suzuki-Miyaura coupling of (S)-***7***yielding (S*)-**9** (*General Procedure B*)*:* A Schlenk tube, equipped with magnetic stirring bar and glass stopper, was charged with a solution of diiodoazepine (274 mg, 0.50 mmol) in toluene (10 mL) and Na_2_CO_3_ solution (2 M in H_2_O, 5.0 mL). Then arylboronic acid (2.00 mmol, 4 equiv.) was added and the mixture was degassed. After the addition of Pd(PPh_3_)_4_ (115 mg, 20 mol %), the reaction was left stirring at 80 °C for 6–48 h. The conversion was monitored by TLC (EtOAc/heptane, 30:70). After cooling to r.t., DCM (50 mL) and H_2_O (30 mL) were added and the phases were separated. The aqueous phase was extracted with DCM (3 × 20 mL). The combined organic phases were washed with KOH solution (10%, 20 mL) and sat. NaCl solution and dried (K_2_CO_3_). After evaporation of solvents, the crude material was purified by MPLC using a solvent gradient (EtOAc + 5% triethylamine (0→30%)/heptane).

*Suzuki–Miyaura coupling of (S)-***8***yielding (S)*-**9***(General Procedure C)*: A Schlenk tube, equipped with magnetic stirring bar, was charged with a solution of boc-protected diiodoazepine **8** (97 mg, 0.15 mmol) and tri-*ortho*-tolylphosphine (9.1 mg, 20 mol %) in toluene (3 mL) and a Na_2_CO_3_ solution (2 M in H_2_O, 2 mL). Then, arylboronic acid (4 equiv.) was added and the mixture was degassed. After the addition of Pd(OAc)_2_ (3.4 mg, 10 mol %), the mixture was left stirring at 80 °C for 12–48 h. The reaction was monitored by TLC (EtOAc/heptane, 30:70). After cooling to r.t., DCM (5 mL) and H_2_O (3 mL) were added and the phases were separated. The aqueous phase was extracted with DCM (3 × 3 mL) and the combined organic phases were washed with KOH solution (10%, 5 mL) and sat. NaCl solution and dried (Na_2_SO_4_). After evaporation of solvents DCM (1 mL) and trifluoroacetic acid (1 mL) was added and the solution stirred at r.t. for 2 h. The reaction was carfully neutralized with solid NaHCO_3_. Extractive work-up was followed by MPLC.

*(S)-2,6-Diphenyl-4,5-dihydro-3H-dinaphtho[2,1-c:1′,2′-e]azepine* (**9a**) [53]: 77% yield [29] (*General Procedure B*).

*(S)-2,6-Di(naphthalen-2-yl)-4,5-dihydro-3H-dinaphtho[2,1-c:1′,2′-e]azepine* (**9b**): 68% yield [29] (*General Procedure B*).

*(S)-2,6-Di([1,1′-biphenyl]-4-yl)-4,5-dihydro-3H-dinaphtho[2,1-c:1′,2′-e]azepine* (**9c**): 68% yield [29] (*General Procedure B*).

*(S)-2,6-Di([1,1’:4’,1’’-terphenyl]-4-yl)-4,5-dihydro-3H-dinaphtho[2,1-c:1’,2’-e]azepine* (**9d**): 55% yield (*General Procedure B* was modified using (dppf)PdCl_2_, 10 mol %); mp: >260 °C (dec.); [α]_D_^20^ = +247 (c: 0.60, DCM). ^1^H-NMR δ: 8.02 (s, 2H); 7.98 (br.d, *J* = 8.2 Hz, 2H); 7.65–7.79 (m, 20H); 7.44–7.53 (m, 8H); 7.37 (m, 2H); 7.33 (ddd, *J* = 8.6, 6.7, 1.3 Hz, 2H); 4.11 (d, *J* = 12.6 Hz, 2H); 3.43 (d, *J* = 12.6 Hz, 2H) ppm. ^13^C-NMR δ: 140.69 (C); 140.44 (C); 140.23 (C); 139.68 (C); 139.55 (C); 139.35 (C); 136.13 (C); 133.36 (C); 132.53 (C); 130.89 (C); 130.19 (CH); 129.67 (CH); 128.82 (CH); 128.32 (CH); 127.57 (CH); 127.55 (CH); 127.46 (CH); 127.36 (CH); 127.05 (CH); 126.83 (CH); 125.84 (CH); 125.79 (CH); 44.67 (CH_2_) ppm. HRMS (ESI) calculated for C_58_H_42_N [M + H]^+^: 752.3317, found: 752.3289.

*(S)-2,6-Bis(4-tritylphenyl)-4,5-dihydro-3H-dinaphtho[2,1-c:1’,2’-e]azepine* (**9e**): 89% yield, white solid; (*General Procedure B* was modified using (dppf)PdCl_2_, 10 mol %); mp: 280–290 °C; [α]_D_^20^ = +206 (c: 0.84, DCM). ^1^H-NMR δ: 7.97 (s, 2H); 7.92 (br.d, *J* = 8.0 Hz, 2H); 7.43–7.49 (m, 6H); 7.41 (br.d, *J* = 8.6 Hz, 2H); 7.17–7.33 (m, ~36H); 4.03 (d, *J* = 12.4 Hz, 2H); 3.33 (d, *J* = 12.4 Hz, 2H) ppm. ^13^C-NMR δ: 146.76 (C); 145.78 (C); 139.41 (C); 138.81 (C); 136.02 (C); 133.36 (C); 132.47 (C); 131.19 (CH); 131.00 (CH); 130.79 (C); 129.62 (CH); 128.67 (CH); 128.22 (CH); 127.51 (2CH); 125.96 (CH); 125.74 (CH); 125.65 (CH); 64.86 (C); 44.63 (CH_2_) ppm. HRMS (ESI) calculated for C_72_H_54_N [M + H]^+^: 932.4251, found: 932.4217.

*(S)-2,6-Bis(4-fluorophenyl)-4,5-dihydro-3H-dinaphtho[2,1-c:1’,2’-e]azepine* (**9f**): 49% yield (*General Procedure B*); 60% (*General Procedure C*); solidifying oil, [α]_D_^20^ = +241 (c: 1.01, DCM). ^1^H-NMR δ: 7.94 (dm, *J* = 8.4 Hz, 2H); 7.92 (s, 2H); 7.56 (m, 4H); 7.48 (ddd, *J* = 8.0, 6.7, 1.1 Hz, 2H); 7.42 (dm, *J* = 8.6 Hz, 2H); 7.28 (ddd, *J* = 8.3, 6.7, 1.3 Hz, 2H); 7.16 (m, 4H); 3.94 (d, *J* = 12.6, 2H); 3.33 (d, *J* = 12.6 Hz, 2H) ppm. ^13^C-NMR δ: 162.32 (d, *J*_CF_ = 245 Hz, CF); 138.64 (C); 137.22 (d, *J*_CF_ = 2.3 Hz, C); 136.03 (C); 133.20 (C); 132.40 (C); 131.20 (d, *J*_CF_ = 8.0 Hz, CH); 130.80 (C); 129.72 (CH); 128.24 (CH); 127.48 (CH); 125.88 (d, *J*_CF_ = 7.5 Hz, CH); 115.17 (CH); 115.03 (CH); 44.52 (CH_2_) ppm. HRMS (ESI) calculated for C_34_H_24_F_2_N [M + H]^+^: 484.1877, found 484.1877.

*(S)-2,6-Bis(3,4,5-trifluorophenyl)-4,5-dihydro-3H-dinaphtho[2,1-c:1’,2’-e]azepine* (**9g**): 80% yield *(General Procedure C)*; solidifying oil, [α]_D_^20^ = +244 (c: 0.95, DCM). ^1^H-NMR δ: 7.96 (br.d, *J* = 8.0 Hz, 2H); 7.92 (s, 2H); 7.52 (ddd, *J* = 8.0, 7.6, 1.3 Hz, 2H); 7.41 (dm, *J* = 8.1 Hz, 2H); 7.31 (ddd, *J* = 8.0, 6.8, 1.3 Hz, 2H); 7.29 (br.m, 4H); 3.94 (d, *J* = 12.8 Hz, 2H); 3.33 (d, *J* = 12.8 Hz, 2H); 7.79 (br.s, ~1H) ppm. ^13^C-NMR δ: 150.86 (ddd, *J*_CF_ = 250, 10, 4 Hz, C); 139.26 (dt, *J*_CF_ = 255, 15 Hz, C); 137.16 (td, *J*_CF_ = 8, 5 Hz, C); 136.74 (C); 136.18 (C); 132.46 (C); 132.30 (C); 131.03 (C); 129.85 (CH); 128.39 (CH); 127.40 (CH); 126.46 (CH); 126.30 (CH); 113.91 (m, CH); 44.43 (CH_2_) ppm. HRMS (ESI) calculated for C_34_H_20_F_6_N [M + H]^+^: 556.1500, found: 556.1496.

*(S)-2,6-Bis(3,5-bis(trifluoromethyl))phenyl)-4,5-dihydro-3H-dinaphtho[2,1-c:1’,2’-e]azepine* (**9h)**: 29% yield (*General Procedure B*); 75% yield *(General Procedure C)*. solidifying oil, [α]_D_^20^ = +207 (c: 0.99, DCM). ^1^H-NMR δ: 8.12 (br.s, 4H); 7.99 (dm, *J* = 8.3 Hz, 2H); 7.98 (s, 2H); 7.94 (m, 2H); 7.54 (ddd, *J* = 8.1, 6.7, 1.2 Hz, 2H); 7.44 (dm, *J* = 8.7 Hz, 2H); 7.34 (ddd, *J* = 8.5, 6.8, 1.4 Hz, 2H); 3.85 (d, *J* = 12.8 Hz, 2H); 3.39 (d, *J* = 12.9, 2H) ppm. ^13^C-NMR δ: 143.18 (C); 136.54 (C); 136.32 (C); 132.34 (C); 132.32 (C); 131.65 (q, J_CF_ = 33.4 Hz, CCF_3_); 131.17 (C); 130.30 (CH); 129.88 (br.CH); 128.47 (CH); 127.38 (CH); 126.75 (CH); 126.47 (CH); 123.26 (q, J_CF_ = 274 Hz, CCF_3_); 121.18 (m, CH); 44.48 (CH_2_) ppm. HRMS (ESI) calculated for C_38_H_22_F_12_N [M + H]^+^: 720.1561, found 720.1553.

*(S)-2,6-Di(furan-2-yl)-4,5-dihydro-3H-dinaphtho[2,1-c:1’,2’-e]azepine* (**9i**): 98% yield (*General Procedure B*); colorless foam. ^1^H-NMR δ: 8.20 (s, 2H); 7.95 (br.d, *J* = 8.2 Hz, 2H); 7.61 (dd, *J* = 1.8, 0.7 Hz, 2H); 7.47 (ddd, *J* = 8.0, 6.7, 1.2 Hz, 2H); 7.37 (dm, *J* = 8.6 Hz, 2H); 7.25 (ddd, *J* = 8.3, 6.7, 1.3 Hz, 2H); 6.77 (dd, *J* = 3.4, 0.7 Hz, 2H); 6.57 (dd, *J* = 3.3, 1.8 Hz, 2H); 4.34 (d, *J* = 12.6 Hz, 2H); 3.36 (d, *J* = 12.6 Hz, 2H) ppm. ^13^C-NMR δ: 153.69 (C); 142.59 (CH); 136.31 (C); 132.51 (C); 132.47 (C); 130.88 (C); 129.02 (CH); 128.49 (CH); 128.35 (C); 127.49 (CH); 126.18 (CH); 125.92 (CH); 111.46 (CH); 109.18 (CH); 44.80 (CH_2_) ppm. HRMS (ESI) calculated for C_30_H_22_NO_2_ [M + H]^+^: 428.1651, found: 428.1647.

*(S)-2,6-Di(thiophen-2-yl)-4,5-dihydro-3H-dinaphtho[2,1-c:1’,2’-e]azepine* (**9j**): 84% yield (*General Procedure B*); colorless foam. ^1^H-NMR δ: 8.09 (s, 2H); 7.94 (d, *J* = 8.3 Hz, 2H); 7.48 (ddd, *J* = 8.0, 6.9, 1.0 Hz, 2H); 7.41 (m, 4 H); 7.31 (dd, *J* = 3.5, 1.1 Hz, 2H); 7.28 (ddd, *J* = 8.0, 6.7, 1.1 Hz, 2H); 7.15 (dd, *J* = 5.2, 3.5 Hz, 2H); 4.25, (d, *J* = 12.6 Hz); 3.34 (d, *J* = 12.6 Hz) ppm. ^13^C-NMR δ: 142.22 (C); 136.08 (C); 133.68 (C); 132.29 (C); 131.90 (C); 130.92 (C); 130.87 (CH); 128.33 (CH); 127.46 (CH); 127.42 (CH); 127.38 (CH); 126.14 (CH); 125.98 (CH); 125.60 (CH); 44.56 (CH_2_) ppm. HRMS (ESI) calculated for C_30_H_22_NS_2_ [M + H]^+^: 460.1194, found: 460.1185.

*tert-Butyl (S)-2,6-bis(4,4,5,5-tetramethyl-1,3,2-dioxaborolan-2-yl)-3,5-dihydro-4H-dinaphtho[2,1-c: 1’,2’-e]azepine-4-carboxylate* (**11**): A suspension of **8** (324 mg, 0.5 mmol), B_2_Pin_2_ (508 mg, 2 mmol), and KOAc (295 mg, 3 mmol) in DMF was degassed. Pd(OAc)_2_ (11 mg, 0.05 mmol) was added and the mixture was degassed once more and stirred for 20 h at 100 °C (bath). Solvent was removed under reduced pressure and the residue was taken up in DCM (100 mL)/water (100 mL). The aqueous layer was extracted with DCM and combined organics were washed with water and sat. NH_4_Cl (50 mL). The organic phase was dried over MgSO_4_, concentrated and subjected to chromatography (EtOAc (0→20%)/heptane; yield 66%, pale yellow oil. The material contained 5–10% of B_2_Pin_2_ and was used without further purification. ^1^H-NMR δ: 8.49 (br.s, 2H); 7.95 (d, *J* = 8.2 Hz, 2H); 7.42 (m, 2H); 7.20–7.26 (m, 4H); 5.52–6.03 (br.m, 2H); 3.45 (br.d, *J* = 12.8 Hz, 2H); 1.47 (s, 12H); 1.46 (br.s, ~9H); 1.43 (s, 12H) ppm. ^13^C-NMR δ: 154.10 (C); 136.68 (C); 132.68 (br. C); 132.04 (C); 128.67 (CH); 127.24 (CH); 126.80 (CH); 125.48 (CH); 84.08 (br. CH_2_); 83.43 (C); 78.69 (C); 28.47 (CH_3_); 24.98 (CH_3_); 24.89 (CH_3_) ppm. HRMS (ESI) calculated for C_39_H_48_B_2_NO_6_ [M + H]^+^: 648.3668, found: 648.3680.

*Phase-transfer catalyzed α-substitution of tert-butyl 2-((diphenylmethylene)amino)acetate **12** (Typical procedure with benzylbromide **13A**): Stock solution A:* 0.025 mmol catalyst in 1 mL DCM, 2.5 × 10^−2^ M; *Stock solution B:* 1.480 g of *tert*-butyl 2-((diphenylmethylene)amino)acetate **12** in 30 mL of toluene, 1.667 × 10^−1^ M; *Stock solution C:* 10 g of solid KOH in 10 g distilled water. A 10 mL Schlenk tube with stirring bar was subsequently charged with 100 µL of solution A (0.0025 mmol, 1 mol %), 1.500 mL of solution B (1.310 g; d = 0.873, 0.25 mmol) and 0.5 mL of solution C. The mixture was degassed and cooled to 0 °C. Freshly distilled benzyl bromide was added with a microliter syringe (38 µL, 54 mg, 1.2 equiv.) and the reaction was vigorously stirred for 4 h. Et_2_O (5 mL) and water (3 mL) was added with stirring and the phases were separated. The aqueous phase was extracted with Et_2_O (3× 5 mL) and the organic layer was washed with water (5 mL) and brine and dried (MgSO_4_). Evaporation gave a clear oil to which solid bibenzyl (0.25 mmol, 45.6 mg) was added and the mixture was completely dissolved in CDCl_3_ (approx. 1 mL). A ^1^H-NMR spectrum was recorded (400 MHz). Integration gave %yield of product, substrate, and eventually benzophenone as a side product. An aliquot (20–30 µL) was transferred to a HPLC vial and diluted with 2-PrOH to 1 mL. Chiral HPLC analysis applying reported conditions (Chiralcel ODH (250 × 4.6 mm), 2-PrOH/heptane 1:99, 0.5 mL min^−1^, 25 °C) gave satisfying separation of enantiomers (13.5 min (*R*)-**14A**, 23.6 min (*S*)-**14A**) without overlap with internal standard (10.7 min), substrate (19.8 min) or benzophenone (17.6 min). Alternatively, the reaction mixture was subjected to MPLC (10 g of SiO_2_, solvent gradient EtOAc (0%→8%)/heptane) to yield a pure product.

*tert-Butyl 2-((diphenylmethylene)amino)-3-(pyridin-2-yl)propanoate* (**14G**): colorless oil. ^1^H-NMR δ = 8.50 (dm, *J* = 4.9 Hz, 1H); 7.63 (m, 2H); 7.59 (td, *J* = 7.6, 1.9 Hz, 1H); 7.41–7.45 (m, 2H); 7.35–7.40 (m, 4 H); 7.25 (dm, *J* = 7.6 Hz, 1H); 7.15 (ddd, *J* = 7.5, 4.9, 1.2 Hz, 1H); 6.78 (br.d, *J* = 6.8 Hz, 2H); 4.53 (dd, *J* = 9.5, 3.9 Hz, 1H); 3.52 (dd, *J* = 13.4, 4.0 Hz, 1H); 3.45 (dd, *J* = 13.3, 9.4 Hz, 1H); 1.52 (s, 9 H) ppm. ^13^C-NMR δ: 170.66 (C); 170.64 (C); 158.61 (C); 149.12 (CH); 139.52 (C); 136.15 (C); 135.87 (CH); 130.03 (CH); 128.67 (CH); 128.34 (CH); 128.01 (CH); 127.83 (CH); 127.61 (CH); 124.50 (CH); 121.14 (CH); 81.08 (C); 66.28 (CH); 41.91 (CH_2_); 27.95 (CH_3_) ppm. HRMS: calcd for C_25_H_27_N_2_O_2_ [M + H]^+^: 387.2073; found: 387.2068. HPLC: Chiralpak ADH (250 × 4.6 mm), 2-PrOH/heptane (5:95), 0.5 mL min^−1^, 25 °C, t_R_ = 13.93 min, 17.55 min.

*6-(tert-Butyl) 1-ethyl (E)-5-((diphenylmethylene)amino)hex-2-enedioate* (**14H**): colorless oil. ^1^H-NMR δ: 7.62–7.65 (m, 2H); 7.41–7.47 (m, 3H); 7.37–7.41 (m, 1H); 7.30–7.34 (m, 2H); 7.15–7.18 (m, 2H); 6.82 (dt, *J* = 15.6, 7.6 Hz, 1H); 5.84 (dm, *J* = 15.6 Hz, 1H); 4.16 (qm, *J* = 7.1 Hz, 2H); 4.07 (dd, *J* = 7.6, 5.2 Hz, 1H); 2.71–2.82 (m, 2H); 1.44 (s, 9H); 1.26 (t, *J* = 7.1 Hz, 3H) ppm. ^13^C-NMR δ = 170.82 (C); 170.18 (C); 145.02 (CH); 139.37 (C); 136.39 (C); 130.35 (CH); 128.81 (CH); 128.63 (CH); 128.48 (CH); 128.00 (CH); 127.76 (CH); 123.58 (CH); 81.47 (C); 64.94 (CH); 60.13 (CH_2_); 36.26 (CH_2_); 28.00 (CH_3_); 14.23 (CH_3_) ppm. HRMS: calculated for C_25_H_30_NO_4_ [M + H]^+^: 408.2175; found: 408.2171. HPLC: Chiralpak ADH (250 × 4.6 mm), 2-PrOH/heptane (5:95), 0.5 mL min^−1^, 25 °C, t_R_ = 9.69 min, 12.42 min.

*tert-Butyl (E)-6-(1,3-dioxoisoindolin-2-yl)-2-((diphenylmethylene)amino)hex-4-enoate* (**14I**): colorless oil. ^1^H-NMR δ: 7.73–7.78 (m, 2H); 7.64–7.69 (m, 2H); 7.53–7.57 (m, 2H); 7.38–7.44 (m, 3H); 7.26–7.30 (m, 1H); 7.16–7.23 (m, 4H); 5.59–5.62 (m, 2H); 4.15–4.25 (m, 2H); 3.94 (t, *J* = 6.7 Hz, 1H); 2.62 (m, 2H); 1.41 (s, 9H) ppm. ^13^C-NMR δ: 170.66 (C); 170.37 (C); 167.79 (C); 139.59 (C); 136.69 (C); 133.78 (CH); 132.14 (C); 130.85 (CH); 130.04 (CH); 128.67 (CH); 128.47 (CH); 128.38 (CH); 127.95 (CH); 127.83 (CH); 126.14 (CH); 123.13 (CH); 81.06 (C); 65.63 (CH); 39.37 (CH_2_); 36.51 (CH_2_); 28.02 (CH_3_) ppm. HRMS: calcd for C_31_H_31_N_2_O_4_ [M + H]^+^: 495.2284; found: 495.2283. HPLC: Chiralpak ADH (250 × 4.6 mm), 2-PrOH/heptane (5:95), 1.0 mL min^−1^, 25 °C, t_R_ = 13.11 min, 15.45 min.

*Ethyl 2-(2-(tert-butoxy)-1-((diphenylmethylene)amino)-2-oxoethyl)cyclopropane-1-carboxylate* (**15**): colorless oil. ^1^H-NMR δ: 7.61–7.63 (m, 2H); 7.42–7.47 (m, 3H); 7.37–7.41 (m, 1H); 7.30–7.34 (m, 2H); 7.15–7.18 (m, 2H); 4.13 (q, *J* = 7.1 Hz, 2H); 3.79 (d, *J* = 5.8 Hz, 1H); 2.03 (dddd, *J* = 10.3, 6.4, 5.9, 4.2 Hz, 1H); 1.83 (ddd, *J* = 9.5, 5.1, 4.3 Hz, 1H); 1.44 (s, 9H); 1.26 (t, *J* = 7.1 Hz, 3H); 1.18 (ddd, *J* = 9.5, 5.1, 4.3 Hz, 1H); 0.91 (ddd, *J* = 8.4, 6.4, 4.3 Hz, 1H) ppm. ^13^C-NMR δ: 174.06 (C); 170.77 (C); 170.24 (C); 139.33 (C); 136.27 (C); 130.38 (CH); 128.82 (CH); 128.64 (CH); 128.49 (CH); 127.97 (CH); 127.77 (CH); 81.28 (C); 65.44 (CH); 60.36 (CH_2_); 27.98 (CH_3_); 25.13 (CH); 17.03 (CH); 14.26 (CH_3_); 12.02 (CH_2_) ppm. HRMS: calculated for C_25_H_30_NO_4_ [M + H]^+^: 408.2175; found: 408.2177.

*2-([1,1’:4’,1’’-Terphenyl]-4-yl)-4,4,5,5-tetramethyl-1,3,2-dioxaborolane* [54]: A literature procedure was followed [52]; 65% yield. ^1^H-NMR δ: 7.93 (dm, *J* = 8.3 Hz, 2H); 7.64–7.75 (m, 8H); 7.48 (m, 2H); 7.37 (m, 1H); 1.39 (s, 12H) ppm. ^13^C-NMR δ: 143.32 (C); 140.66 (C); 140.43 (C); 139.88 (C); 135.31 (CH); 128.80 (CH); 127.55 (CH); 127.49 (CH); 127.36 (CH); 127.04 (CH); 126.30 (CH); 83.81 (C); 24.88 (CH_3_) ppm. HRMS (ESI) calculated for C_24_H_25_BNaO_2_ [M + Na]^+^: 379.1845, found 379.1843.

*4,4,5,5-Tetramethyl-2-(4-tritylphenyl)-1,3,2-dioxaborolane* [52]: ^1^H-NMR δ: 7.69 (dm, *J* = 8.5 Hz, 2H); 7.15–7.27 (m, ~17H); 1.33 (s, 12H) ppm. ^13^C-NMR δ: 150.00 (C); 146.64 (C); 133.94 (CH); 131.13 (CH); 130.54 (CH); 127.50 (CH); 125.89 (CH); 83.73 (C); 65.21 (C); 24.88 (CH_3_) ppm.

*Ethyl (E)-4-iodobut-2-enoate*: A literature procedure reported for the methylester was applied [55]. Yield: 77% (pale yellow oil, 10 mmol scale) The compound decomposed slowly at r.t. and was stored at -20 °C. ^1^H-NMR δ: 7.04 (dt, *J* = 15.3, 8.2 Hz, 1H); 5.93 (dt, *J* = 15.3, 1.0 Hz, 1H); 4.20 (q, *J* = 7.2 Hz, 2H); 3.93 (dd, *J* = 8.3, 1.1 Hz, 2H); 1.29 (t, *J* = 7.2 Hz, 3H) ppm. ^13^C-NMR δ: 143.44 (CH); 123.30 (CH); 60.67 (CH_2_); 14.19 (CH_3_); 0.72 (CH_2_) ppm. HRMS calculated for C_6_H_10_IO_2_ [M + H]^+^: 240.9725; found 240.9723.

*(E)-2-(4-Bromobut-2-en-1-yl)isoindoline-1,3-dione*: A literature procedure was applied [56]. ^1^H-NMR δ: 7.83-7.87 (m, 2H); 7.70-7.74 (m, 2H); 5.94 (dm, *J* = 15.4 Hz, 2H); 5.83 (dm, *J* = 15.3 Hz, 2H); 4.30 (dm, *J* = 5.7 Hz, 2H); 3.90 (dm, *J* = 7.2 Hz, 2H) ppm. ^13^C-NMR δ: 167.70 (C); 134.03 (CH); 132.02 (C); 129.95 (CH), 128.33 (CH); 123.34 (CH); 38.57 (CH_2_); 31.18 (CH_2_) ppm.

*(E)-2-(4-Iodobut-2-en-1-yl)isoindoline-1,3-dione* [57]: To a solution of (*E*)-2-(4-bromobut-2-en-1-yl)isoindoline-1,3-dione (560 mg, 2 mmol) in acetone (10 mL) was added NaI (1.50 g, 10 mmol) and the reaction was stirred at r.t. for 20 h. The mixture was concentrated and the residue was partitioned between EtOAc and water (50 + 50 mL). The organic phase was separated, concentrated under reduced presure, and the crude material subjected to chromatography ((EtOAc (5→25%)/heptane) to yield 486 mg (74%) of product as crystalline solid. ^1^H-NMR δ: 7.83–7.88 (m, 2H); 7.70–7.75 (m, 2H); 5.99 (dtt, *J* = 15.2, 7.8, 1.3 Hz, 1H); 5.76 (dtt, *J* = 15.2, 6.2, 1.0 Hz, 2H); 4.27 (dm, *J* = 6.2 Hz, 2H); 3.82 (dm, *J* = 7.9 Hz, 2H) ppm. ^13^C-NMR δ: 167.70 (C); 134.00 (CH); 131.99 (C); 131.63 (CH); 126.91 (CH); 123.31 (CH); 38.57 (CH_2_); 3.61 (CH_2_) ppm. HRMS calculatedfor C_12_H_10_INaNO_2_ [M + Na]^+^: 349.9654; found: 349.9645.

### 3.3. X-ray Structure Analysis

Crystals suitable for structure determinations were grown from DCM/heptane (**1d**, **1e**) or DCM/MeOH (during optical resolution of **7**). The X-ray intensity data were measured on Bruker D8 Venture and X8 APEX2 diffractometer (Bruker AXS GmbH, Karlsruhe, BRD) equipped with multilayer monochromators, Mo K/α INCOATEC micro focus sealed tubes and Oxford and Cryoflex2 cooling systems at 150 K (**1d**, **1e**) or 100 K (**7** tartrate). Crystal data are collected in Table 3. Experimental data and CCDC codes can be found in Supplementary Materials.

## Figures and Tables

**Table 1:** Asymmetric benzylation of *tert*-butyl 2-((diphenylmethylene)amino)acetate **12** with **13A** under PTC conditions yielding **14A**. ^a.^

entry	cat.	yield/% ^b^	yield/% ^c^	ee/% ^d^
1	**1a** ^e^	78	74	86
2	**1b** ^f^	91	89	94
3	**1c**	86	86	92
4	**1d**	92	88	91
5	**1e’**	72	69	rac.
6	**1f** ^g^	82	79	94
7	**1g** ^h^	90	85	99
8	**1h**	33	28	92
9	**1i**	67	67	88
10	**1j**	87	83	89
11	**5′**	92	91	61

^a^ 0.25 mmol substrate, 0.30 mmol benzylbromide, 1 mol % of catalyst with (*S,S*)-configuration, 1.5 mL toluene, 0.5 mL KOH (50%), vigorous stirring at 0 °C for 4 h. ^b^ Yield after extractive work-up based on ^1^H-NMR (nuclear magnetic resonance) integration of signals of product and methylene groups of bibenzyl as internal standard. ^c^ Isolated yield after extractive work-up and subsequent chromatography. ^d^ Determined by chiral high-performance liquid chromatography (HPLC, Chiralcel ODH), products with (*R*)-configuration predominating. ^e^ 89% ee, 81% isol. yield at 0 °C, 0.5 h [25,46]. ^f^ 96% ee, 95% isol.yield at 0 °C, 0.5 h [25]. ^g^ 94% ee, 74% isol. yield at 0 °C, 2 h [25]. ^h^ 99% ee, 79% isol. yield at 0 °C, 2 h [25].

**Table 2:** Asymmetric substitution of *tert*-butyl 2-((diphenylmethylene)amino)acetate (**12**) with electrophiles **13B-13I** under phase-transfer (PT) conditions. ^a.^

	**14B**	**14C**	**14D**	**14E**	**14F**	**14G**	**14H** ^b^	**14I** ^c^
**cat.**	y/*ee* ^d^	y/*ee* ^d^	y/*ee* ^d^	y/*ee* ^d^	y/*ee* ^d^	y/*ee* ^d^	y/*ee* ^d^	y/*ee* ^d^
**1c**	89/*88*	80/*91*	90/*75*	51/*83*	**92**/*89*	88/*72*	62/*51* ^e^	33/*57* ^f^
**1d**	**90**/*88*	**86**/*91*	90/*74*	55/***88***	91/*88*	**91**/*71*	56/*41* ^g^	26/*51* ^f^
**1g**	89/***98*** ^h^	71/***95*** ^i^	87/***93***	**72**/*68*	90/***90*** ^j^	81/***83***	**61**/*79*^k^	23/*83* ^f^ (**33**/***88****)*
**1h**	70/***98***	45/*52*	61/*84*	66/*14*	30/*90*	83/*73*	60/***80***	20/*83* ^i^

^a^ 0.25 mmol of substrate, 0.30 mmol of electrophile, 1 mol % of catalyst with (*S,S*)-configuration, 1.5 mL toluene, 0.5 mL KOH (50%), vigorous stirring at 0 °C for 20 h. ^b^ CsOH (50%) was used instead of KOH. ^c^ Solid Cs_2_CO_3_ (5 equiv.) was used instead of KOH. ^d^ Yield after extractive work-up based on ^1^H-NMR integration of signals of product and bibenzyl added as internal standard. Ee determined by chiral HPLC (Chiralcel ODH or Chiralpak ADH), products with (*R*)-configuration predominating. ^e^ 12% of **15** formed. ^f^ Instead of **13I** the corresponding iodo compound was used which gave higher yield and enantioselectivity with the excepion of cat. **1g** where the bromide was superior (in paranthesis). ^g^ 13% of **15** formed. ^h^ 99% ee, 80% isolated yield, 0 °C, 24 h [25]. ^i^ 99% ee 86% over two steps, 0 °C, 24 h [25]. ^j^ 99% ee 89% isolated yield, 0 °C, 15 h [25]. ^k^ 8% of **15** formed.

**Table 3:** Crystal data for **1d**, **1e**, and the less soluble (*S,S*)-dibenzoyltartrate of **7**.

	(*R*)(*R*)-1d	(*R*)_bina_(*S*)_biphe_-1e	(*R*)-7 (*S,S*)-dibenzoyltartrate
M [g/mol]	1323.48	791.92	811.22
Space group	*P212121*	*C2*	*C2*
a [Å]	9.1716(8)	21.7990(14)	28.7403(11)
b [Å]	26.018(2)	23.2291(13)	12.0555(4)
c [Å]	30.298(3)	8.8684(5)	8.9062(3)
α [°]	90	90	90
ß [°]	90	94.251(2)	101.095(2)
γ [°]	90	90	90
V [Å^3^]	7229.8(11)	4478.3(5)	3028.14(18)
Z	4	4	4
Dcalc [g/cm^3^]	1.216	1.175	1.779
R_int_	0.1022	0.0294	0.0359
R_sigma_	0.0399	0.0300	0.0175
R1 (I > 2σ(I))	0.0812	0.0802	0.0362
wR2 (all data)	0.2433	0.2295	0.0897

## Supplementary Materials

The following are available online: Comments on structure of side products upon rearrangements, ^1^H- and ^13^C-NMR spectra of all new compounds, comments on screening experiments, UV traces of chiral HPLC separations of racemates of **14A-I**, and details of X-ray structure determinations of **1d**, **1e**, and (*S,S*)-dibenzoyltartrate of **7**.
