# Supplementary material for: Improved Access to Chiral Tetranaphthoazepinium-Based Organocatalysts Using Aqueous Ammonia as Nitrogen Source
Source: Molecules. 2019 Oct 25;24(21):3844. doi: 10.3390/molecules24213844 (PMC6864439; doi:10.3390/molecules24213844)
Supplement: Supplementary file 1 [file molecules-24-03844-s001.zip › molecules-621572-supplementary materials.pdf]

Supplementary Materials

Improved Access to Chiral Tetranaphthoazepinium-Based Organocatalysts Using Aqueous Ammonia as Nitrogen Source

Auraya Manaprasertsak, Sorachat Tharamak, Christina Schedl, Alexander Roller and Michael Widhalm

Content

Proposed structures of sequence products originating from a Stevens rearrangement of **1e** and **5** 2

^{1}H NMR and ^{13}C NMR spectra 10

Comments on screening experiments in PTC – Practical aspects 68

UV traces of chiral HPLC separations 71

Crystal structure analysis of **1d**, **1e**, and of the less soluble tartrate of **7** 80

Proposed sequence products of a Stevens rearrangement of **1e** and **5**, respectively

During attempted spirocyclisation of (*S*)-**9e** with (*S*)-2,2'-bisbromomethyl-1,1'-binaphthyl in MeCN the target compound (*S*)(*S*)-**1e** was isolated in small amounts (<10%, ~80% purity). Instead considerable amounts of dinaphthooxepine along with a sequence product **1e-S** from **1e** resulting from Stevens rearrangement was detected. Feasible structures *A-D* are depicted in Scheme S1. Based on the ^{1}H NMR spectrum showing seven aliphatic protons in different environment (2x AB, 1x ABX system) structure *C* and *D* can be excluded. From steric reasons an equatorial attack of the CH_2 group will be favored resulting in an axial methine proton in *A* or *B*. Due to the complexity of NMR spectra in the aromatic range only partial assignment of signals was done and no decision between structure *A* and *B* for **1e-S** is possible at the time.

Scheme S1. Stevens Rearrangement of Postulated Dinaphthoazepinium Bromides **1e** and **5** with Bulky Substituents.

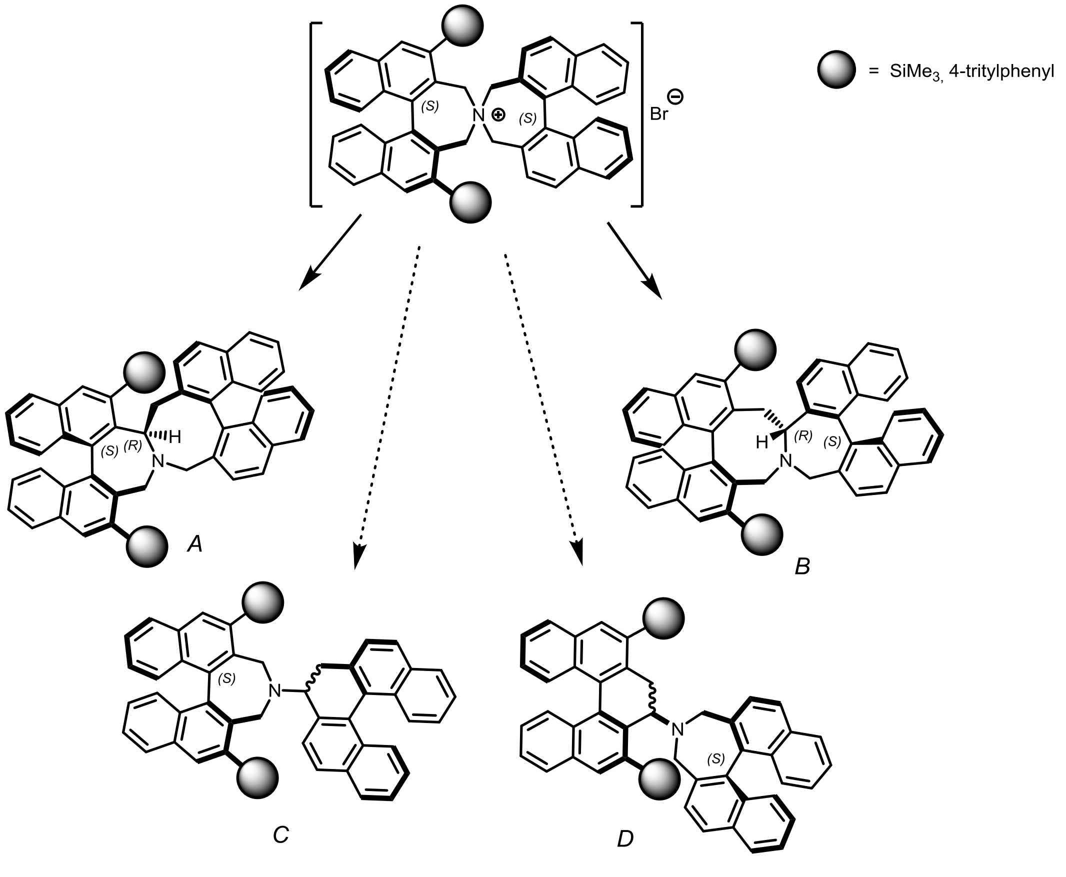

Also in case of (*S*)-**5** reaction of **4** with (*S*)-2,2'-bisbromomethyl-1,1'-binaphthyl afforded a rearranged product **5-S** with similar ^{1}H NMR spectrum and correct HRMS. It is worth to note that the same product **5-S** was also obtained from unsubstituted dinaphthoazepine and dibromide **3**, indicative for running

through a common intermediate **5** (Scheme S2). Attempted transformation into corresponding methylammonium salts failed.

Scheme S2. Access of **5-S** from different precursors.

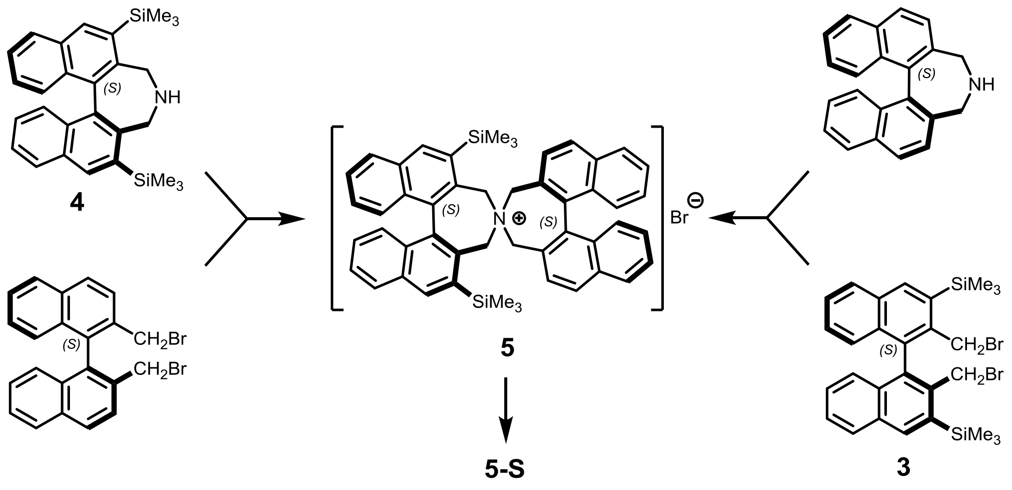

1e-S: HRMS: Calcd for $C_{94}H_{68}N$ $[M + H]^+$: 1210.5347, found: 1210.5337.

5-S: HRMS: Calcd for $C_{50}H_{47}NSi_2$ $[M + H]^+$: 718.3325, found: 716.3314.

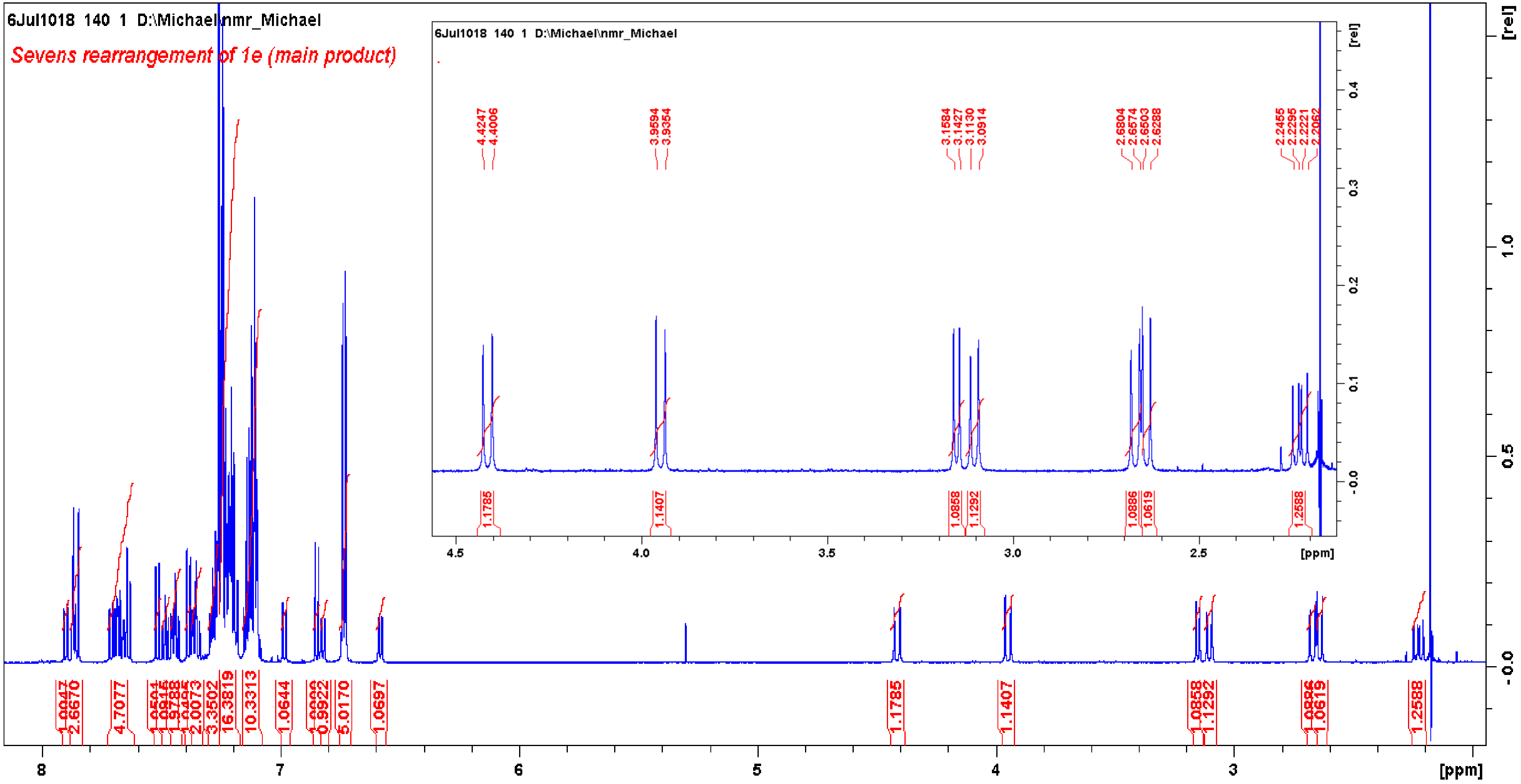

6Jul1018 140 1 D:\Michael\nmr_Michael
Sevens rearrangement of 1e (main product)
[ppm]
[rel]

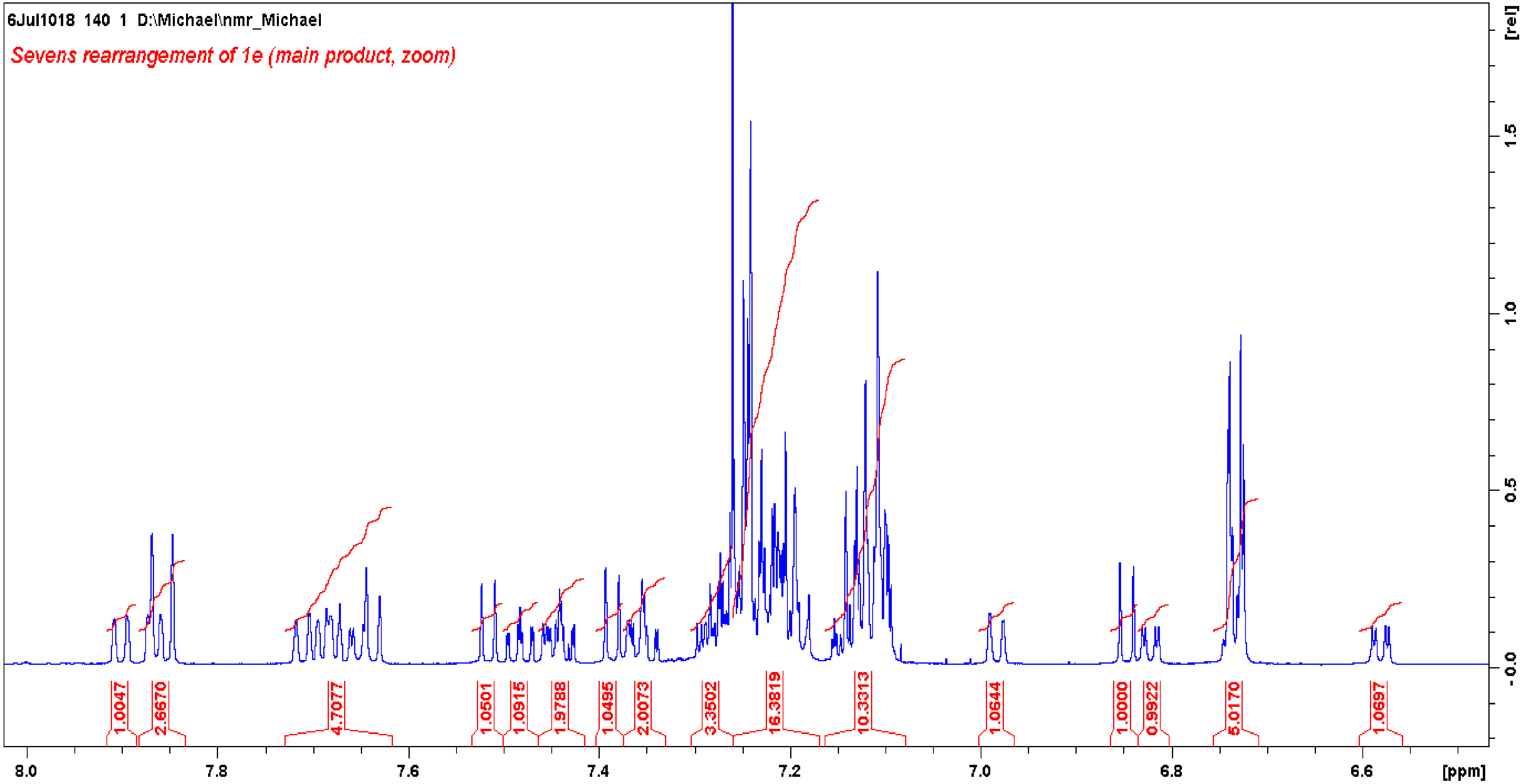
6Jul1018 140 1 D:\Michael\nmr_Michael
Sevens rearrangement of 1e (main product, zoom)
1.0047
2.6670
4.7077
1.0501
1.0915
1.9788
1.0495
2.0073
3.3502
16.3819
10.3313
1.0644
1.0000
0.9922
5.0170
1.0697
8.0
7.8
7.6
7.4
7.2
7.0
6.8
6.6
[ppm]
[rel]
1.5
1.0
0.5
-0.0

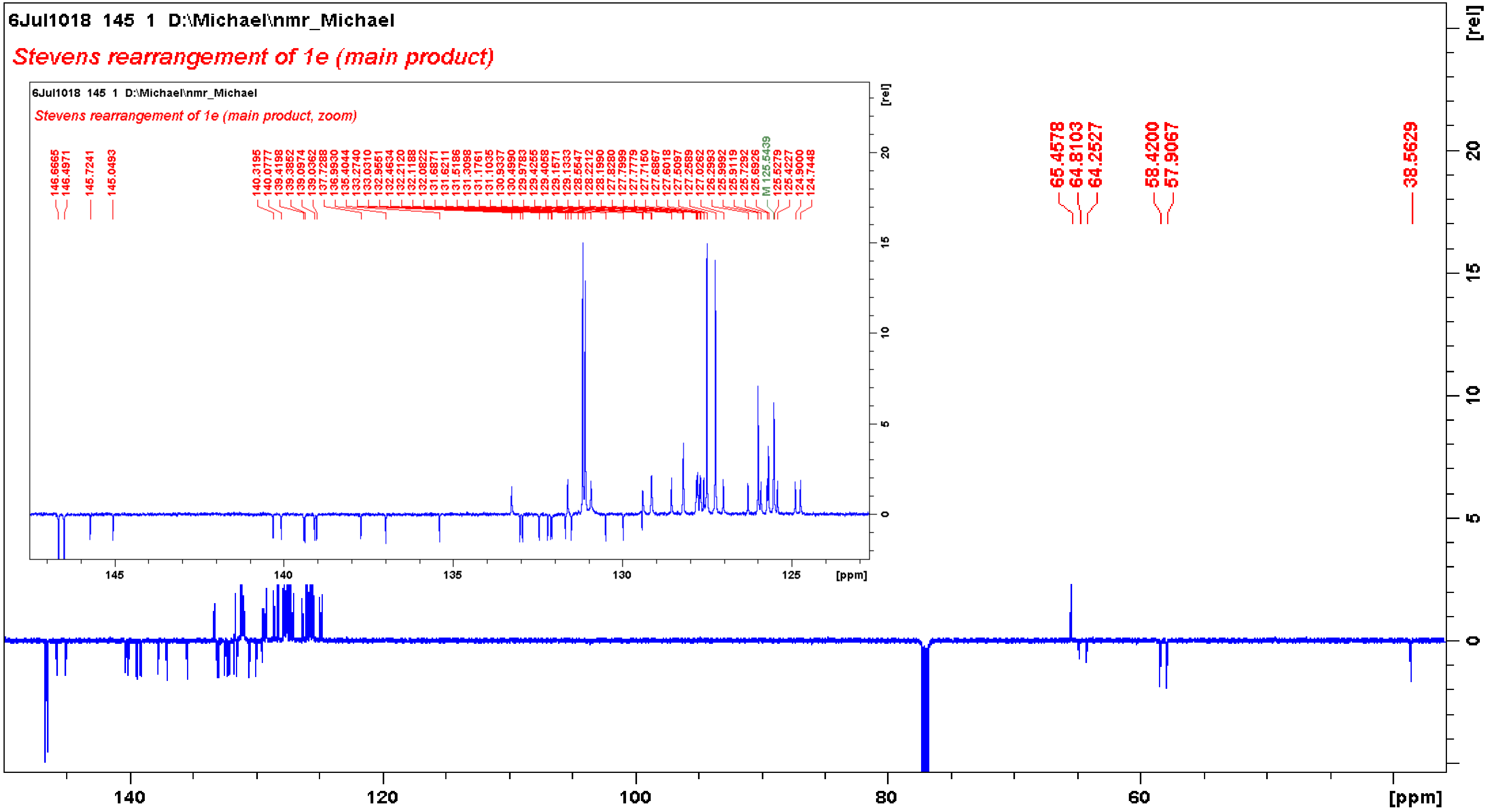
6Jul1018 145 1 D:\Michael\nmr_Michael
Stevens rearrangement of 1e (main product)
6Jul1018 145 1 D:\Michael\nmr_Michael
Stevens rearrangement of 1e (main product, zoom)
65.4578
64.8103
64.2527
58.4200
57.9067
38.5629
[ppm]
[rel]

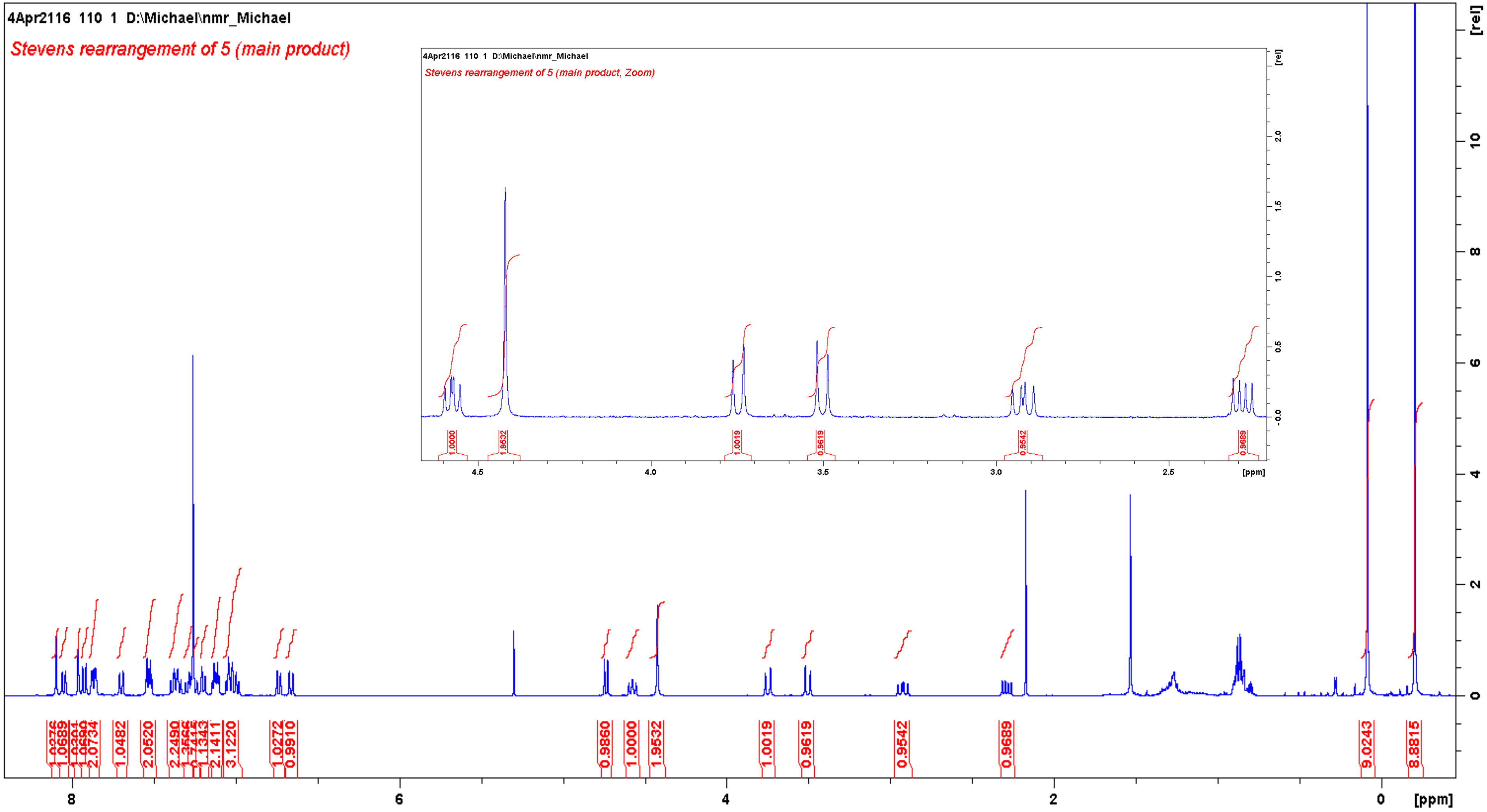
4Apr2116 110 1 D:\Michael\nmr_Michael
Stevens rearrangement of 5 (main product)
4Apr2116 110 1 D:\Michael\nmr_Michael
Stevens rearrangement of 5 (main product, Zoom)
[rel]
[ppm]

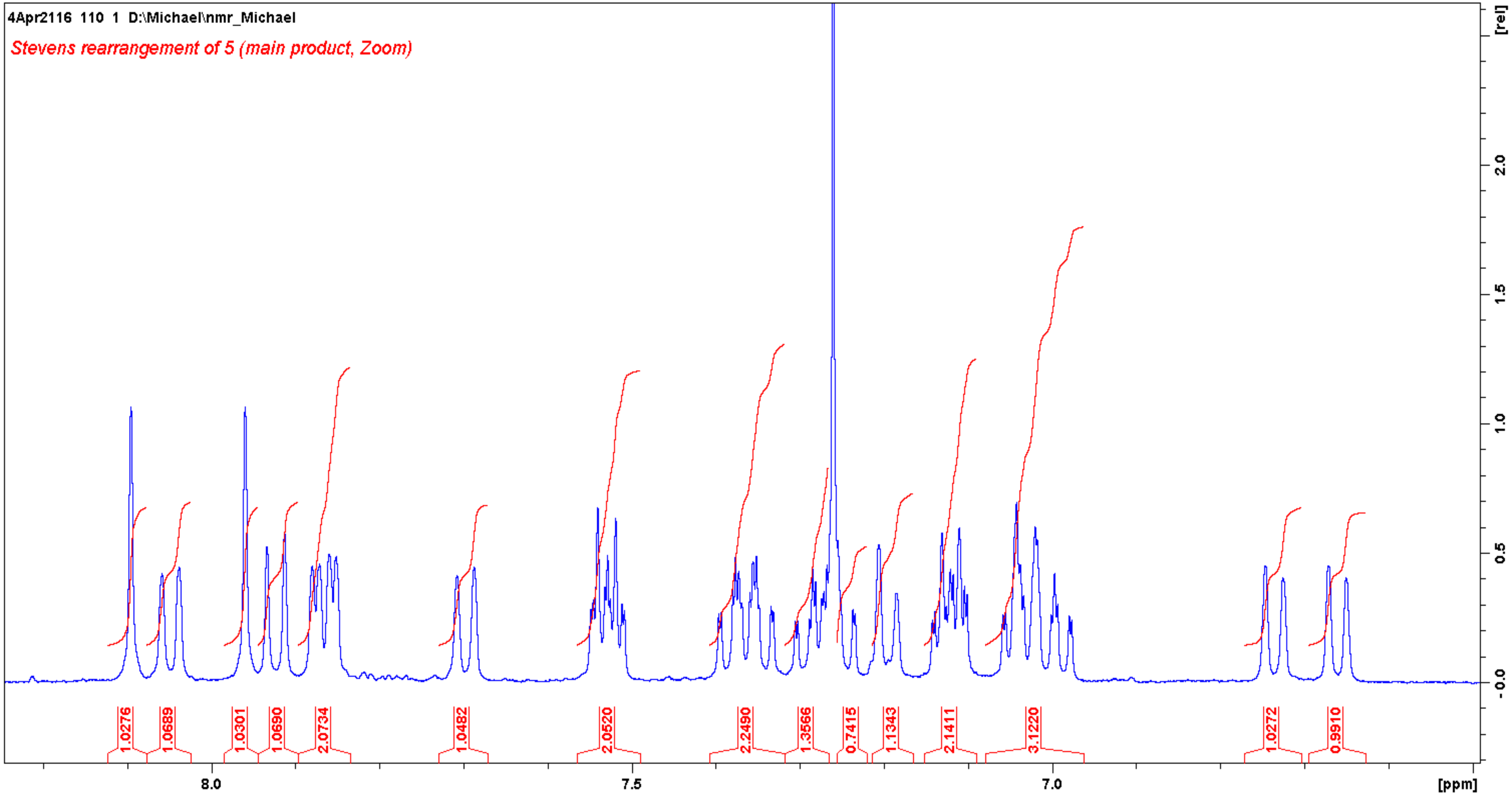
4Apr2116 110 1 D:\Michael\nmr_Michael
Stevens rearrangement of 5 (main product, Zoom)
1.0276
1.0689
1.0301
1.0690
2.0734
1.0482
2.0520
2.2490
1.3566
0.7415
1.1343
2.1411
3.1220
1.0272
0.9910
8.0
7.5
7.0
[ppm]
[rel]
2.0
1.5
1.0
0.5
0.0

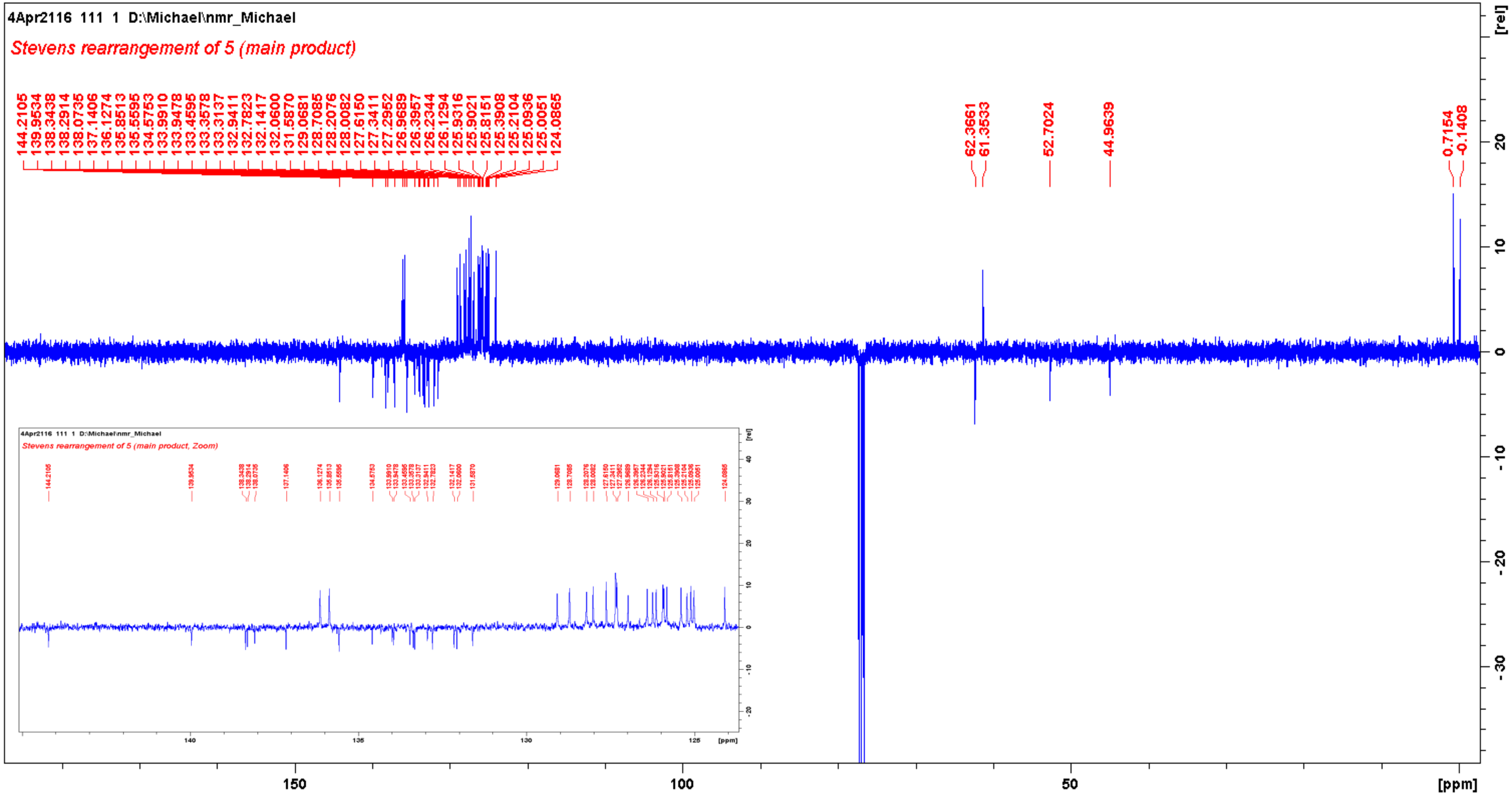
4Apr2116 111 1 D:\Michael\nmr_Michael
Stevens rearrangement of 5 (main product)
144.2105
139.9534
138.3438
138.2914
138.0735
137.1406
136.1274
135.8513
135.5595
134.5753
133.9910
133.9478
133.4595
133.3578
133.3137
132.9411
132.7823
132.1417
132.0600
131.5870
129.0681
128.7085
128.2076
128.0082
127.6150
127.3411
127.2952
126.9689
126.3957
126.2344
126.1294
125.9316
125.9021
125.8151
125.3908
125.2104
125.0936
125.0051
124.0865
62.3661
61.3533
52.7024
44.9639
0.7154
-0.1408
[rel]
[ppm]
Stevens rearrangement of 5 (main product, Zoom)

<u>^{1}H- and ^{13}C NMR spectra</u> (If not otherwise noted spectra are recorded at room temperature in $CDCl_3$)

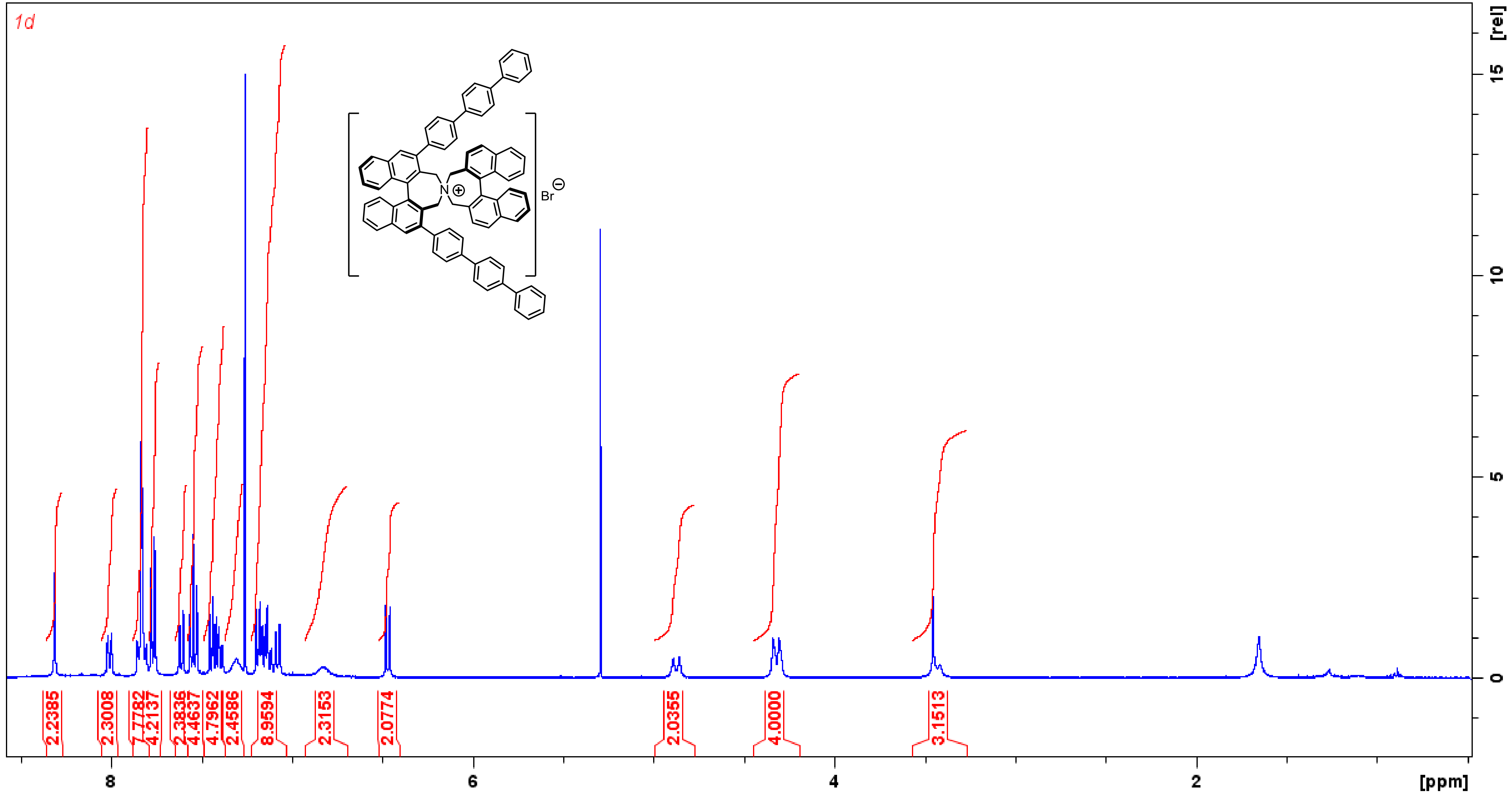

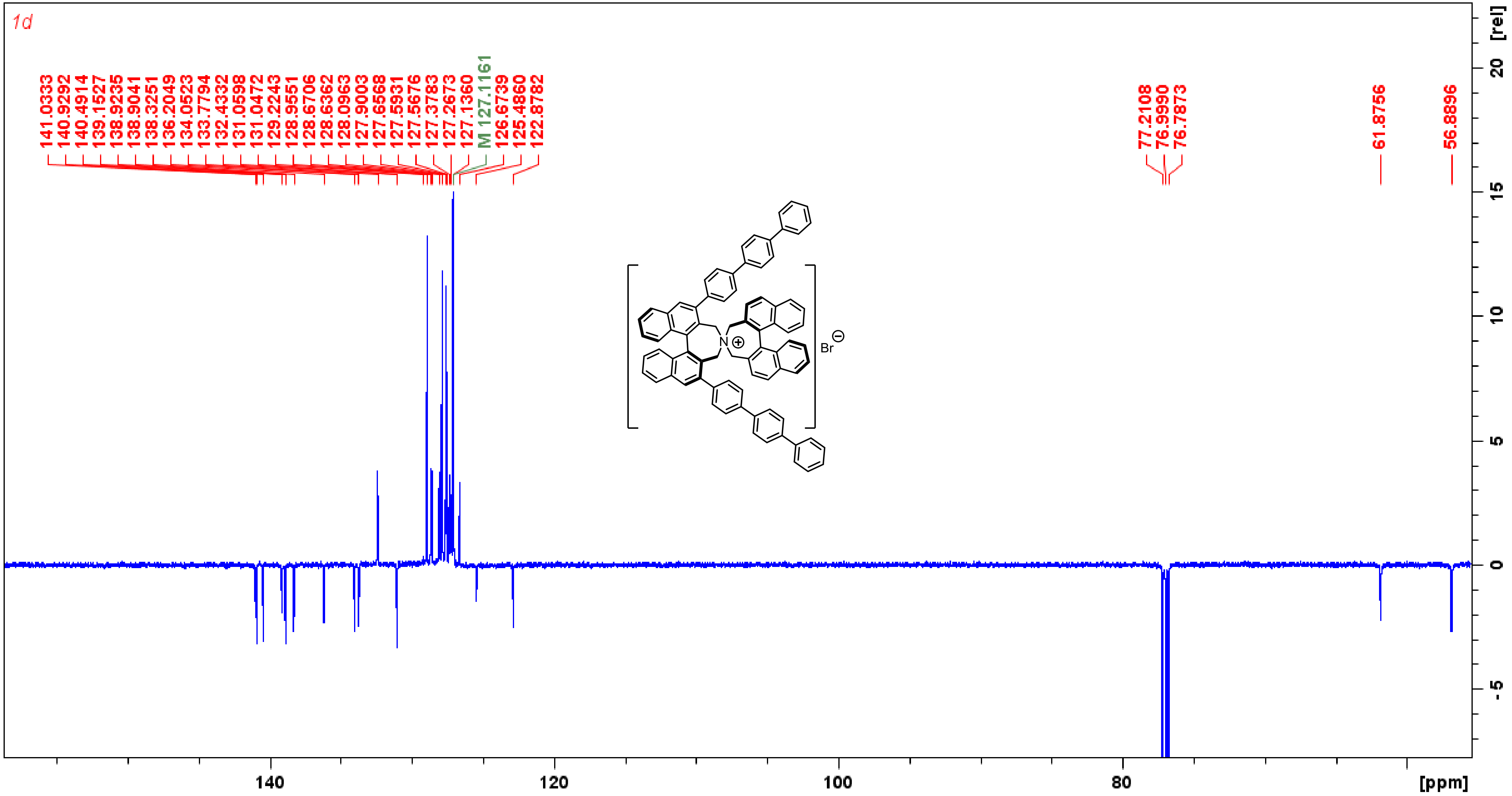
1d
141.0333
140.9292
140.4914
139.1527
138.9235
138.9041
138.3251
136.2049
134.0523
133.7794
132.4332
131.0598
131.0472
129.2243
128.9551
128.6706
128.6362
128.0963
127.9003
127.6568
127.5931
127.5676
127.3783
127.2673
127.1360
M 127.1161
126.6739
125.4860
122.8782
77.2108
76.9990
76.7873
61.8756
56.8896
N
Br
[ppm]
[rel]

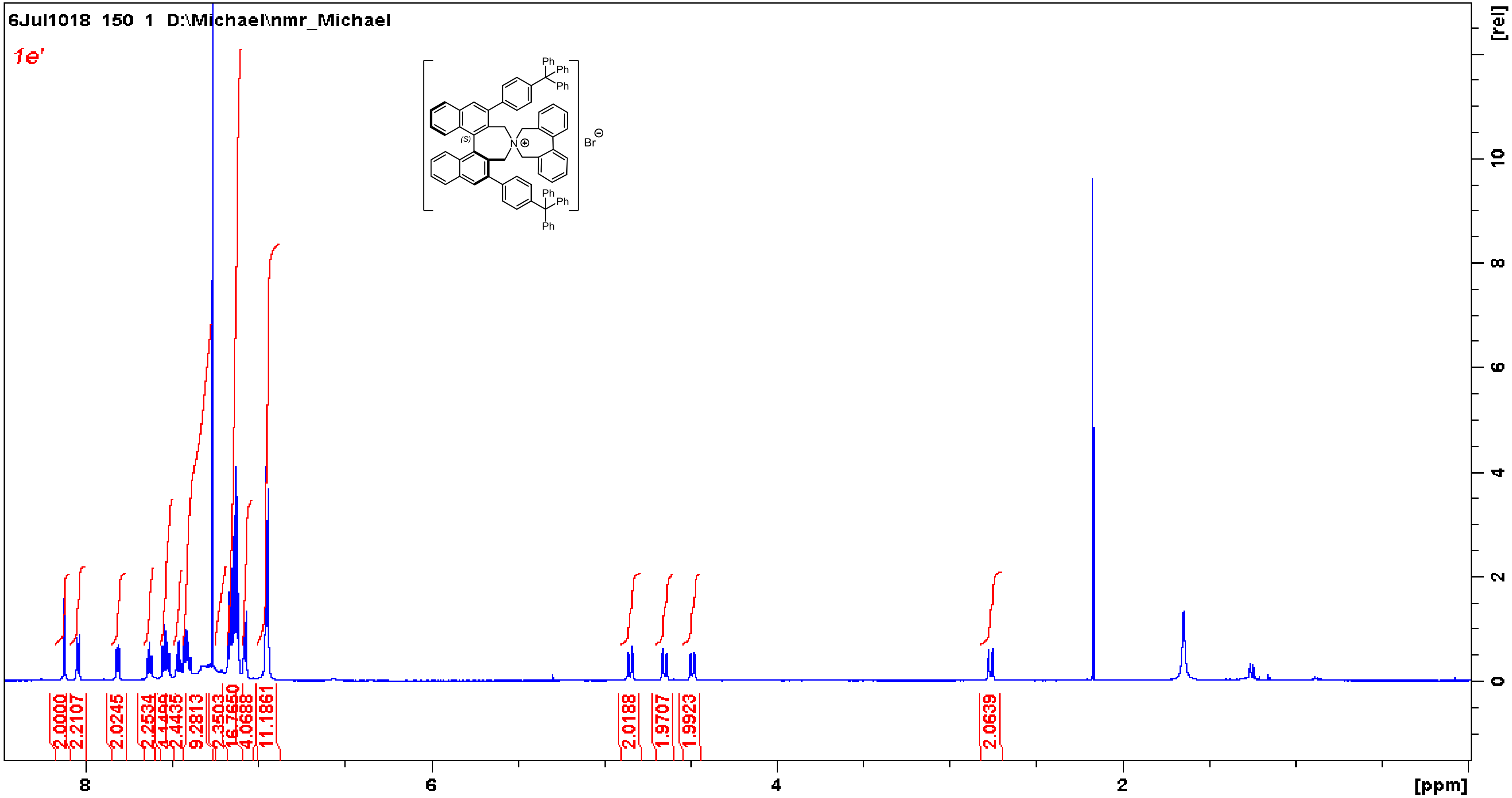

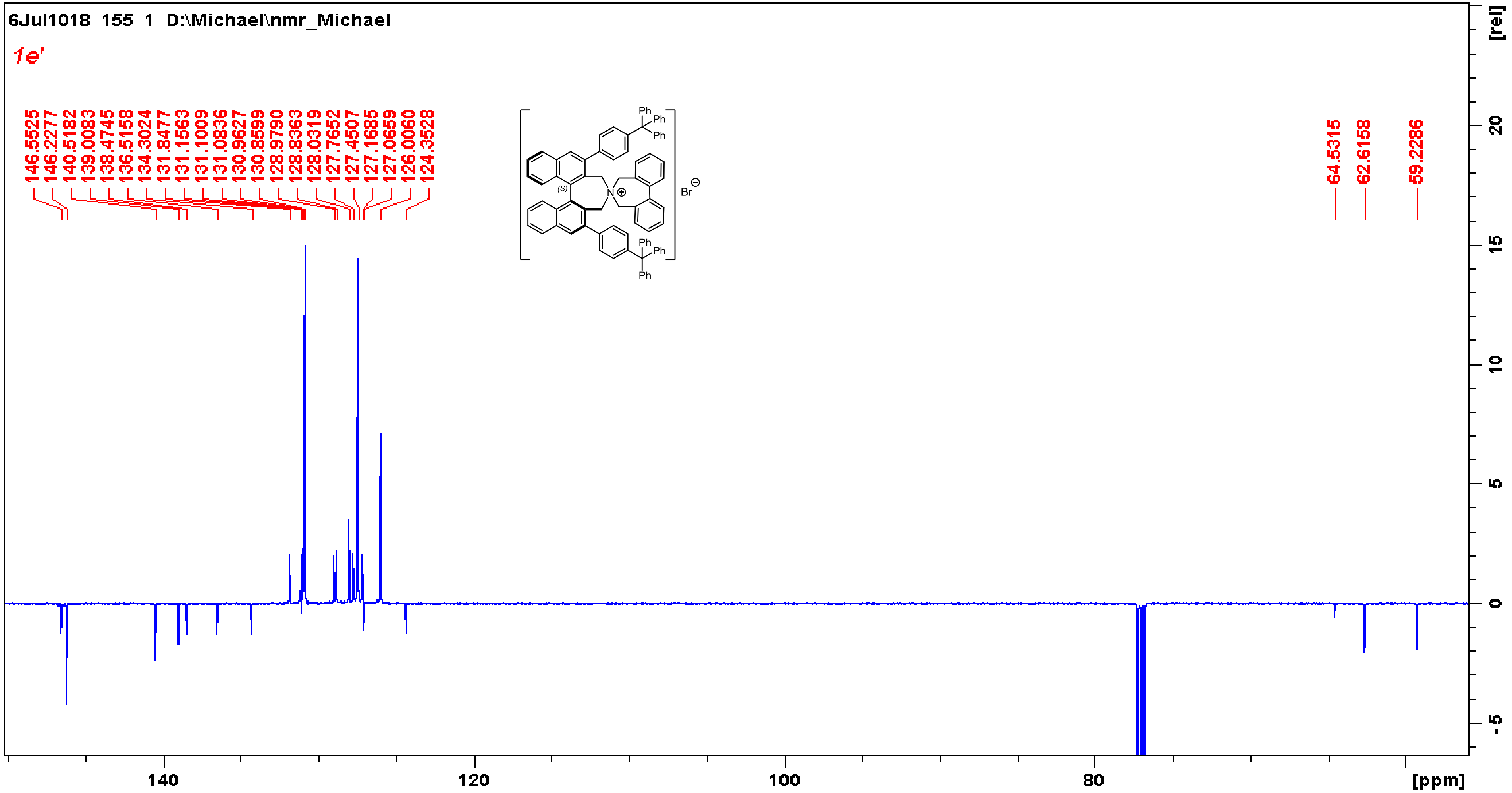
6Jul1018 155 1 D:\Michael\nmr_Michael
1e'
146.5525
146.2277
140.5182
139.0083
138.4745
136.5158
134.3024
131.8477
131.1563
131.1009
131.0836
130.9627
130.8599
128.9790
128.8363
128.0319
127.7652
127.4507
127.1685
127.0659
126.0060
124.3528
64.5315
62.6158
59.2286
Ph
Ph
Ph
(S)
N⊕
Br⊖
[rel]
[ppm]
140
120
100
80
20
15
10
5
0
-5

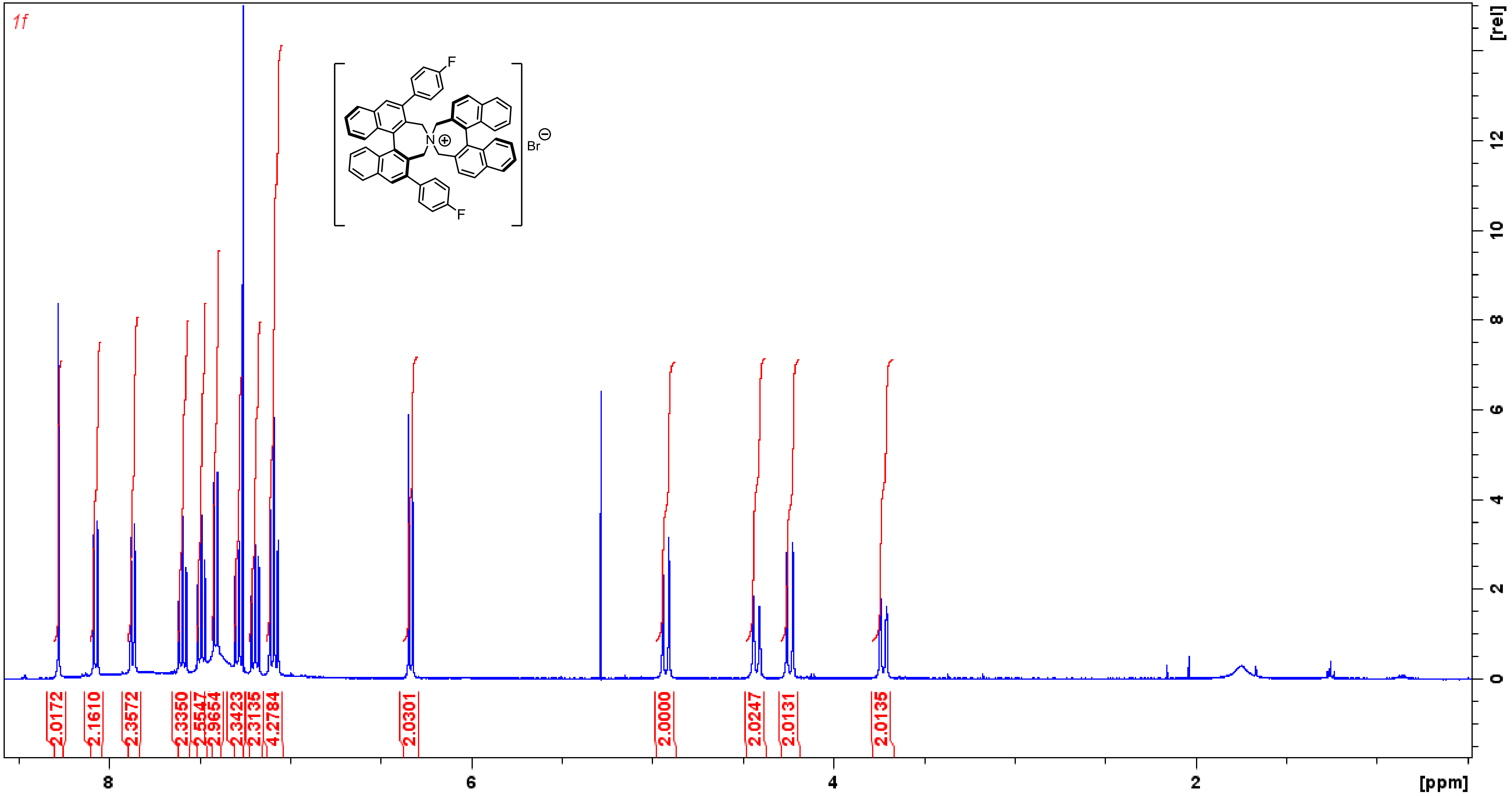
1f
F
F
N
Br
2.0172
2.1610
2.3572
2.3350
2.5547
2.9654
2.3423
2.3135
4.2784
2.0301
2.0000
2.0247
2.0131
2.0135
8
6
4
2
[ppm]
[rel]
12
10
8
6
4
2
0

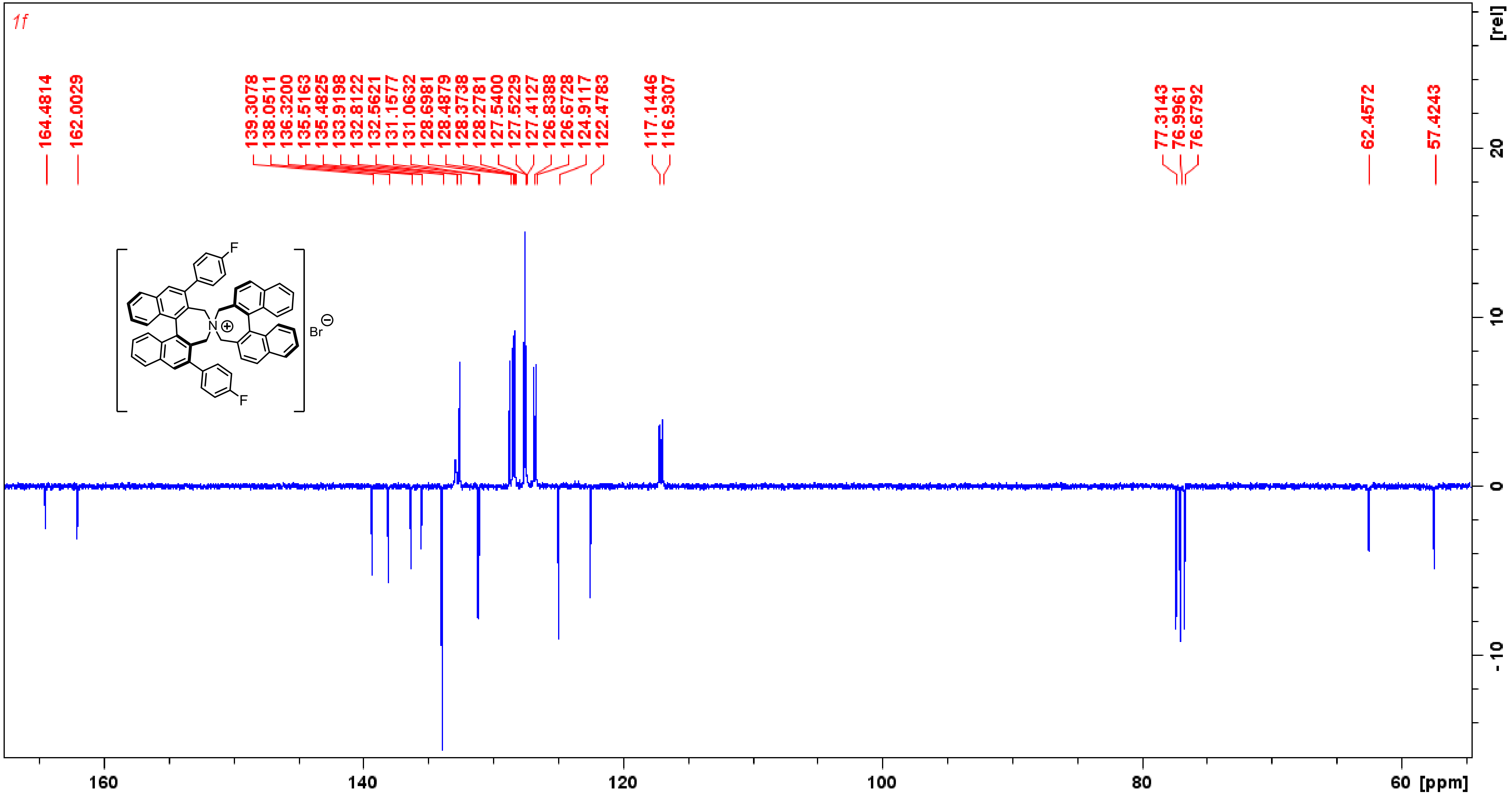
1f
164.4814
162.0029
139.3078
138.0511
136.3200
135.5163
135.4825
133.9198
132.8122
132.5621
131.1577
131.0632
128.6981
128.4879
128.3738
128.2781
127.5400
127.5229
127.4127
126.8388
126.6728
124.9117
122.4783
117.1446
116.9307
77.3143
76.9961
76.6792
62.4572
57.4243
F
N
Br
[rel]
20
10
0
-10
160
140
120
100
80
60 [ppm]

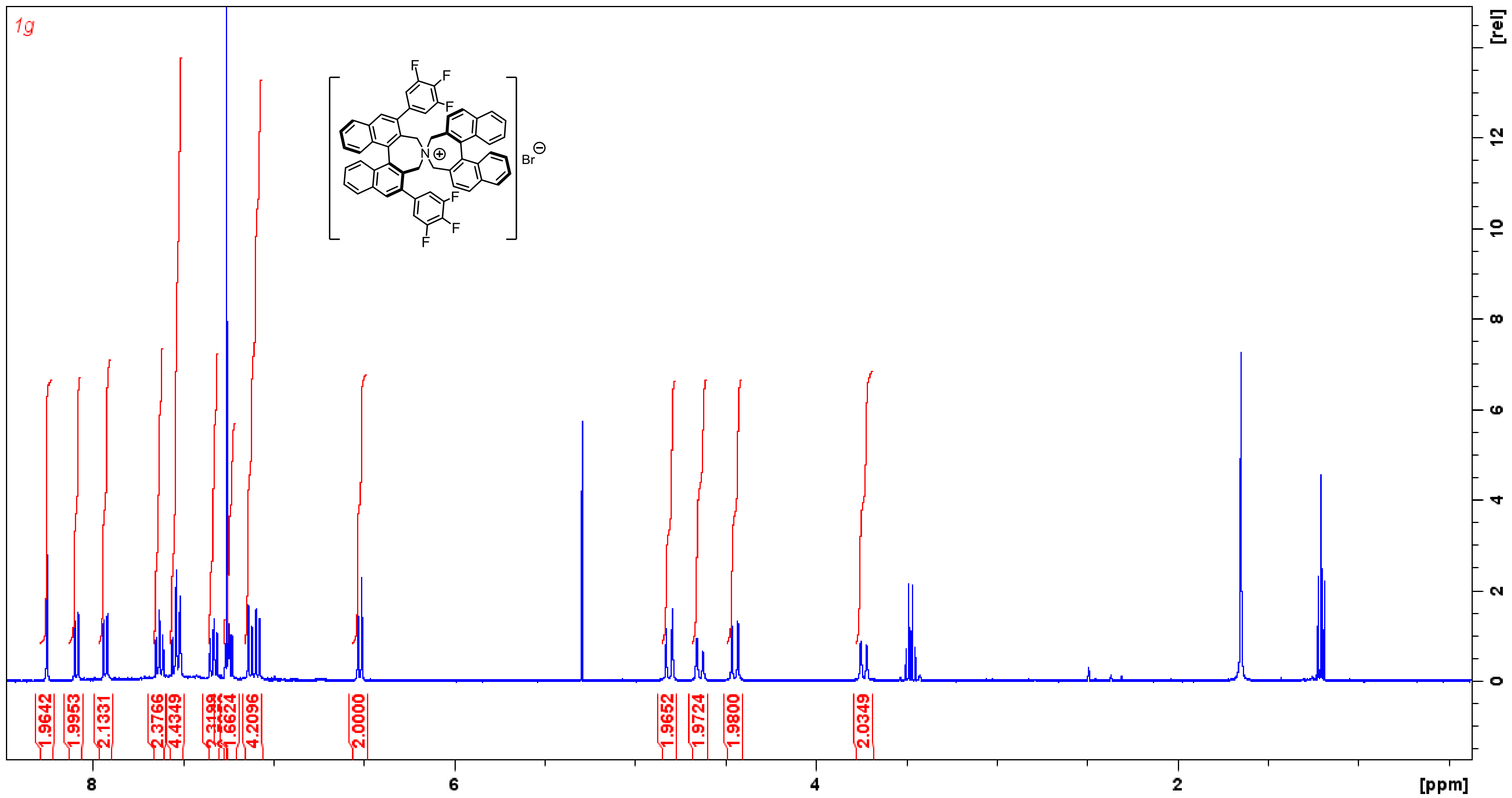
1g
Br
[rel]
[ppm]
1.9642
1.9953
2.1331
2.3766
4.4349
1.6624
4.2096
2.0000
1.9652
1.9724
1.9800
2.0349

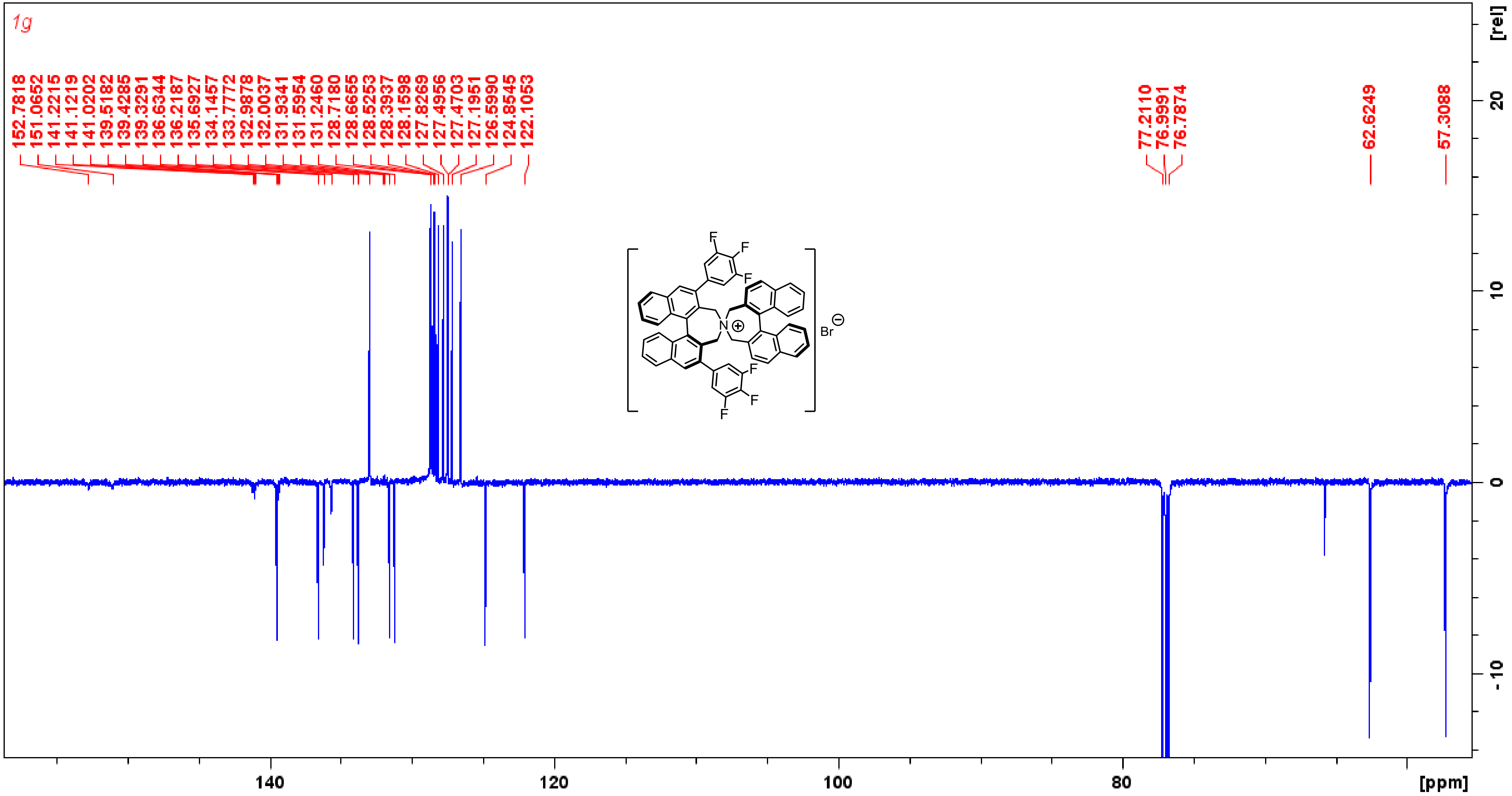
1g
152.7818
151.0652
141.2215
141.1219
141.0202
139.5182
139.4285
139.3291
136.6344
136.2187
135.6927
134.1457
133.7772
132.9878
132.0037
131.9341
131.5954
131.2460
128.7180
128.6655
128.5253
128.3937
128.1598
127.8269
127.4956
127.4703
127.1951
126.5990
124.8545
122.1053
77.2110
76.9991
76.7874
62.6249
57.3088
F
F
F
F
F
F
N
Br
[rel]
20
10
0
-10
140
120
100
80
[ppm]

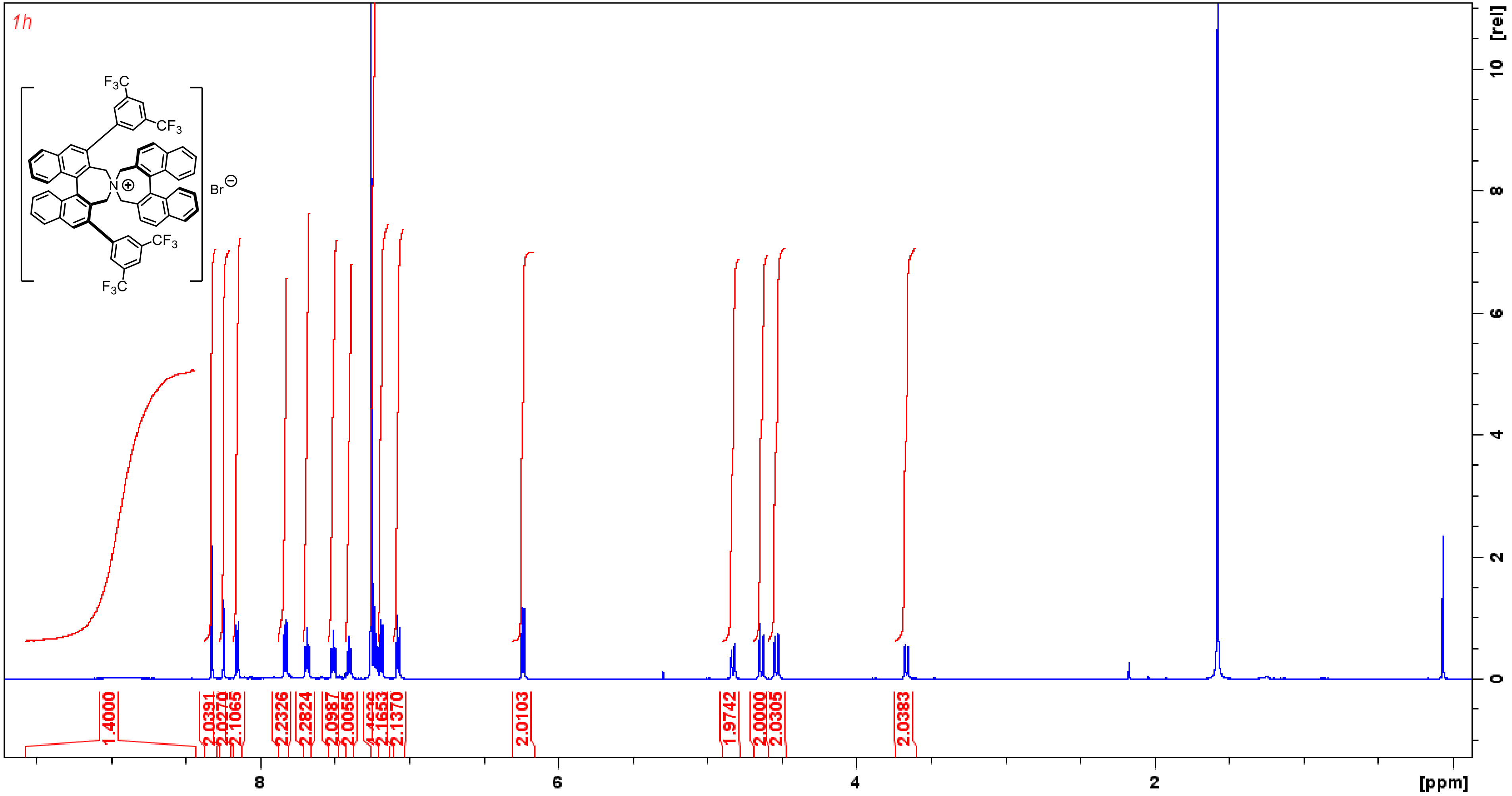

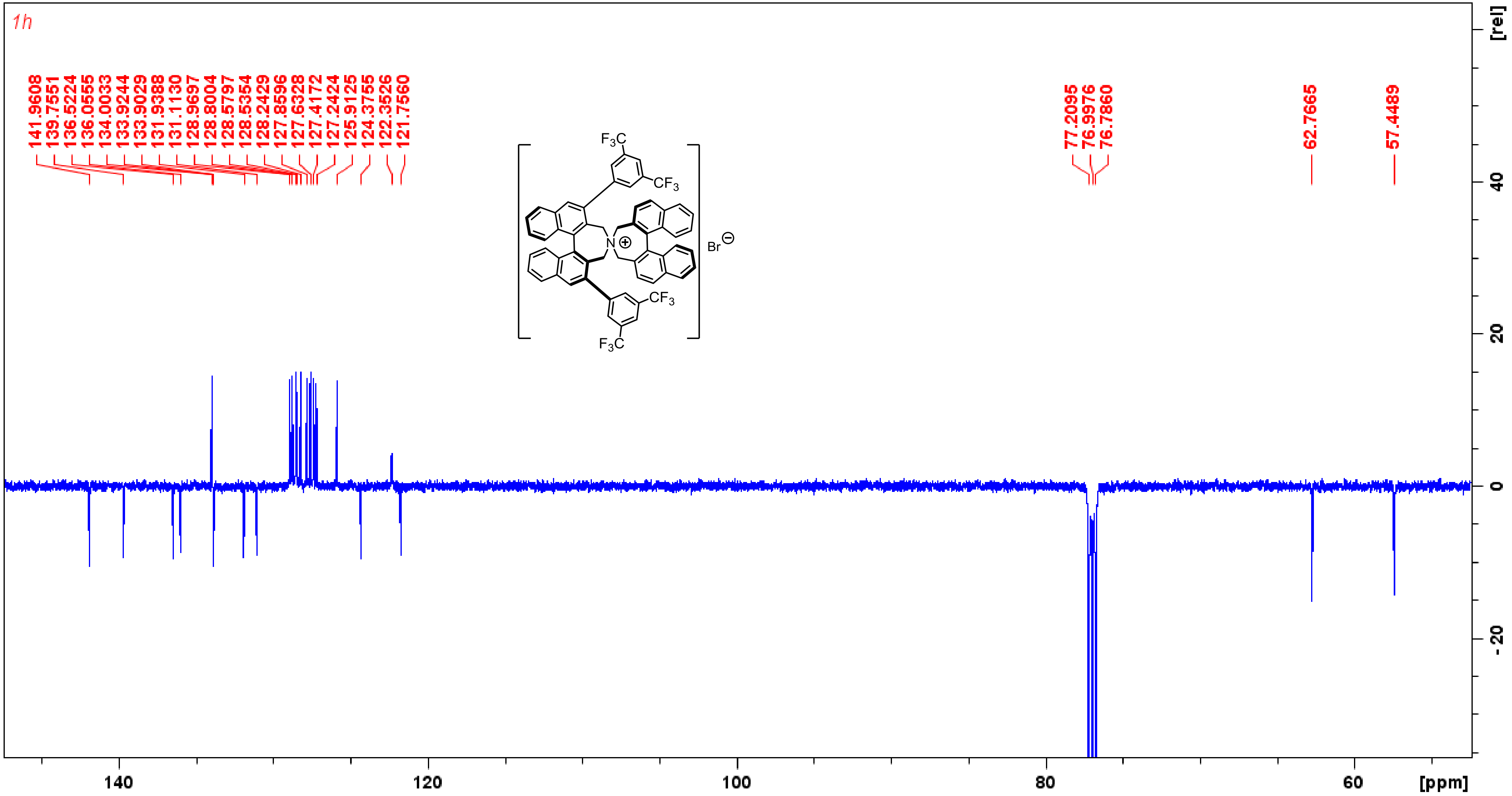
1h
141.9608
139.7551
136.5224
136.0555
134.0033
133.9244
133.9029
131.9388
131.1130
128.9697
128.8004
128.5797
128.5354
128.2429
127.8596
127.6328
127.4172
127.2424
125.9125
124.3755
122.3526
121.7560
77.2095
76.9976
76.7860
62.7665
57.4489
F3C
CF3
Br
[rel]
140
120
100
80
60
[ppm]

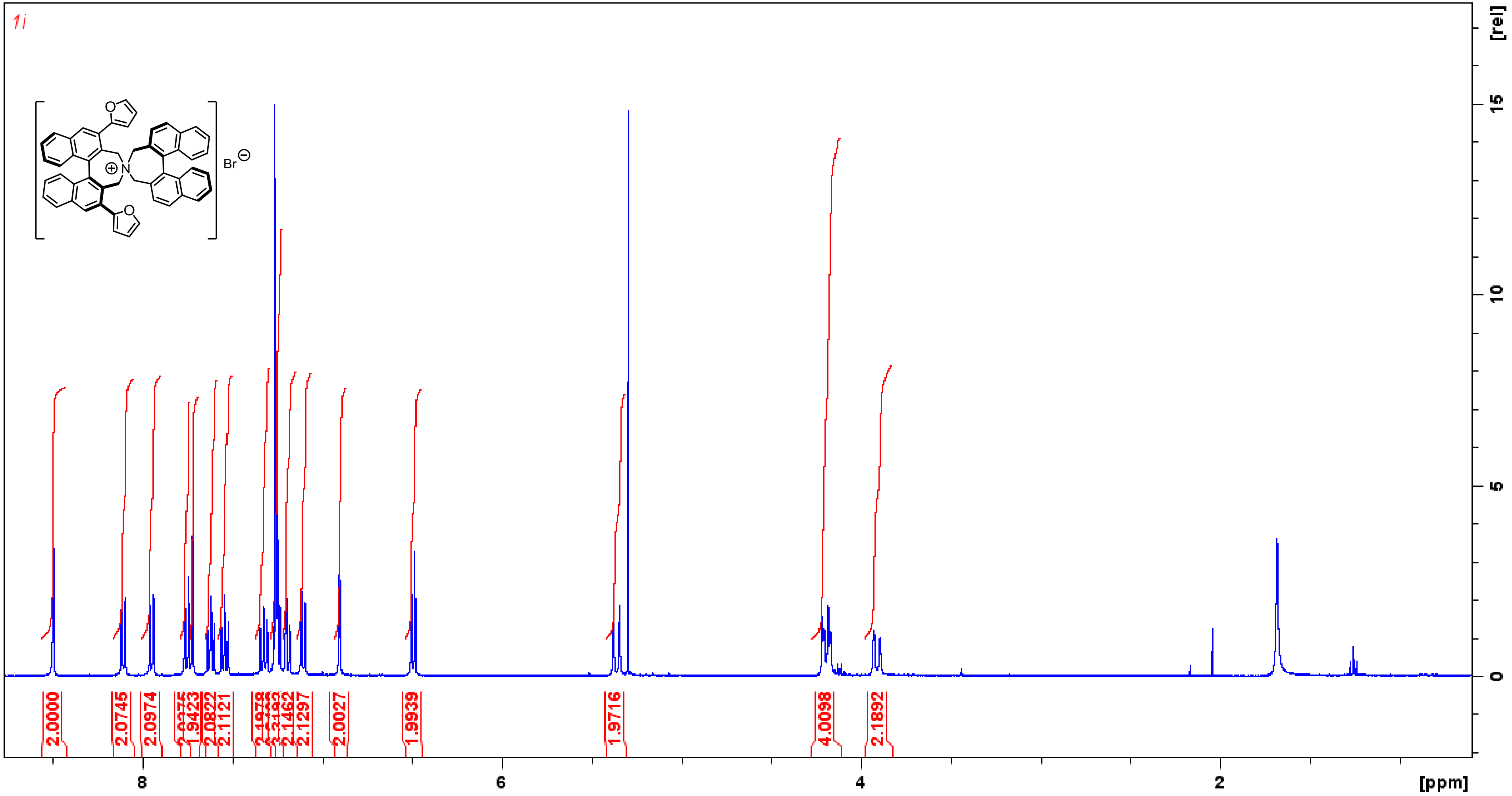
1i
Br
[rel]
15
10
5
0
2.0000
2.0745
2.0974
1.9423
2.0822
2.1121
2.1462
2.1297
2.0027
1.9939
1.9716
4.0098
2.1892
8
6
4
2
[ppm]

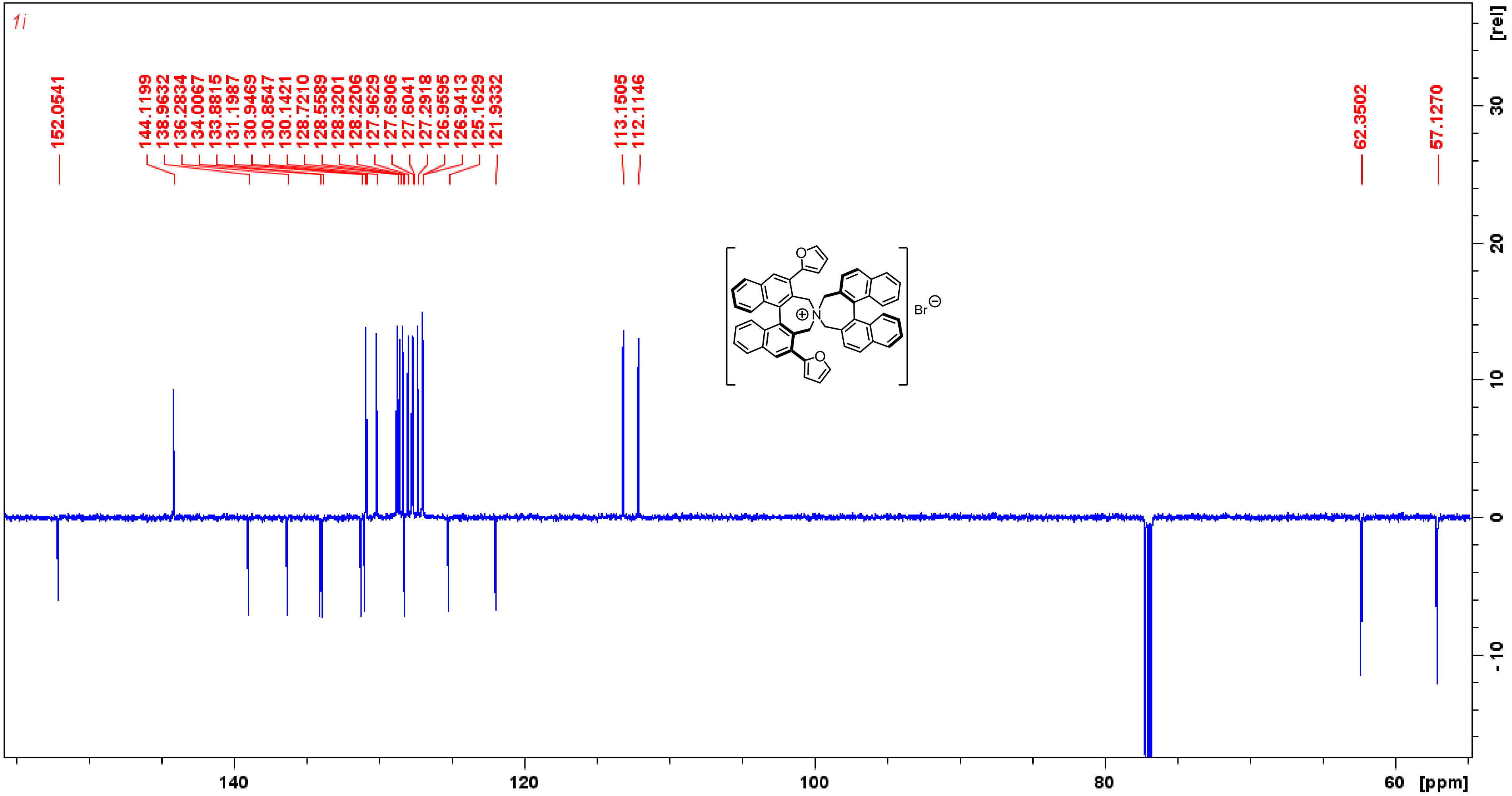
1i
152.0541
144.1199
138.9632
136.2834
134.0067
133.8815
131.1987
130.9469
130.8547
130.1421
128.7210
128.5589
128.3201
128.2206
127.9629
127.6906
127.6041
127.2918
126.9595
126.9413
125.1629
121.9332
113.1505
112.1146
62.3502
57.1270
Br
140
120
100
80
60
[ppm]
[rel]
30
20
10
0
-10

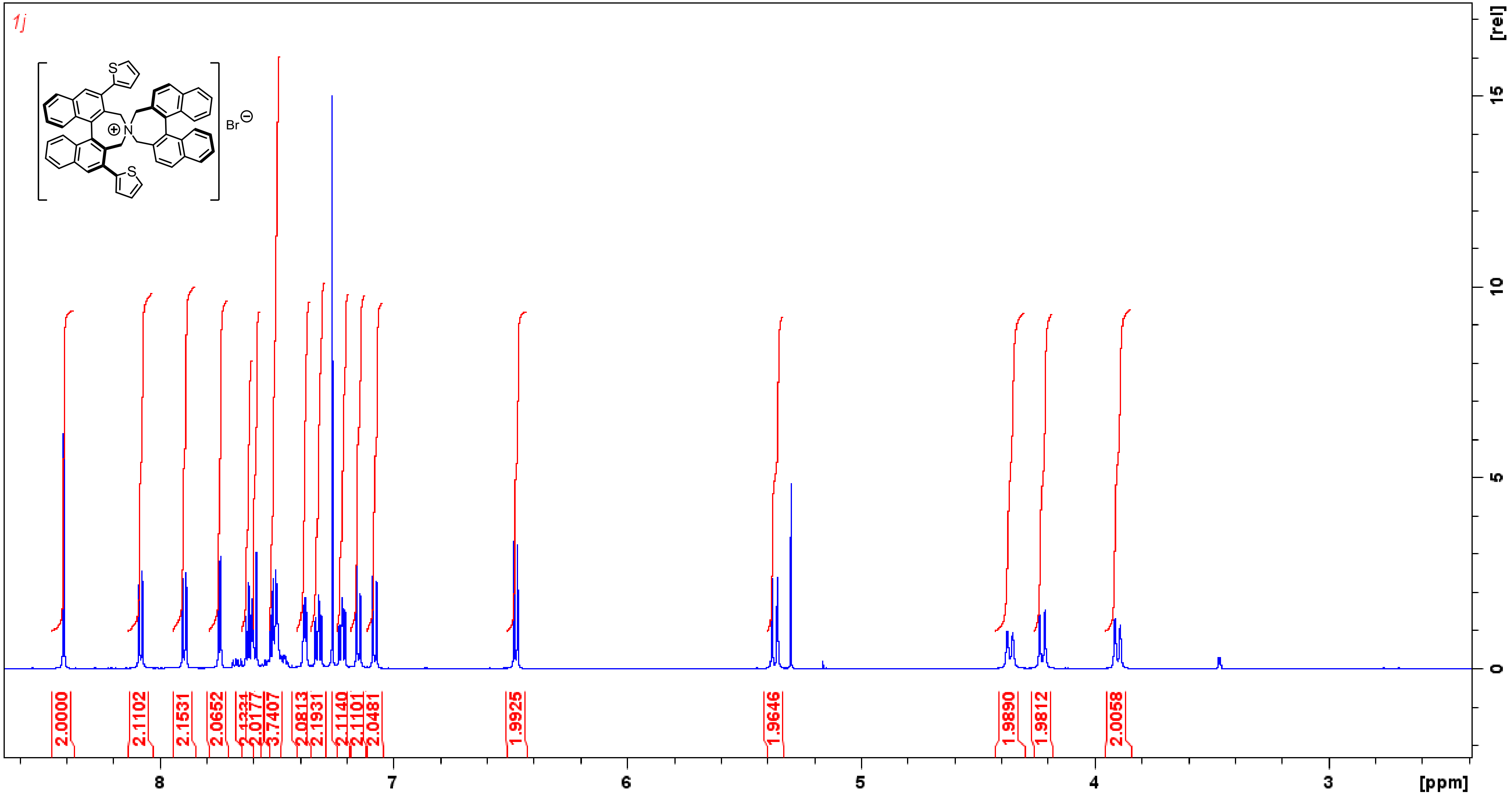
1j
Br
2.0000
2.1102
2.1531
2.0652
2.1324
2.0177
3.7407
2.0813
2.1931
2.1140
2.1101
2.0481
1.9925
1.9646
1.9890
1.9812
2.0058
8
7
6
5
4
3
[ppm]
0
5
10
15
[rel]

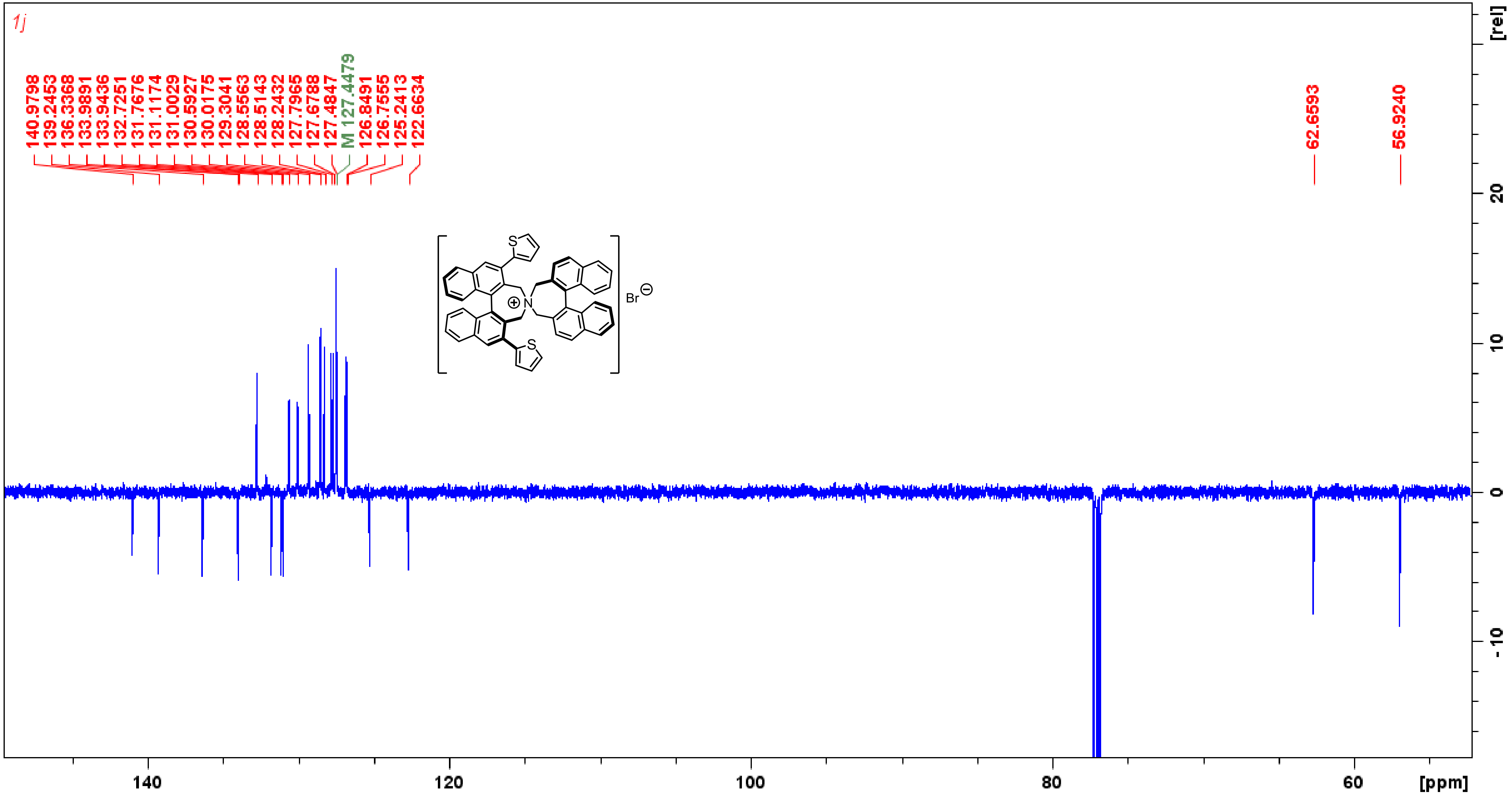
1j
140.9798
139.2453
136.3368
133.9891
133.9436
132.7251
131.7676
131.1174
131.0029
130.5927
130.0175
129.3041
128.5563
128.5143
128.2432
127.7965
127.6788
127.4847
M 127.4479
126.8491
126.7555
125.2413
122.6634
62.6593
56.9240
Br
N
S
[rel]
20
10
0
-10
140
120
100
80
60
[ppm]

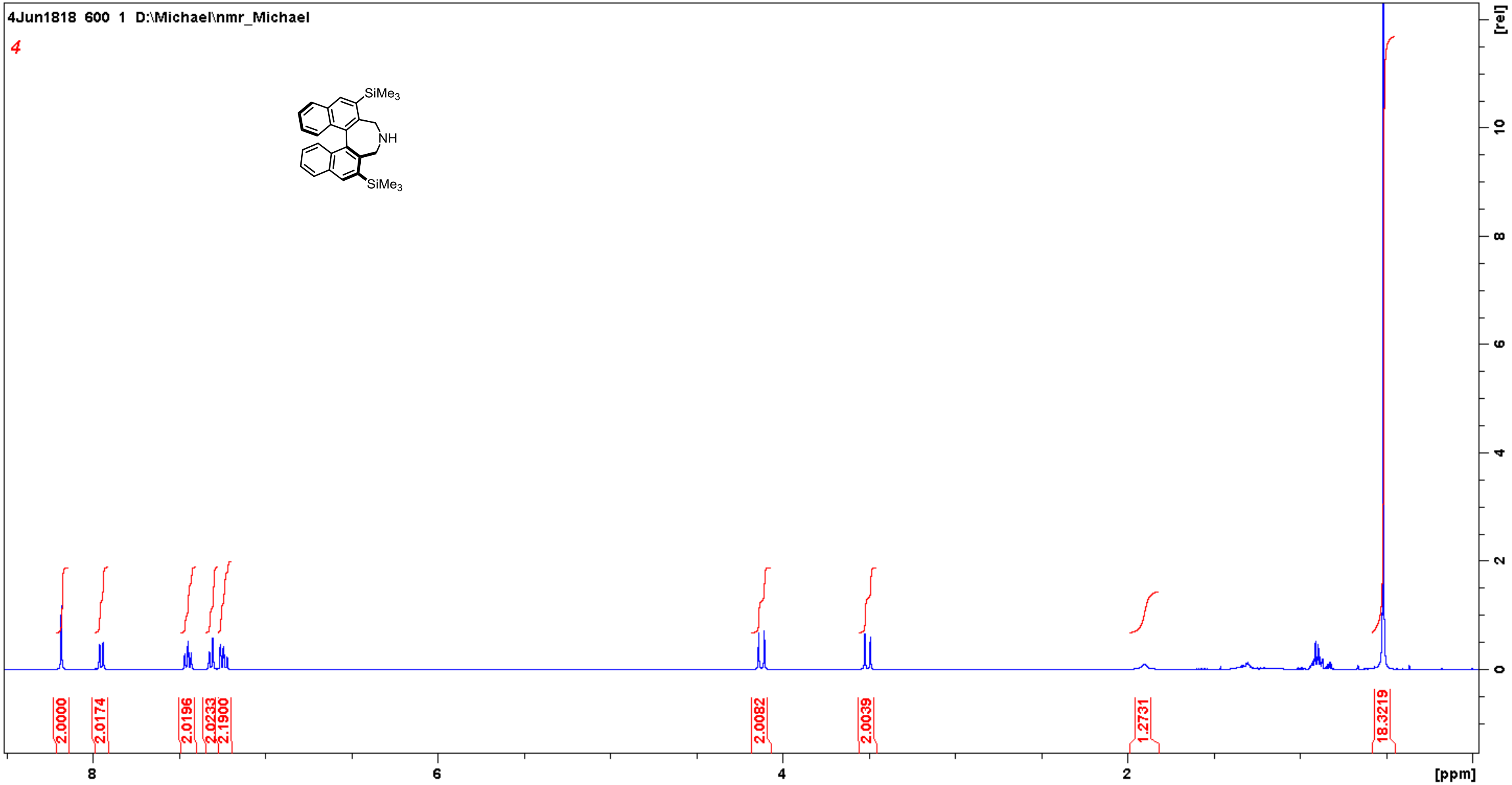
4Jun1818 600 1 D:\Michael\nmr_Michael
4
SiMe3
NH
SiMe3
2.0000
2.0174
2.0196
2.0233
2.1900
2.0082
2.0039
1.2731
18.3219
8
6
4
2
[ppm]
[rel]

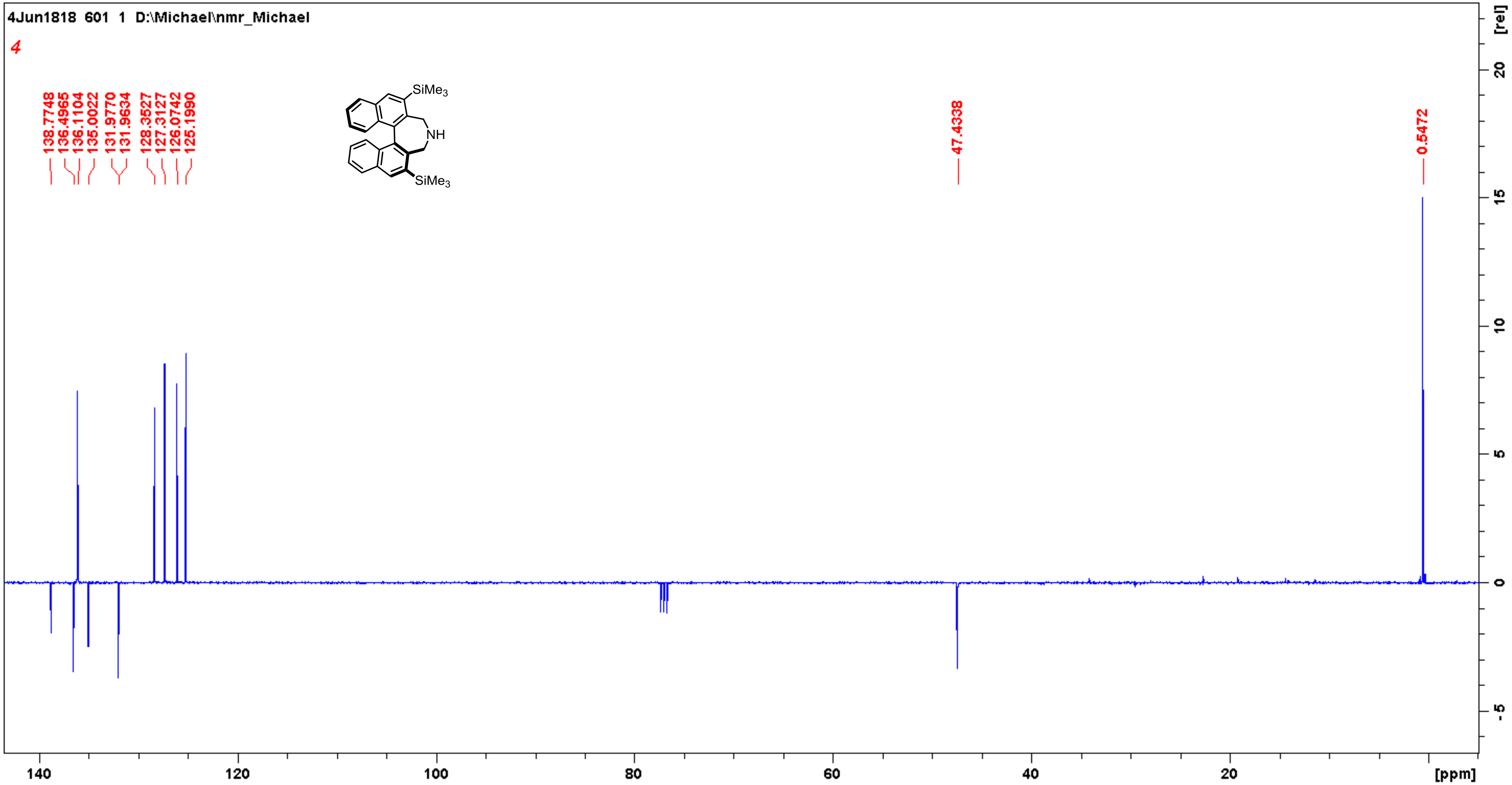
4Jun1818 601 1 D:\Michael\nmr_Michael
4
138.7748
136.4965
136.1104
135.0022
131.9770
131.9634
128.3527
127.3127
126.0742
125.1990
SiMe3
NH
SiMe3
47.4338
0.5472
[rel]
20
15
10
5
0
-5
140
120
100
80
60
40
20
[ppm]

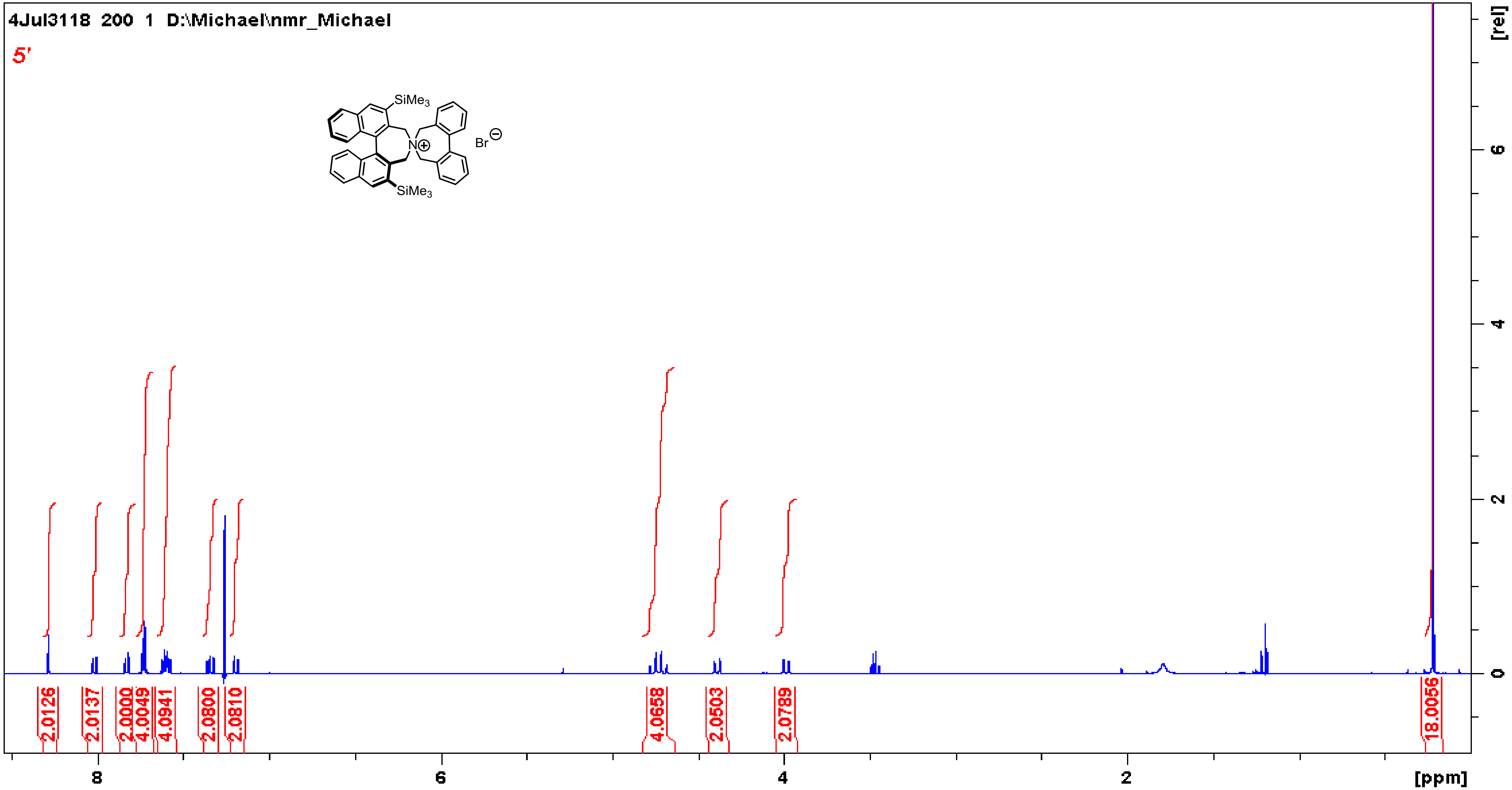
4Jul3118 200 1 D:\Michael\nmr_Michael
5'
SiMe3
N⊕
Br⊖
SiMe3
2.0126
2.0137
2.0000
4.0049
4.0941
2.0800
2.0810
4.0658
2.0503
2.0789
18.0056
8
6
4
2
[ppm]
[rel]
6
4
2
0

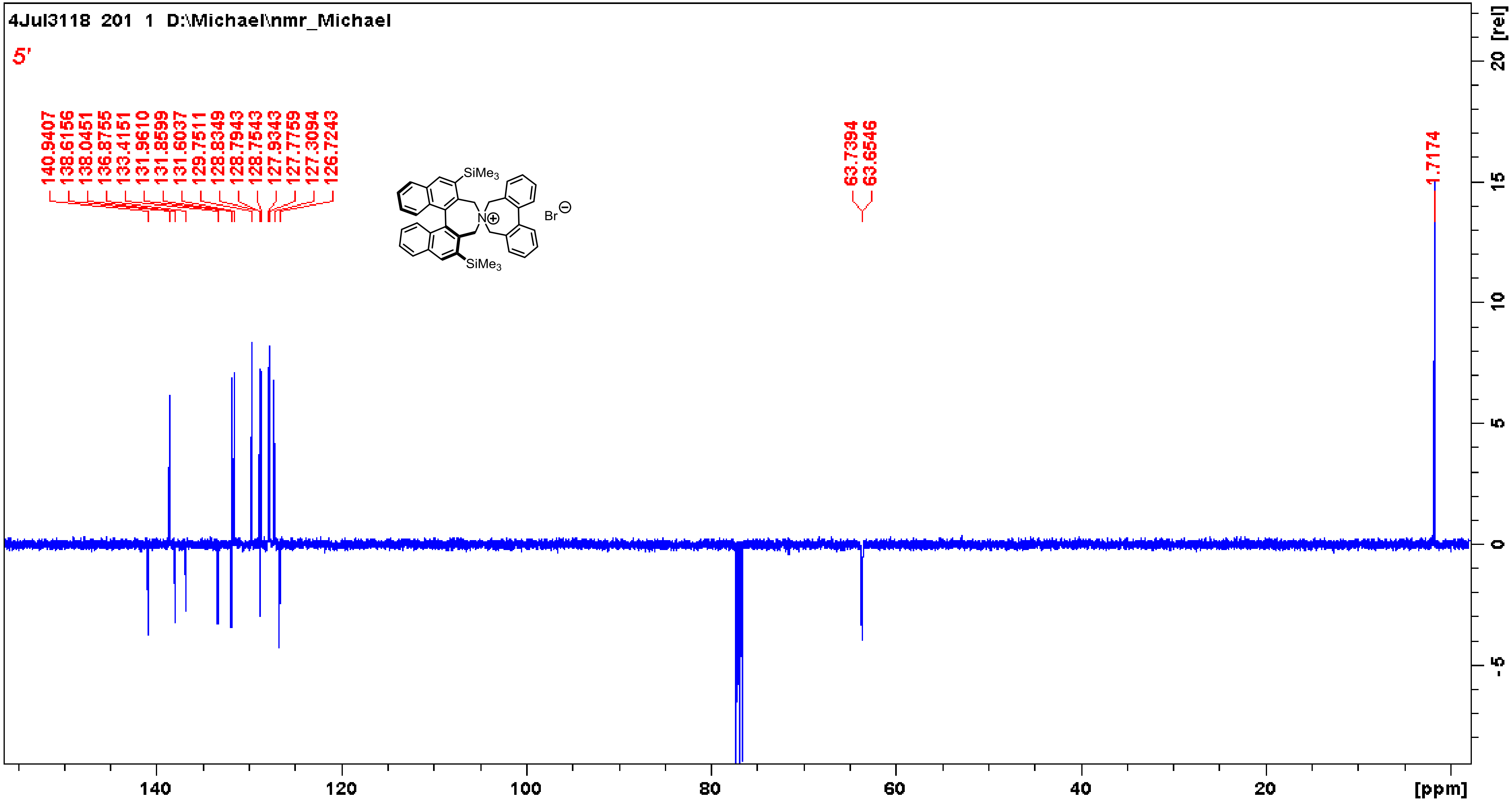
4Jul3118 201 1 D:\Michael\nmr_Michael
5'
140.9407
138.6156
138.0451
136.8755
133.4151
131.9610
131.8599
131.6037
129.7511
128.8349
128.7943
128.7543
127.9343
127.7759
127.3094
126.7243
63.7394
63.6546
1.7174
SiMe3
SiMe3
N⊕
Br⊖
[rel]
20
15
10
5
0
-5
140
120
100
80
60
40
20
[ppm]

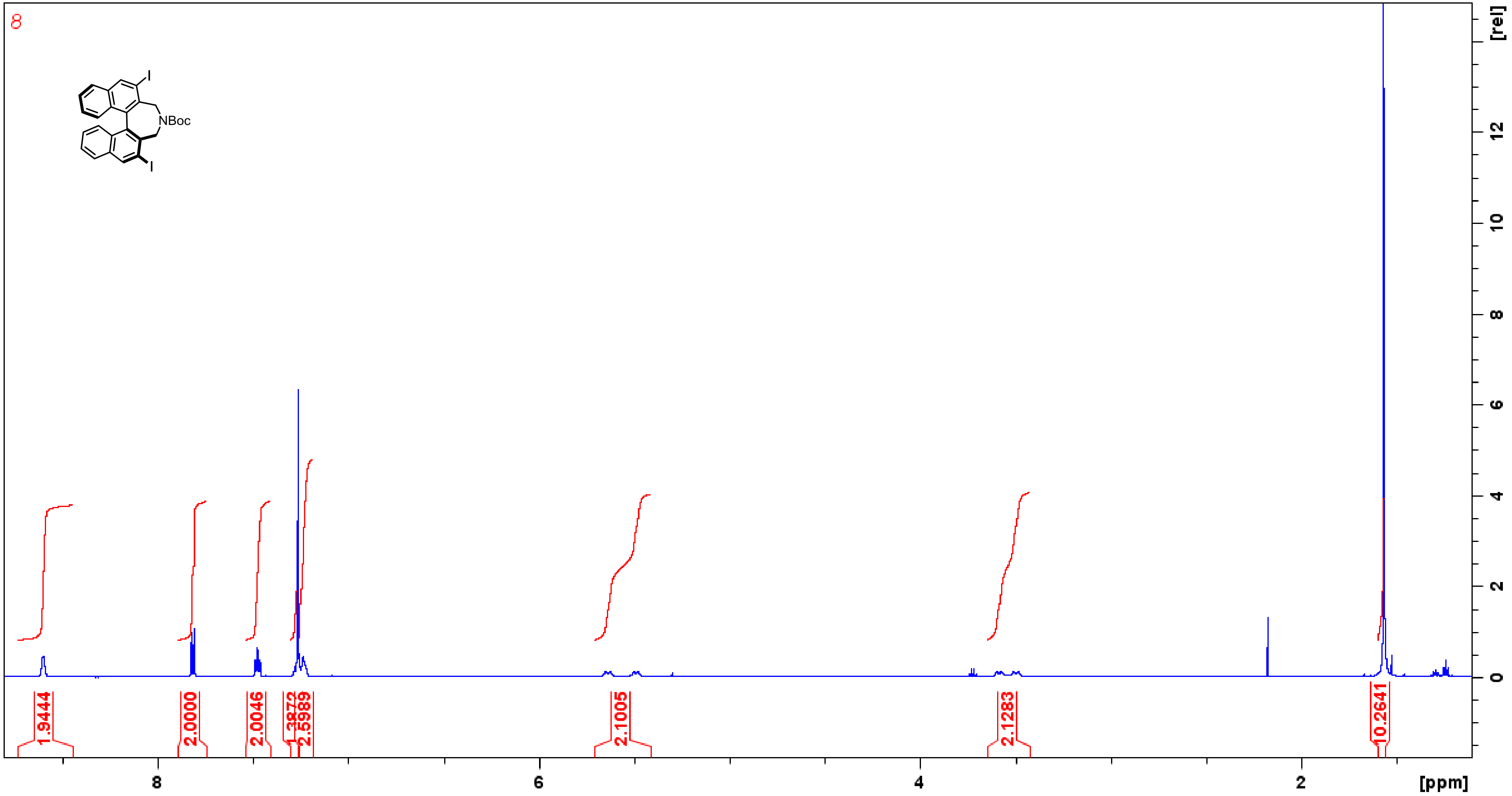

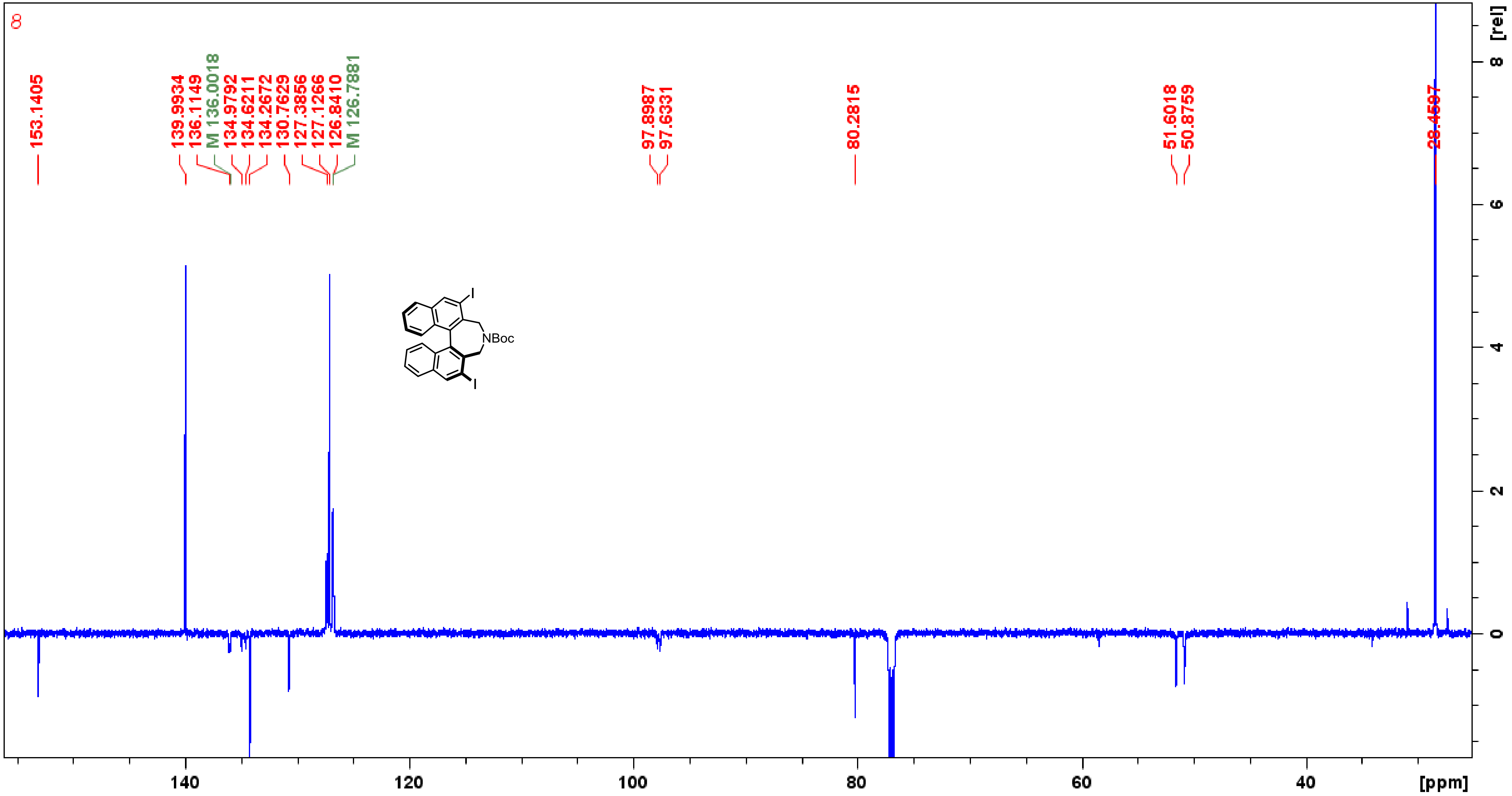
153.1405
139.9934
136.1149
M 136.0018
134.9792
134.6211
134.2672
130.7629
127.3856
127.1266
126.8410
M 126.7881
97.8987
97.6331
80.2815
51.6018
50.8759
28.4587
NBoc
I
[rel]
[ppm]

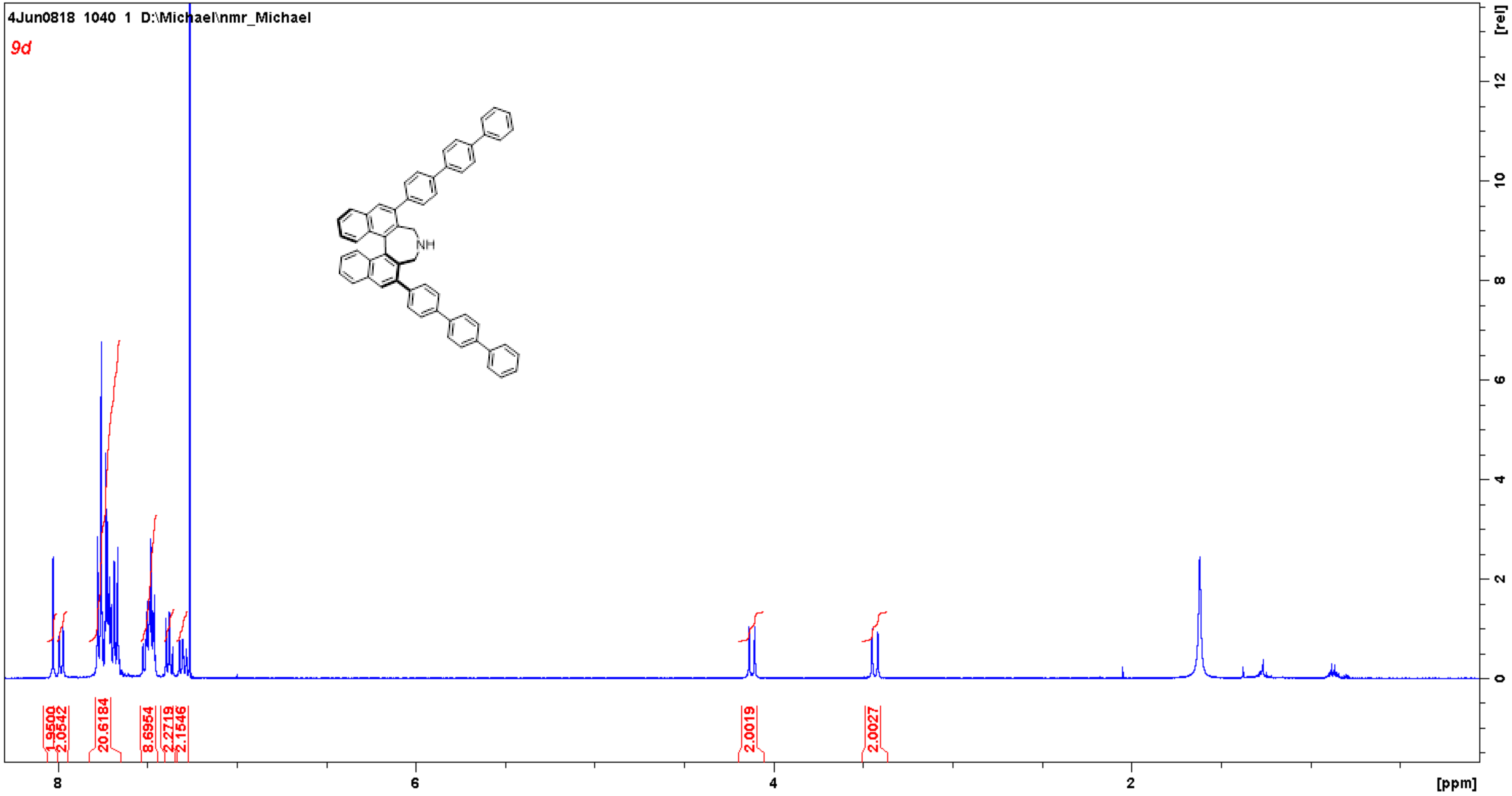
4Jun0818 1040 1 D:\Michael\nmr_Michael
9d
NH
[rel]
12
10
8
6
4
2
0
1.9500
2.0542
20.6184
8.6954
2.2719
2.1546
2.0019
2.0027
8
6
4
2
[ppm]

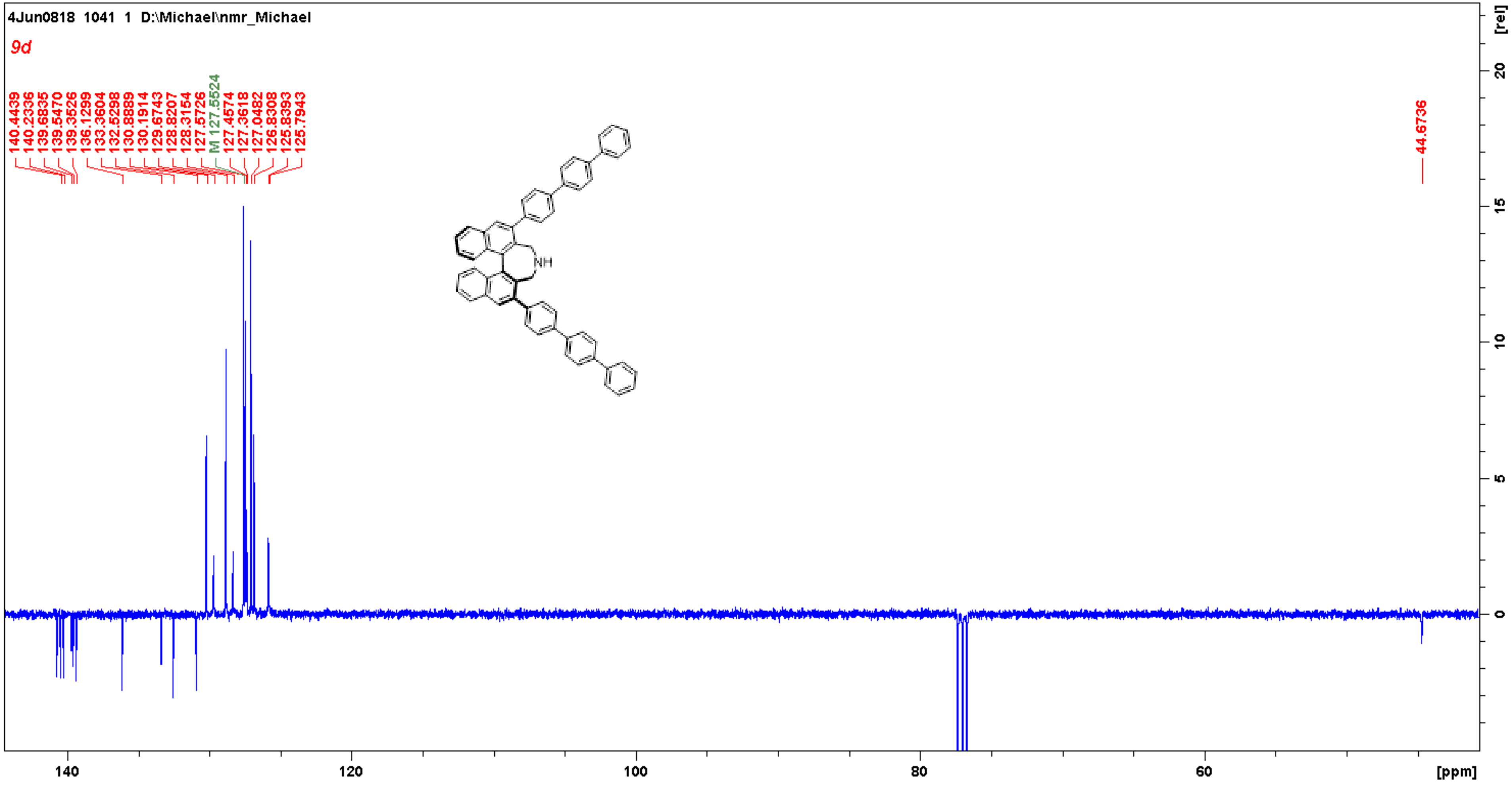
4Jun0818 1041 1 D:\Michael\nmr_Michael
9d
140.4439
140.2336
139.6835
139.5470
139.3526
136.1299
133.3604
132.5298
130.8889
130.1914
129.6743
128.8207
128.3154
127.5726
M 127.5524
127.4574
127.3618
127.0482
126.8308
125.8393
125.7943
44.6736
NH
[rel]
20
15
10
5
0
140
120
100
80
60
[ppm]

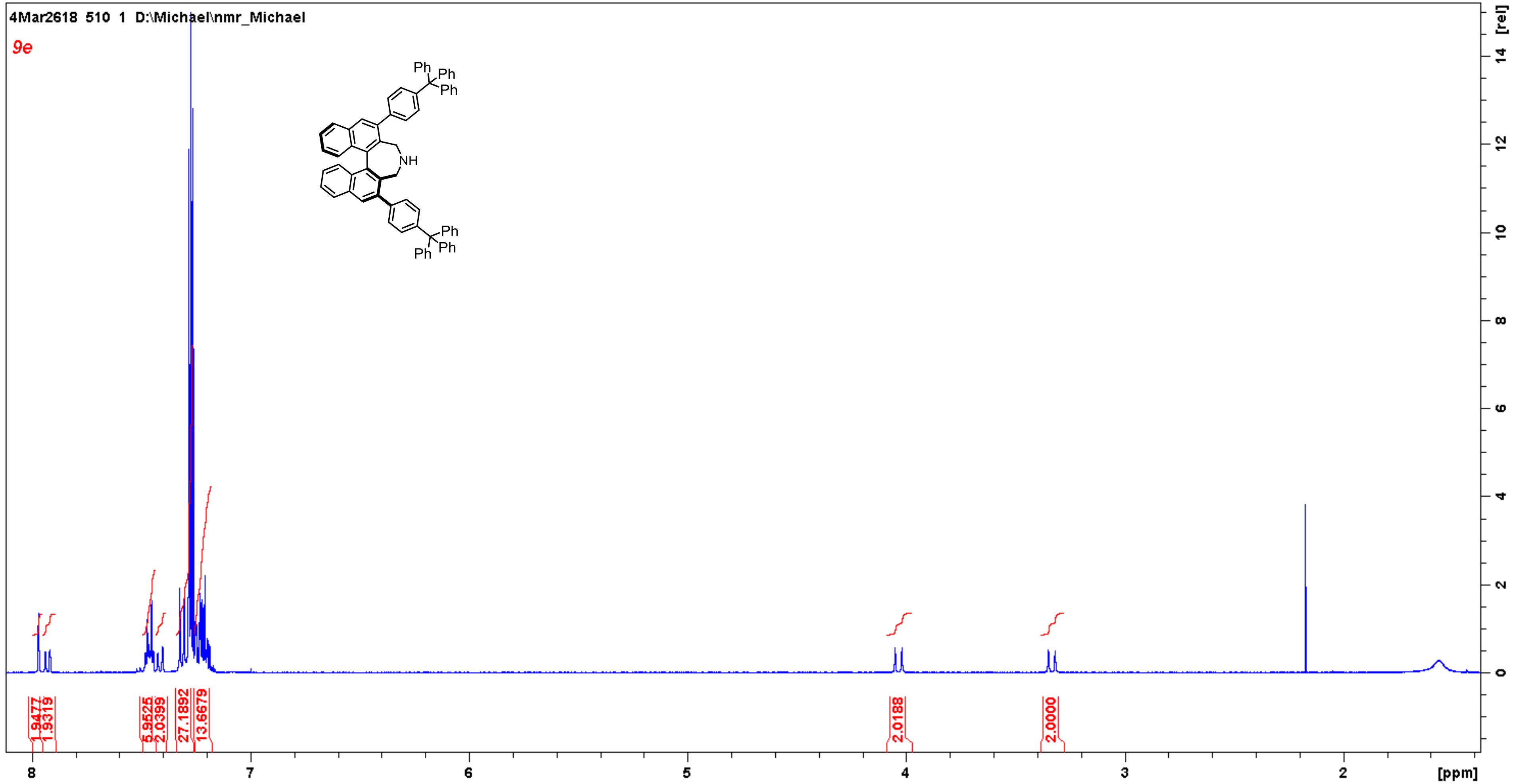
4Mar2618 510 1 D:\Michael\nmr_Michael
9e
Ph
Ph
Ph
NH
Ph
Ph
Ph
1.9477
1.9319
5.9525
2.0399
27.1892
13.6679
2.0188
2.0000
[rel]
[ppm]

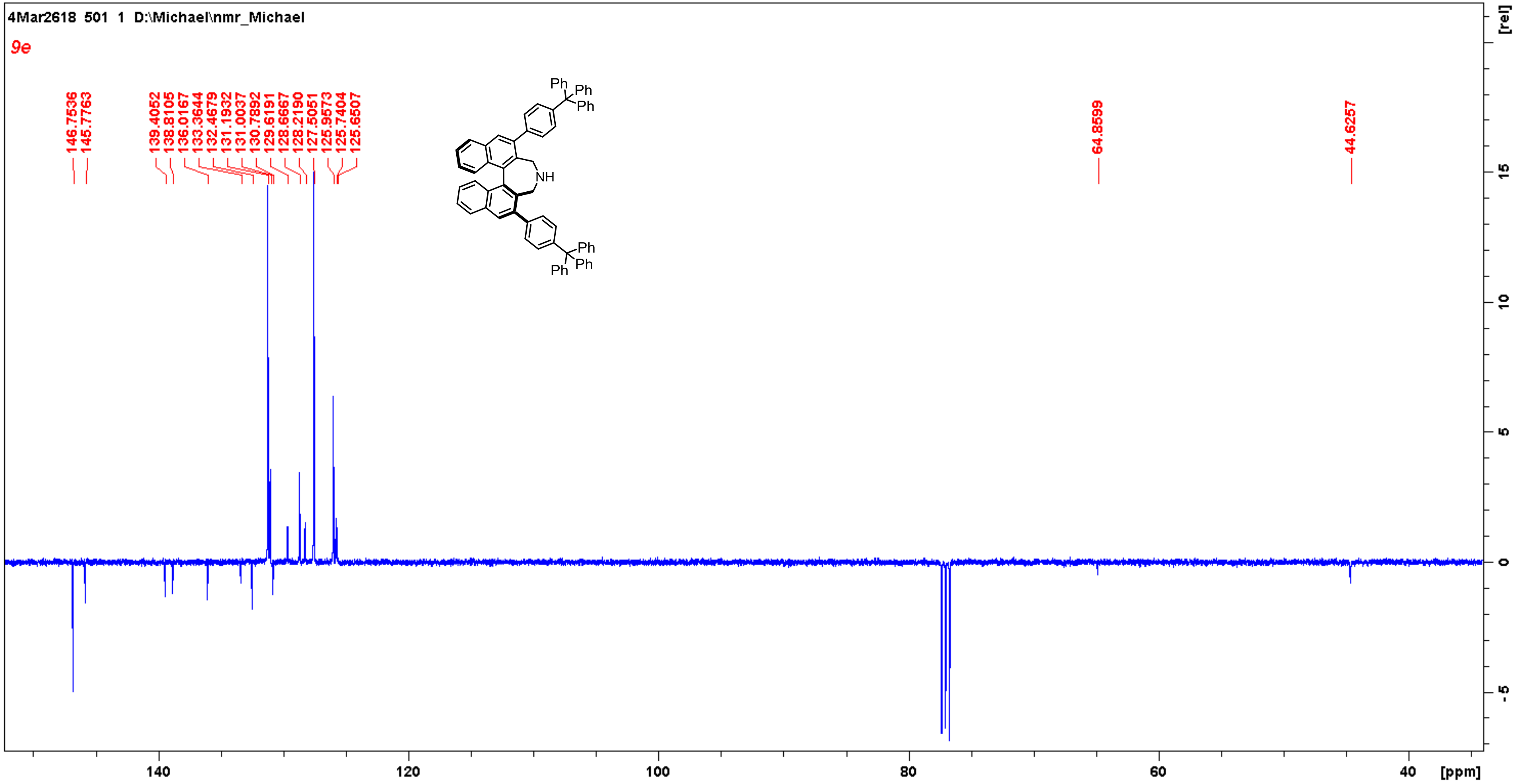
4Mar2618 501 1 D:\Michael\nmr_Michael
9e
146.7536
145.7763
139.4052
138.8105
136.0167
133.3644
132.4679
131.1932
131.0037
130.7892
129.6191
128.6667
128.2190
127.5051
125.9573
125.7404
125.6507
64.8599
44.6257
NH
Ph
Ph
Ph
Ph
Ph
Ph
[rel]
15
10
5
0
-5
140
120
100
80
60
40
[ppm]

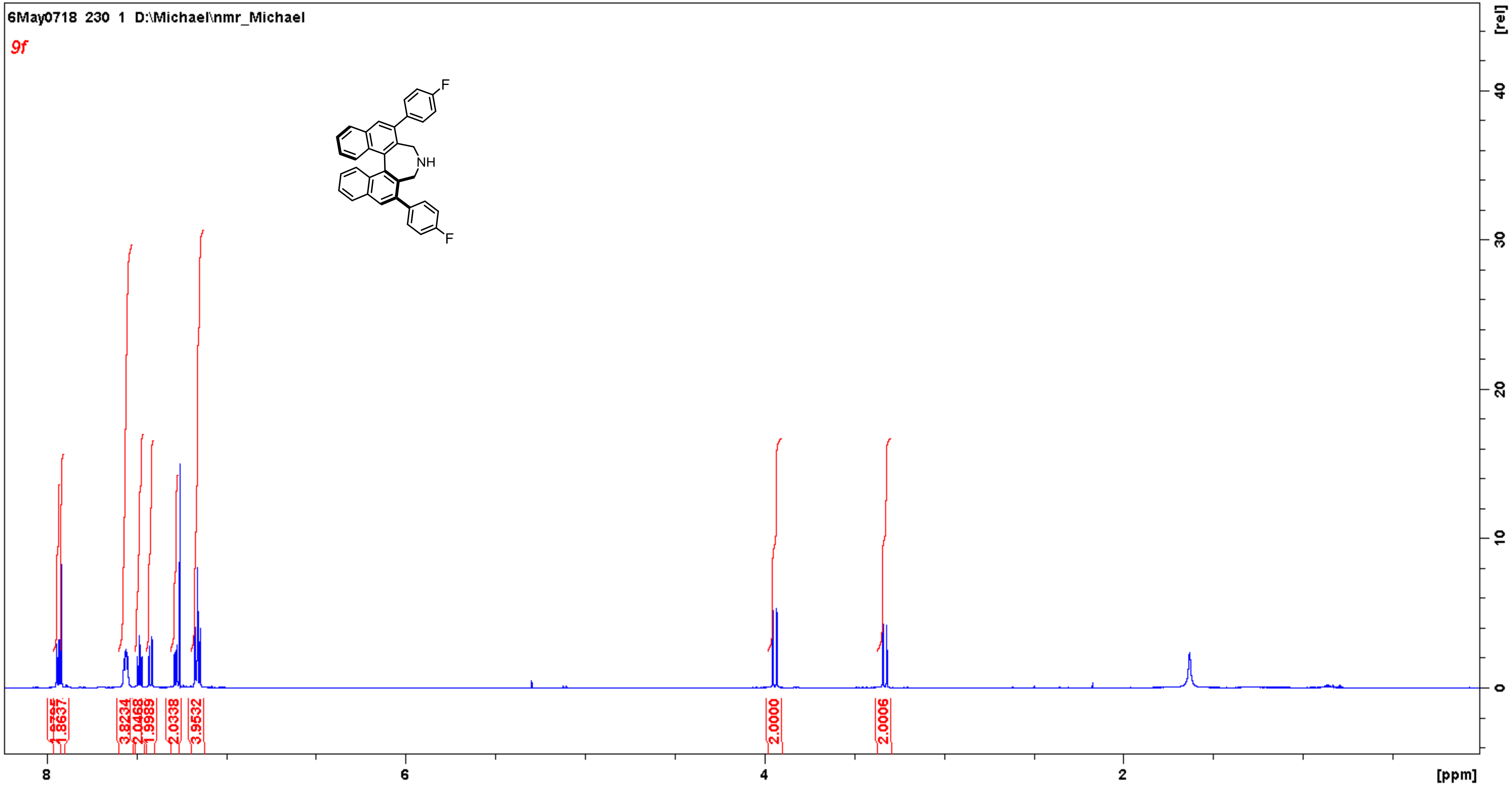
6May0718 230 1 D:\Michael\nmr_Michael
9f
F
NH
F
1.8637
3.8234
2.0468
1.9989
2.0338
3.9532
2.0000
2.0006
8
6
4
2
[ppm]
[rel]
40
30
20
10
0

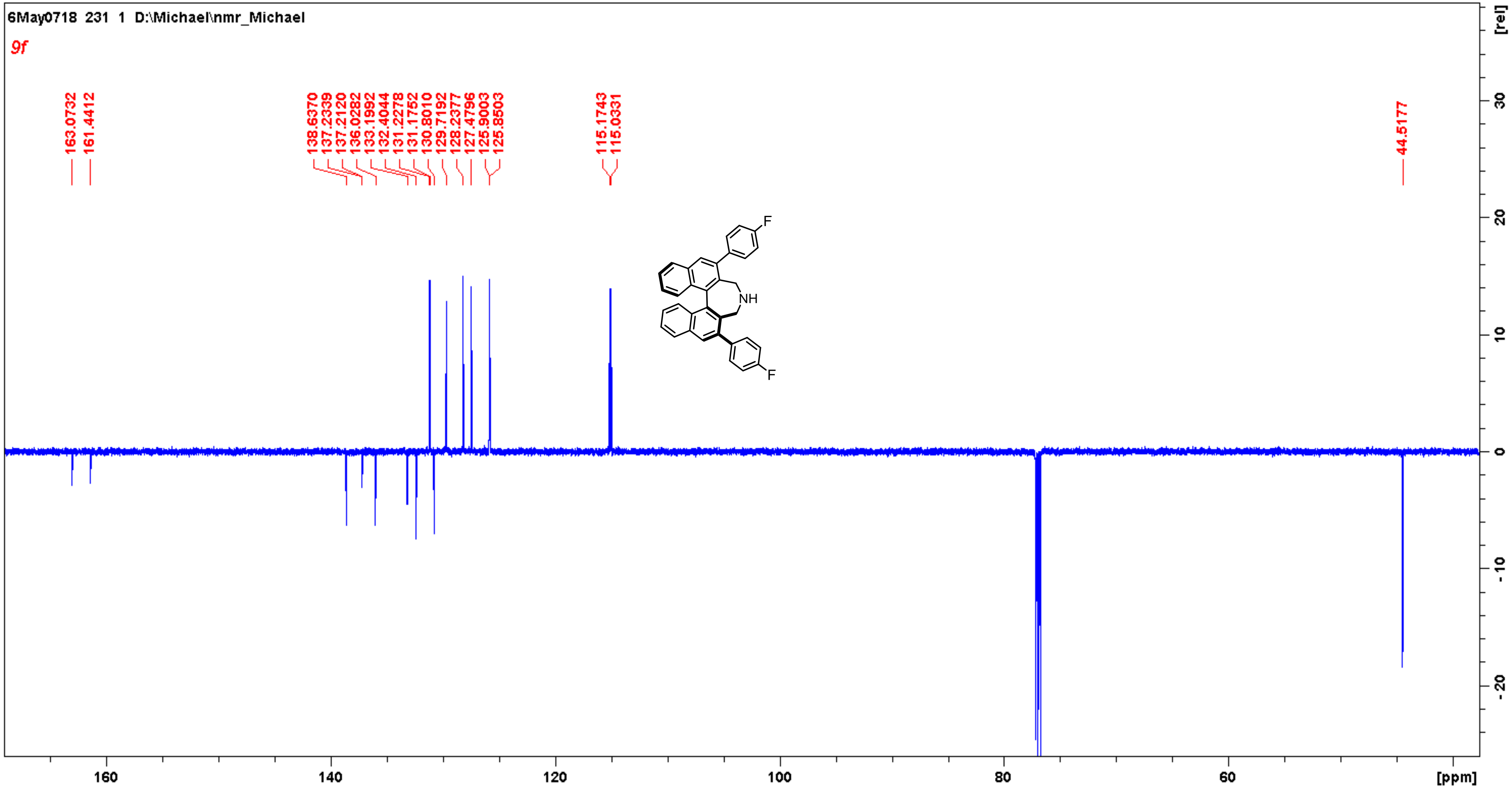

6May0718 231 1 D:\Michael\nmr_Michael
9f
163.0732
161.4412
138.6370
137.2339
137.2120
136.0282
133.1992
132.4044
131.2278
131.1752
130.8010
129.7192
128.2377
127.4796
125.9003
125.8503
115.1743
115.0331
44.5177
F
NH
F
[rel]
30
20
10
0
-10
-20
160
140
120
100
80
60
[ppm]

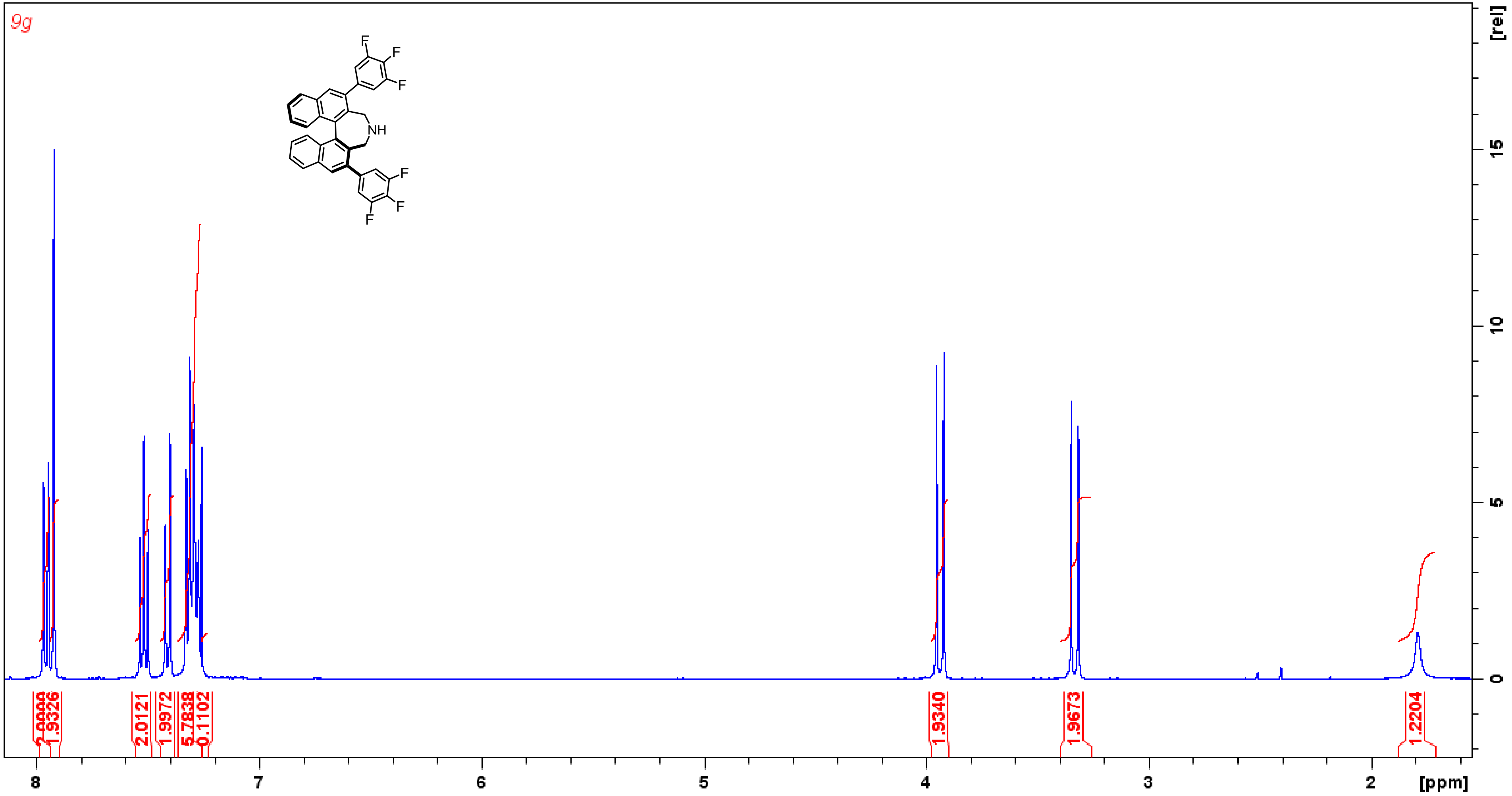
9g
F
F
F
NH
F
F
F
1.9326
2.0121
1.9972
5.7838
0.1102
1.9340
1.9673
1.2204
[rel]
15
10
5
0
8
7
6
5
4
3
2
[ppm]

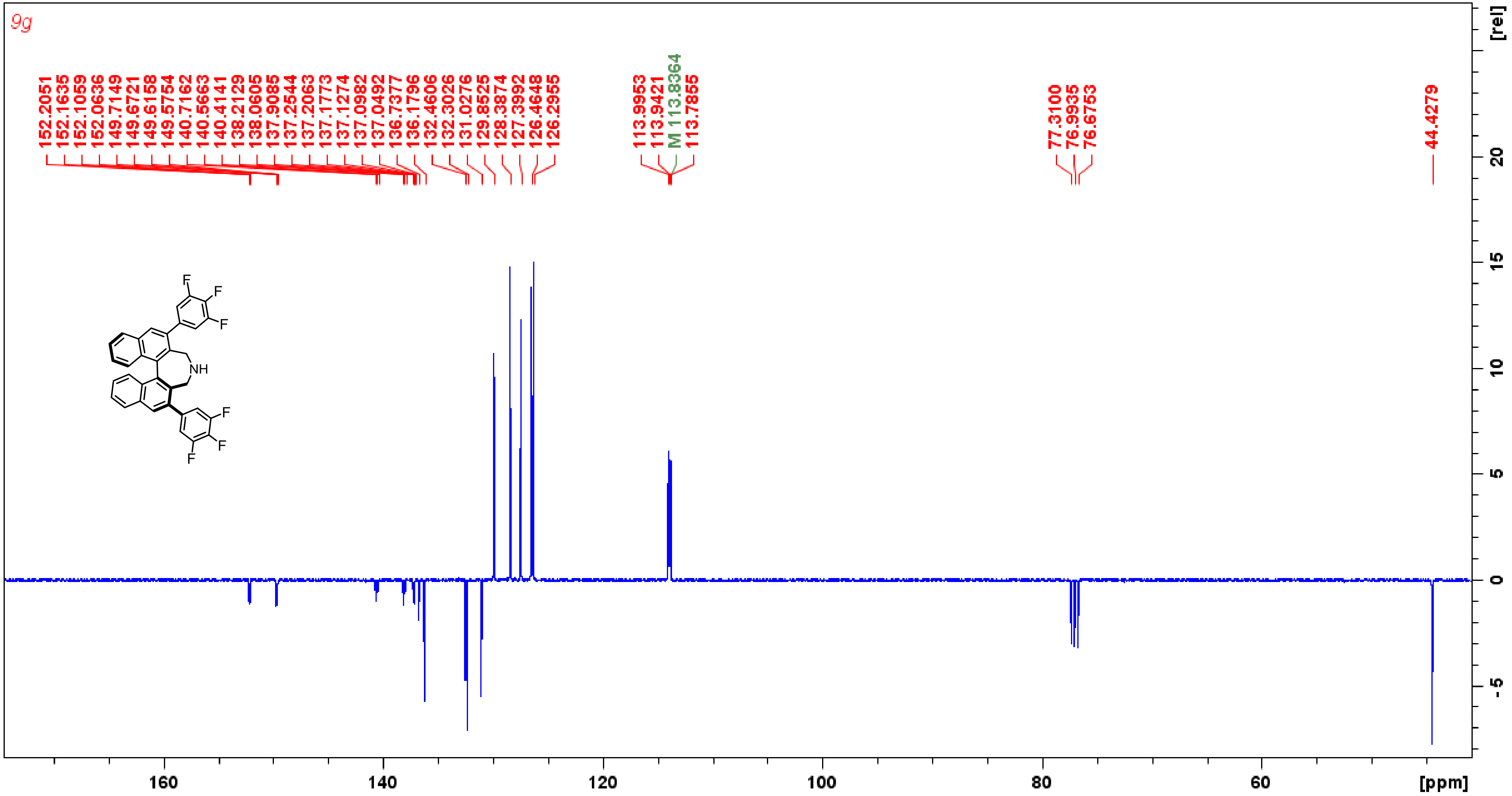
9g
152.2051
152.1635
152.1059
152.0636
149.7149
149.6721
149.6158
149.5754
140.7162
140.5663
140.4141
138.2129
138.0605
137.9085
137.2544
137.2063
137.1773
137.1274
137.0982
137.0492
136.7377
136.1796
132.4606
132.3026
131.0276
129.8525
128.3874
127.3992
126.4648
126.2955
113.9953
113.9421
M 113.8364
113.7855
77.3100
76.9935
76.6753
44.4279
F
F
F
NH
F
F
F
[rel]
20
15
10
5
0
-5
160
140
120
100
80
60
[ppm]

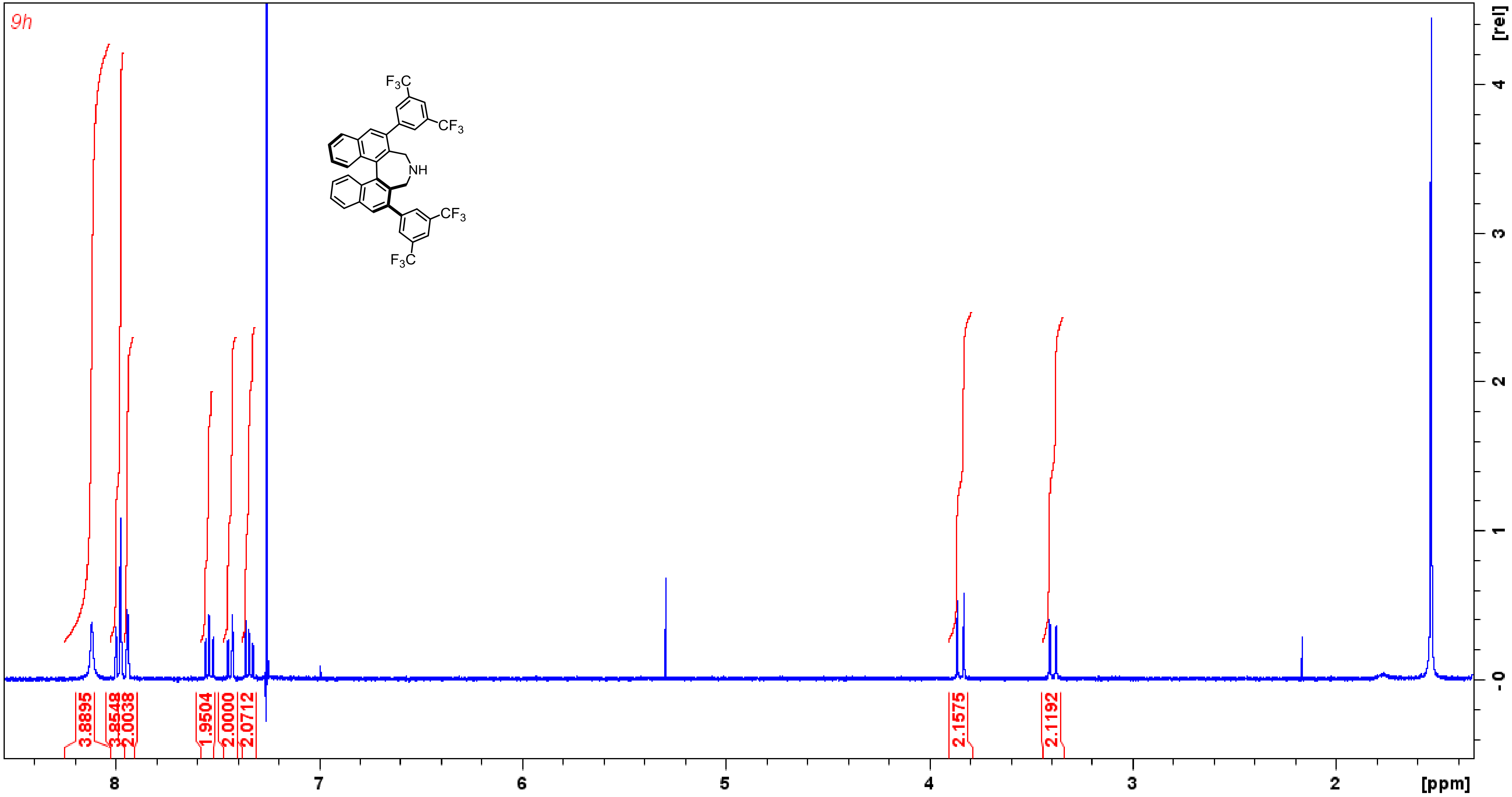
9h
NH
F_3C
CF_3
CF_3
F_3C
3.8895
3.8548
2.0038
1.9504
2.0000
2.0712
2.1575
2.1192
[rel]
[ppm]

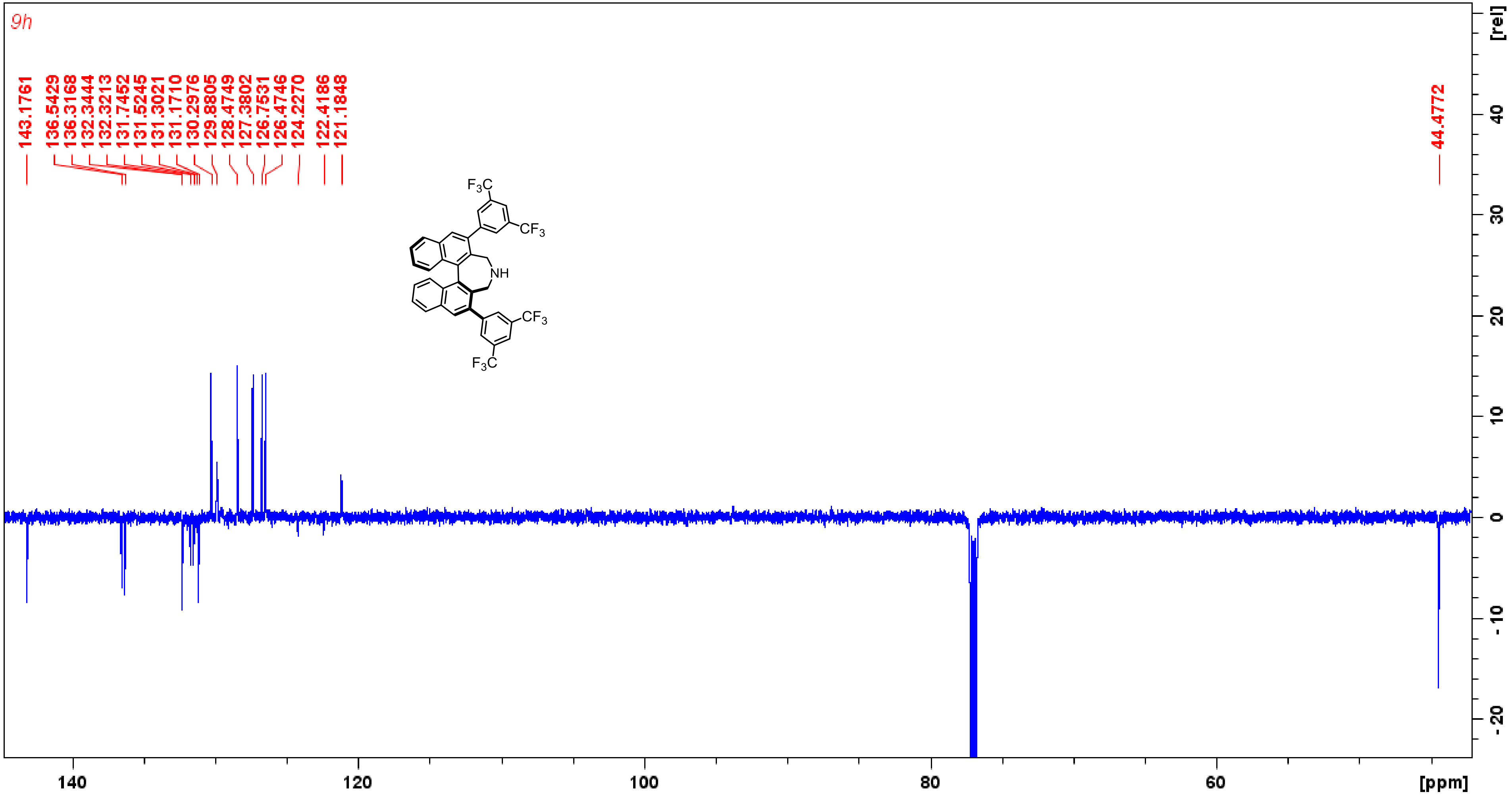
9h
143.1761
136.5429
136.3168
132.3444
132.3213
131.7452
131.5245
131.3021
131.1710
130.2976
129.8805
128.4749
127.3802
126.7531
126.4746
124.2270
122.4186
121.1848
44.4772
F3C
CF3
NH
CF3
F3C
[rel]
40
30
20
10
0
-10
-20
140
120
100
80
60
[ppm]

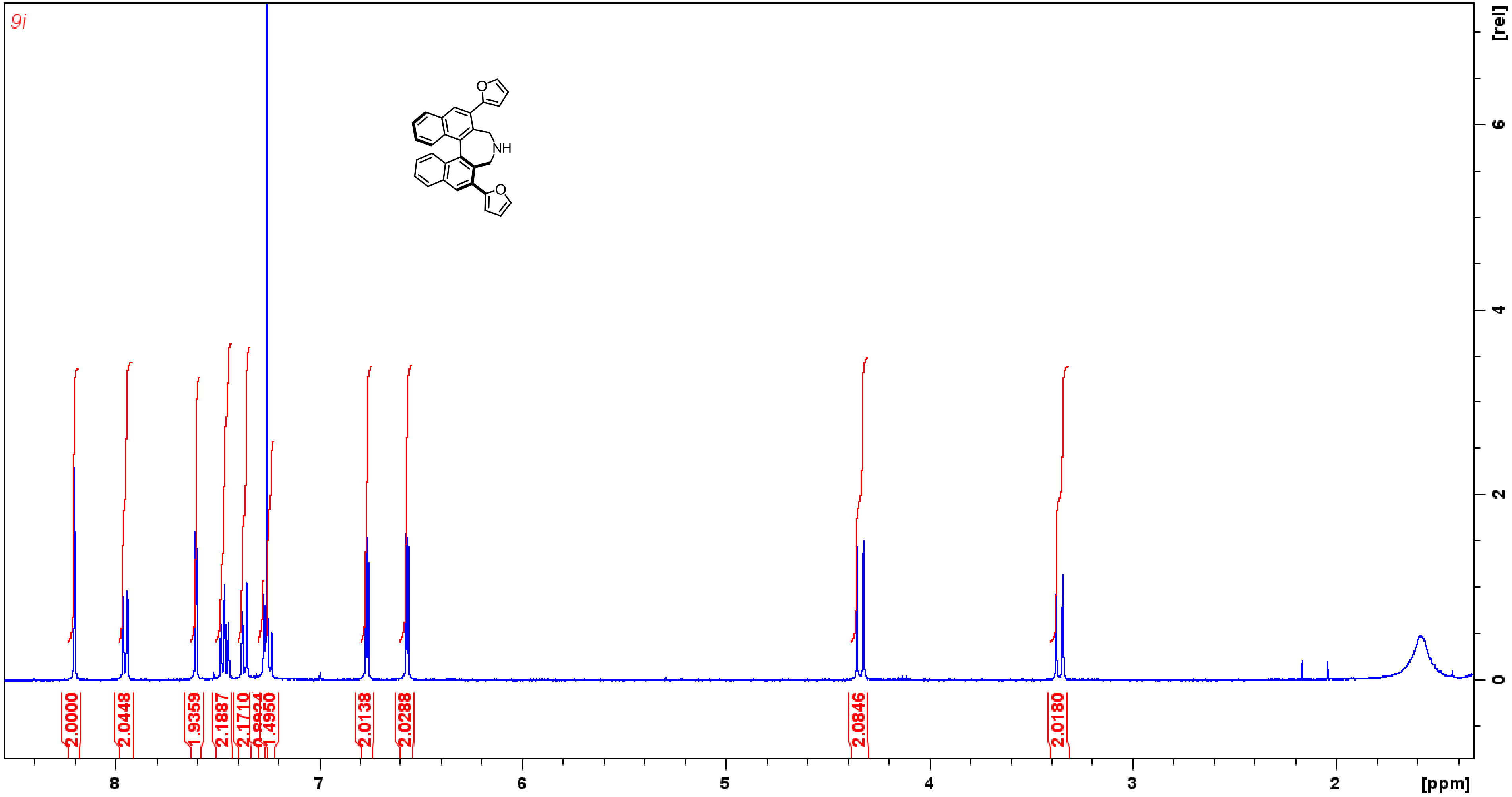
9i
O
NH
O
2.0000
2.0448
1.9359
2.1887
2.1710
1.4950
2.0138
2.0288
2.0846
2.0180
8
7
6
5
4
3
2
[ppm]
[rel]
0
2
4
6

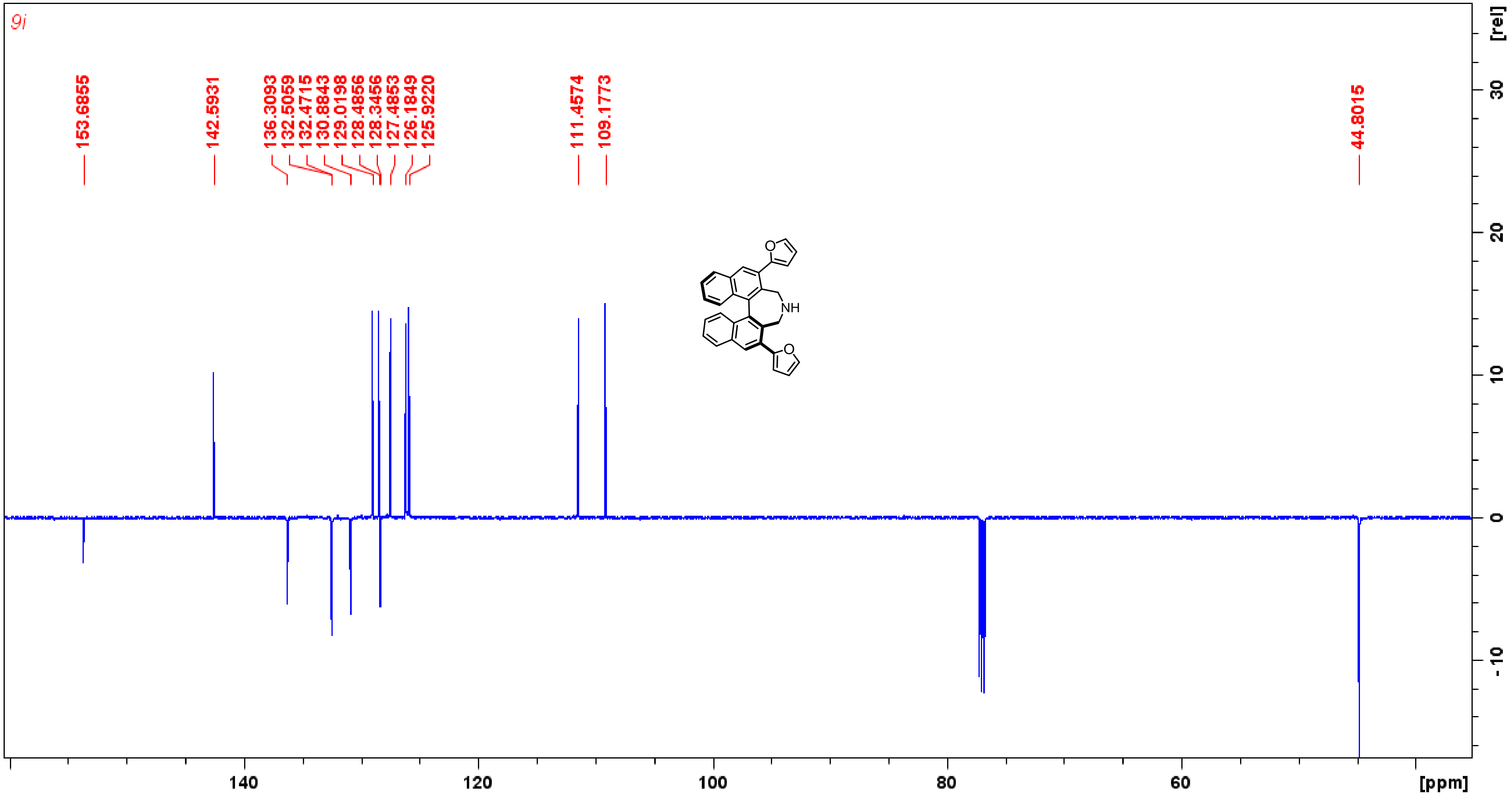
9i
153.6855
142.5931
136.3093
132.5059
132.4715
130.8843
129.0198
128.4856
128.3456
127.4853
126.1849
125.9220
111.4574
109.1773
44.8015
NH
O
O
[rel]
30
20
10
0
-10
140
120
100
80
60
[ppm]

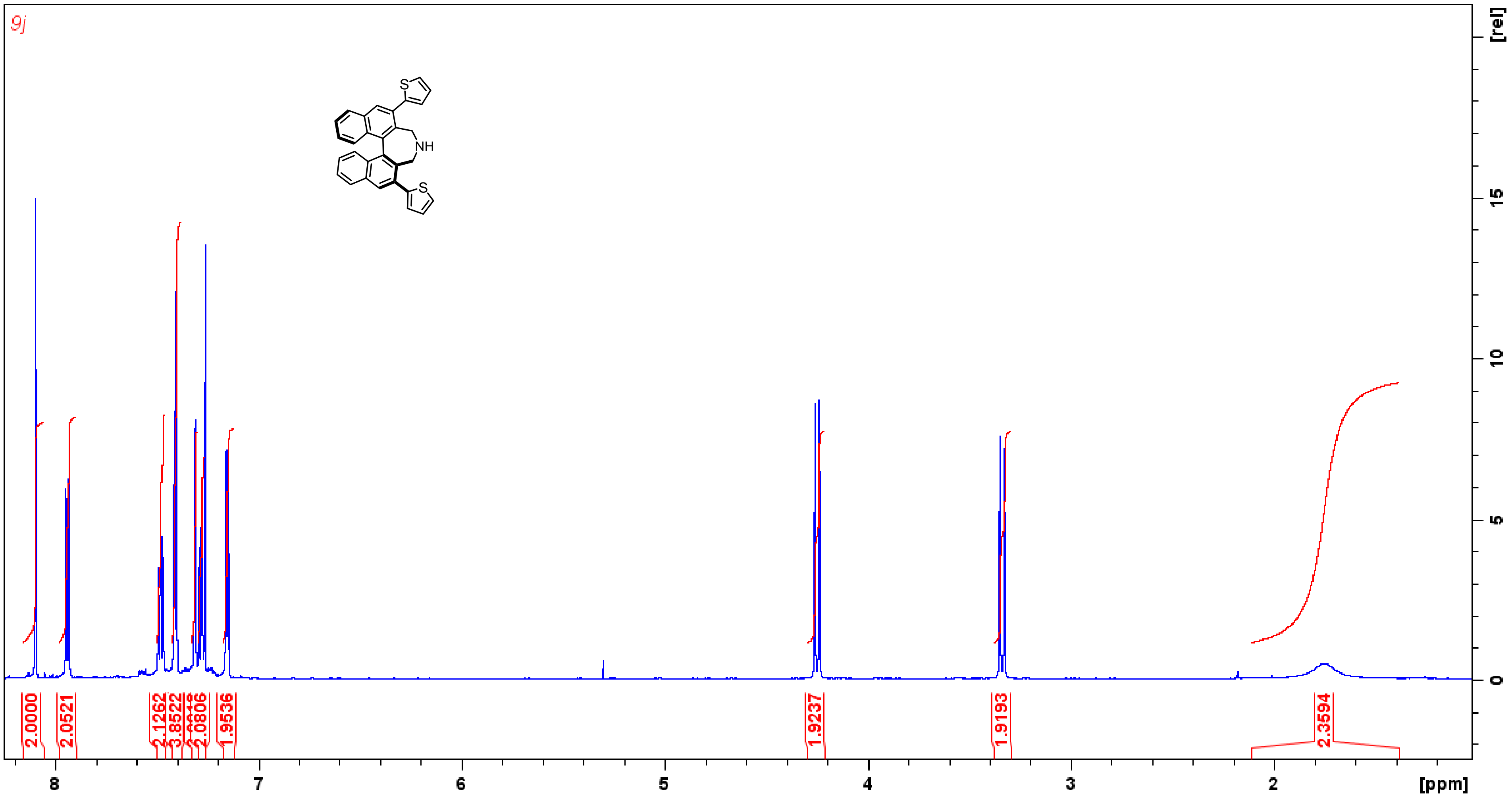
9j
NH
S
S
2.0000
2.0521
2.1262
3.8522
2.0806
1.9536
1.9237
1.9193
2.3594
8
7
6
5
4
3
2
[ppm]
[rel]
15
10
5
0

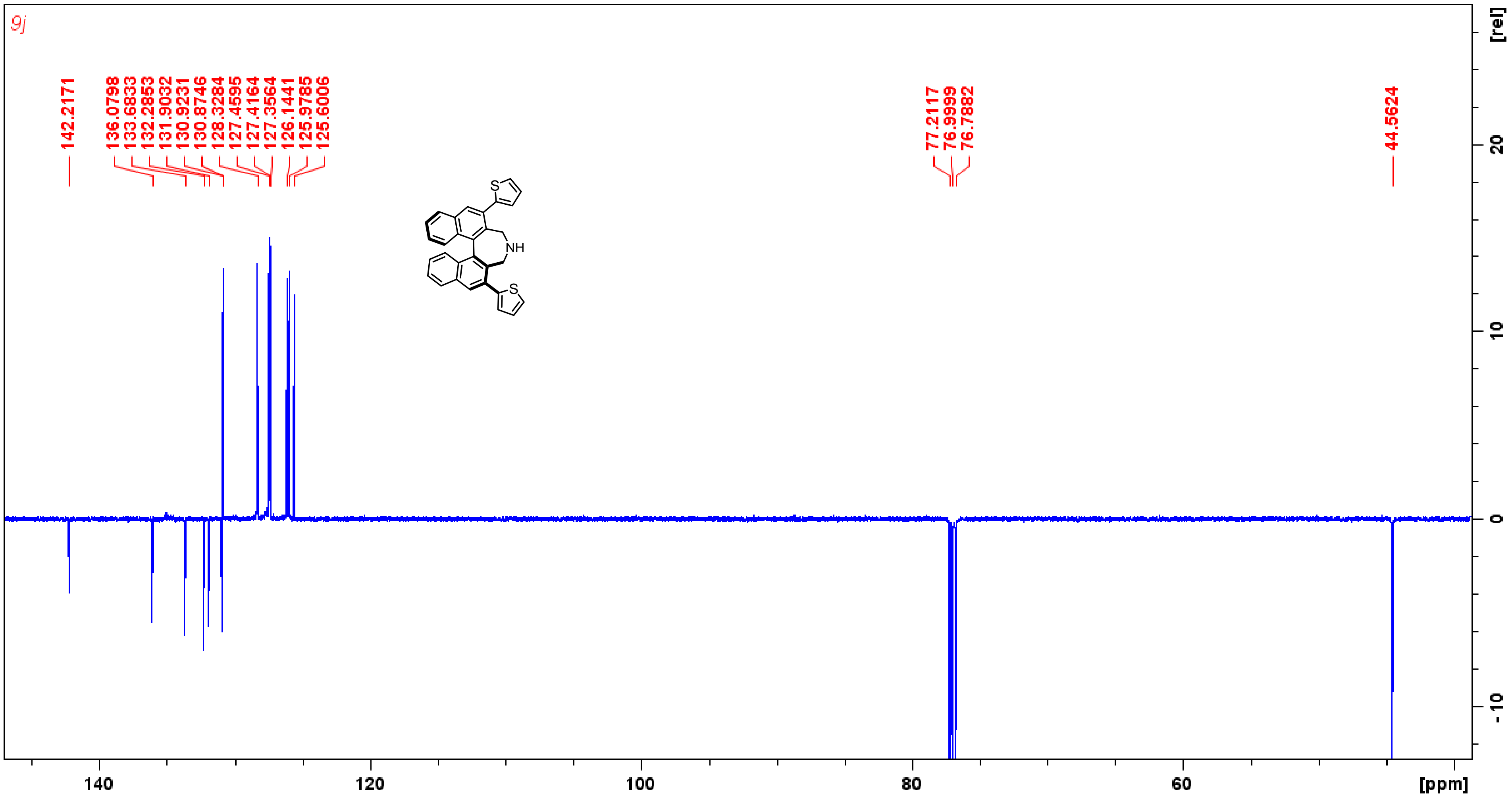
9j
142.2171
136.0798
133.6833
132.2853
131.9032
130.9231
130.8746
128.3284
127.4595
127.4164
127.3564
126.1441
125.9785
125.6006
77.2117
76.9999
76.7882
44.5624
NH
S
S
[rel]
20
10
0
-10
140
120
100
80
60
[ppm]

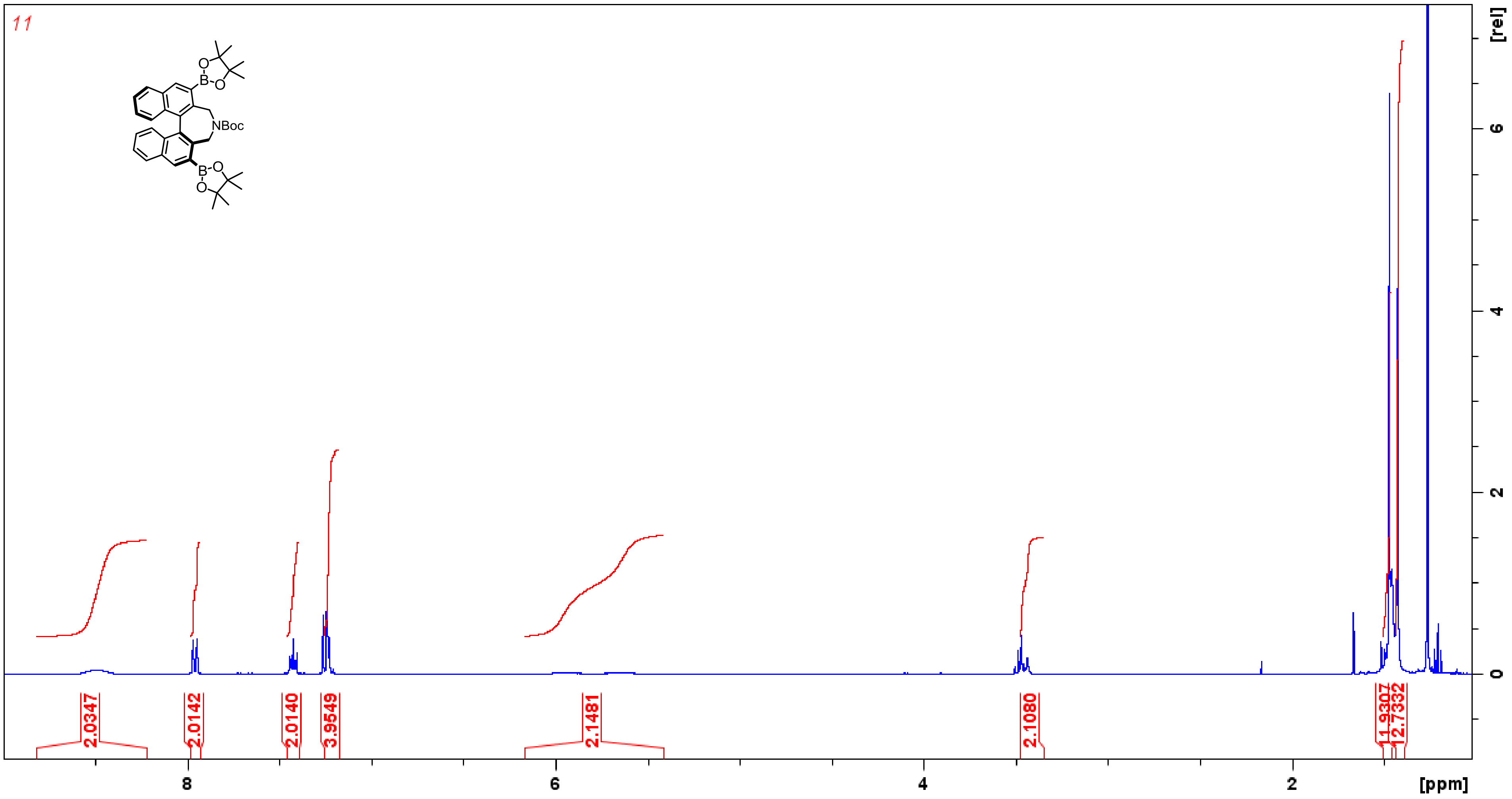
11
NBoc
[rel]
[ppm]
2.0347
2.0142
2.0140
3.9549
2.1481
2.1080
11.9307
12.7332

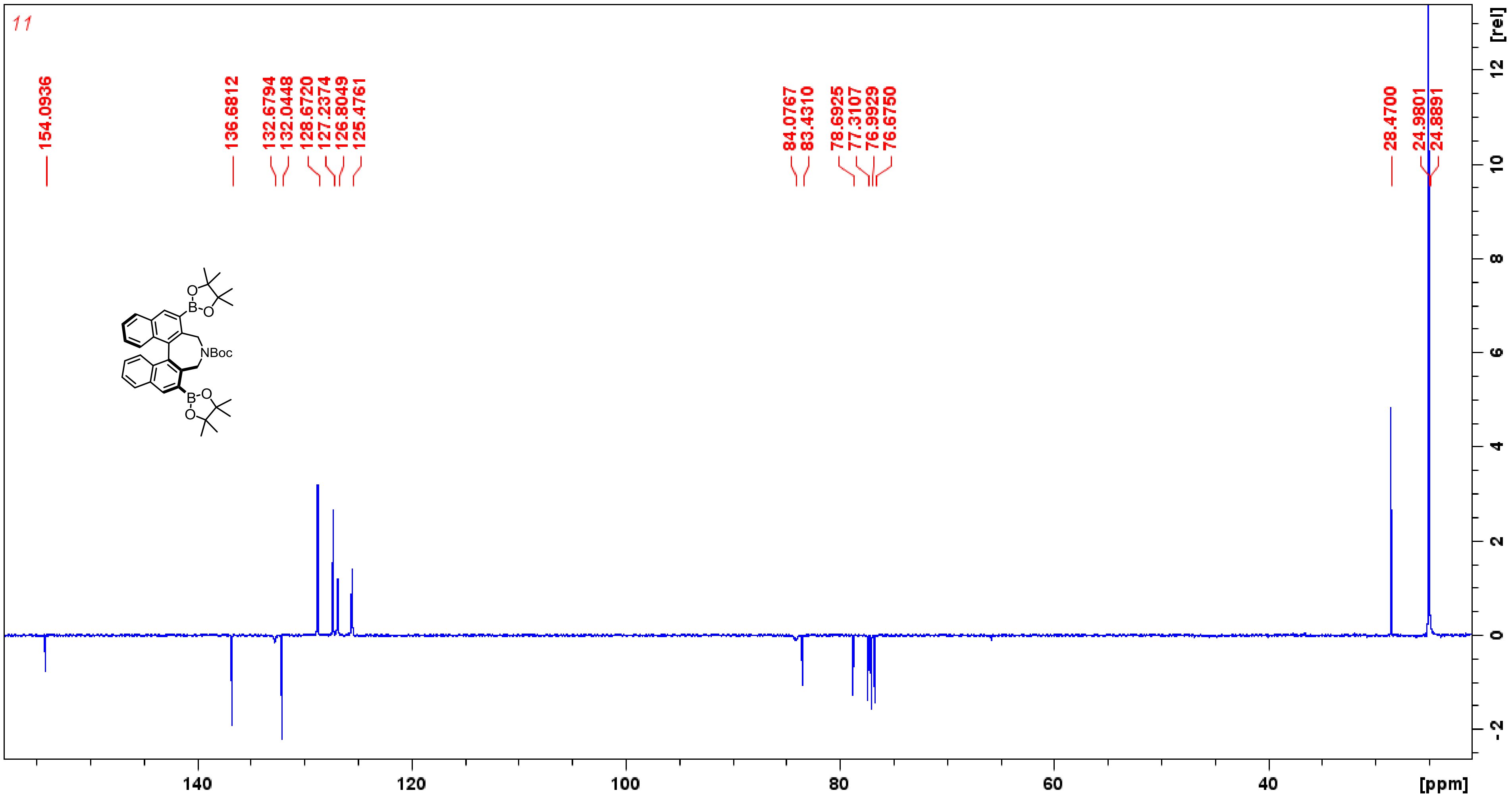
11
154.0936
136.6812
132.6794
132.0448
128.6720
127.2374
126.8049
125.4761
84.0767
83.4310
78.6925
77.3107
76.9929
76.6750
28.4700
24.9801
24.8891
NBoc
B
O
[rel]
[ppm]
140
120
100
80
60
40

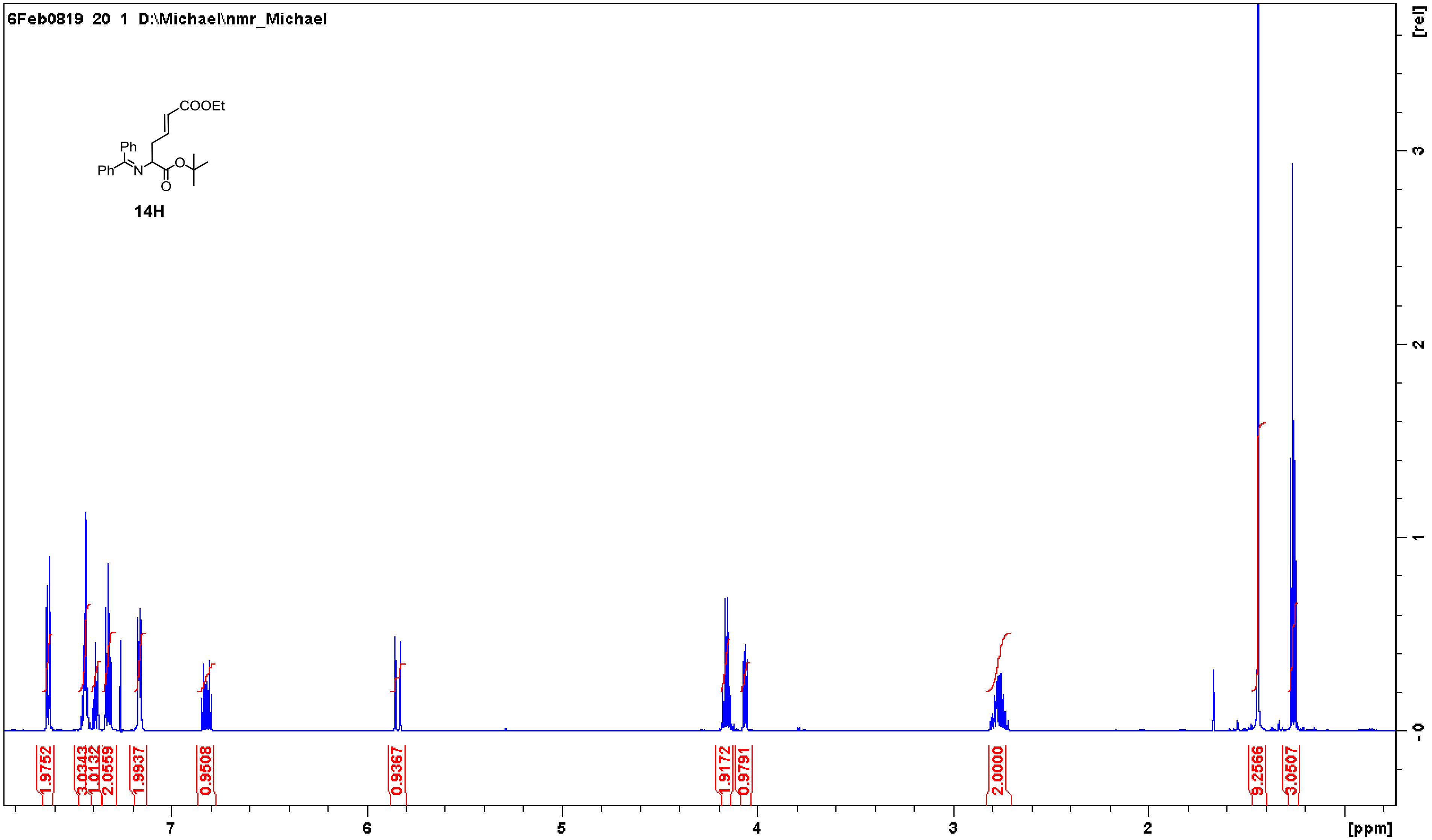
6Feb0819 20 1 D:\Michael\nmr_Michael
COOEt
Ph
Ph
N
O
O
14H
1.9752
3.0343
1.0132
2.0559
1.9937
0.9508
0.9367
1.9172
0.9791
2.0000
9.2566
3.0507
7
6
5
4
3
2
[ppm]
[rel]
3
2
1
0

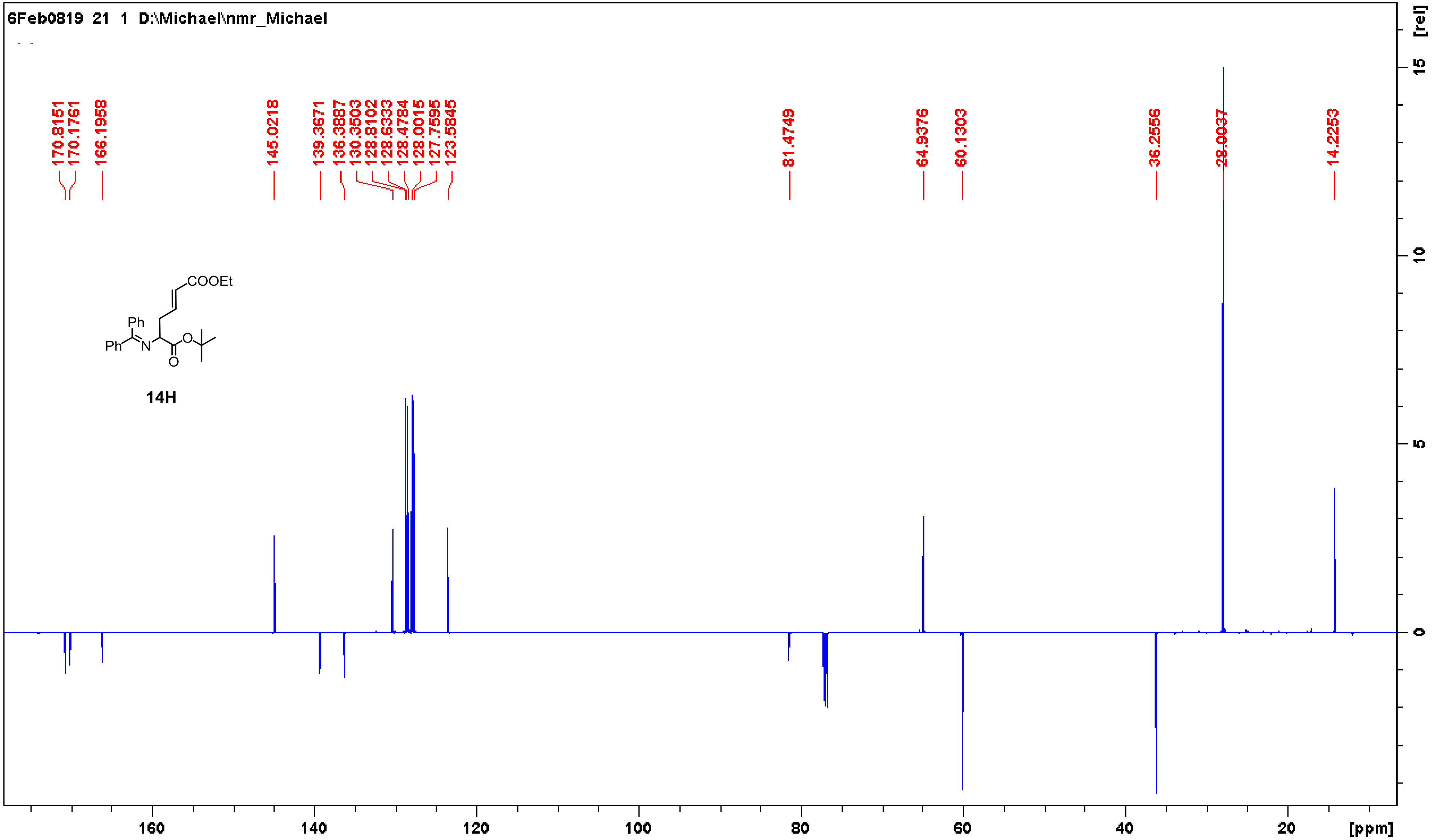
6Feb0819 21 1 D:\Michael\nmr_Michael
170.8151
170.1761
166.1958
145.0218
139.3671
136.3887
130.3503
128.8102
128.6333
128.4784
128.0015
127.7595
123.5845
81.4749
64.9376
60.1303
36.2556
28.0037
14.2253
COOEt
Ph
Ph
N
O
O
14H
[rel]
[ppm]

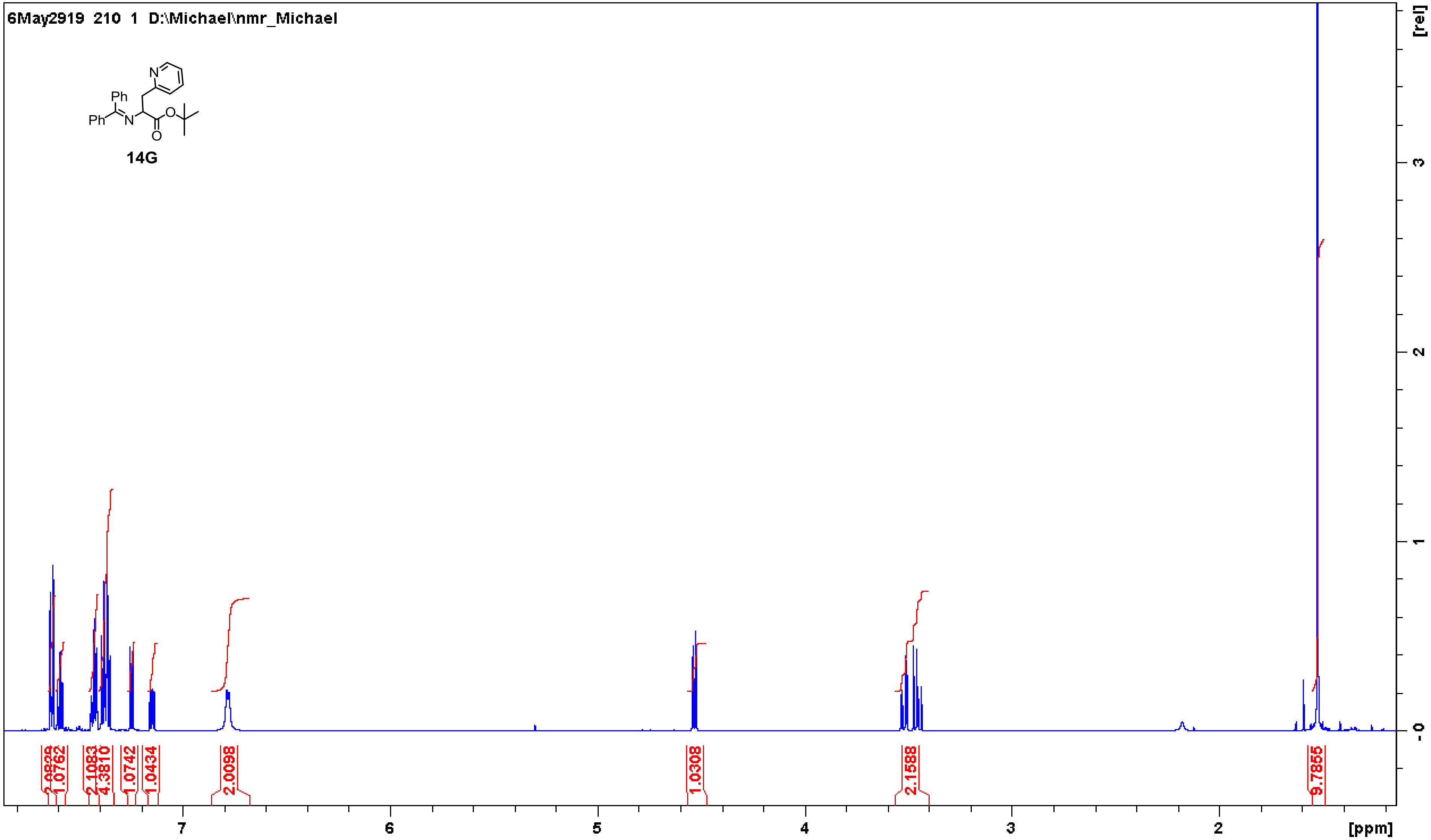
14G
6May2919 210 1 D:\Michael\nmr_Michael
[rel]
[ppm]

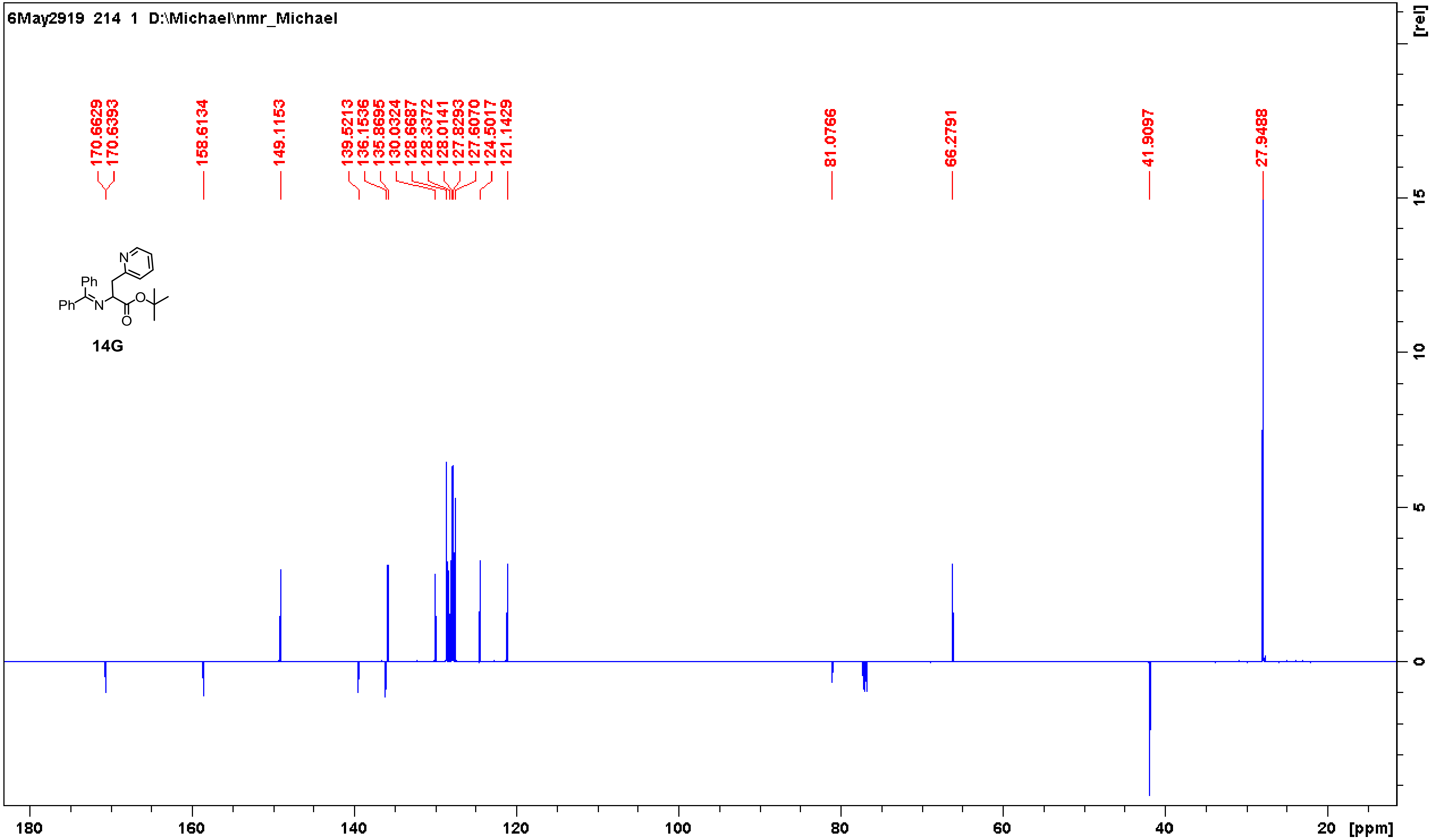
6May2919 214 1 D:\Michael\nmr_Michael
170.6629
170.6393
158.6134
149.1153
139.5213
136.1536
135.8695
130.0324
128.6687
128.3372
128.0141
127.8293
127.6070
124.5017
121.1429
81.0766
66.2791
41.9097
27.9488
14G
[rel]
[ppm]

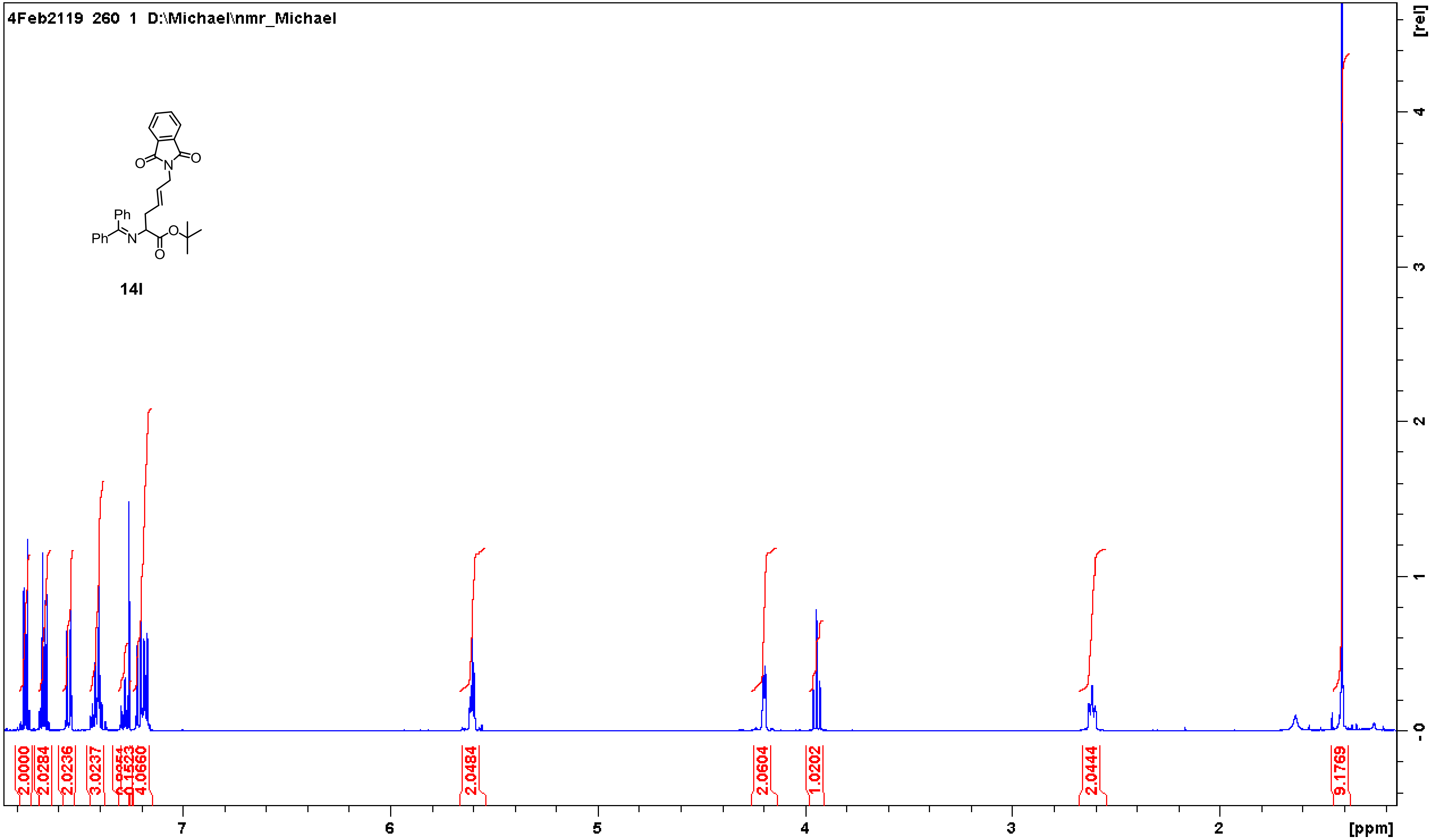
4Feb2119 260 1 D:\Michael\nmr_Michael
14l
2.0000
2.0284
2.0236
3.0237
4.0660
2.0484
2.0604
1.0202
2.0444
9.1769
[ppm]
[rel]

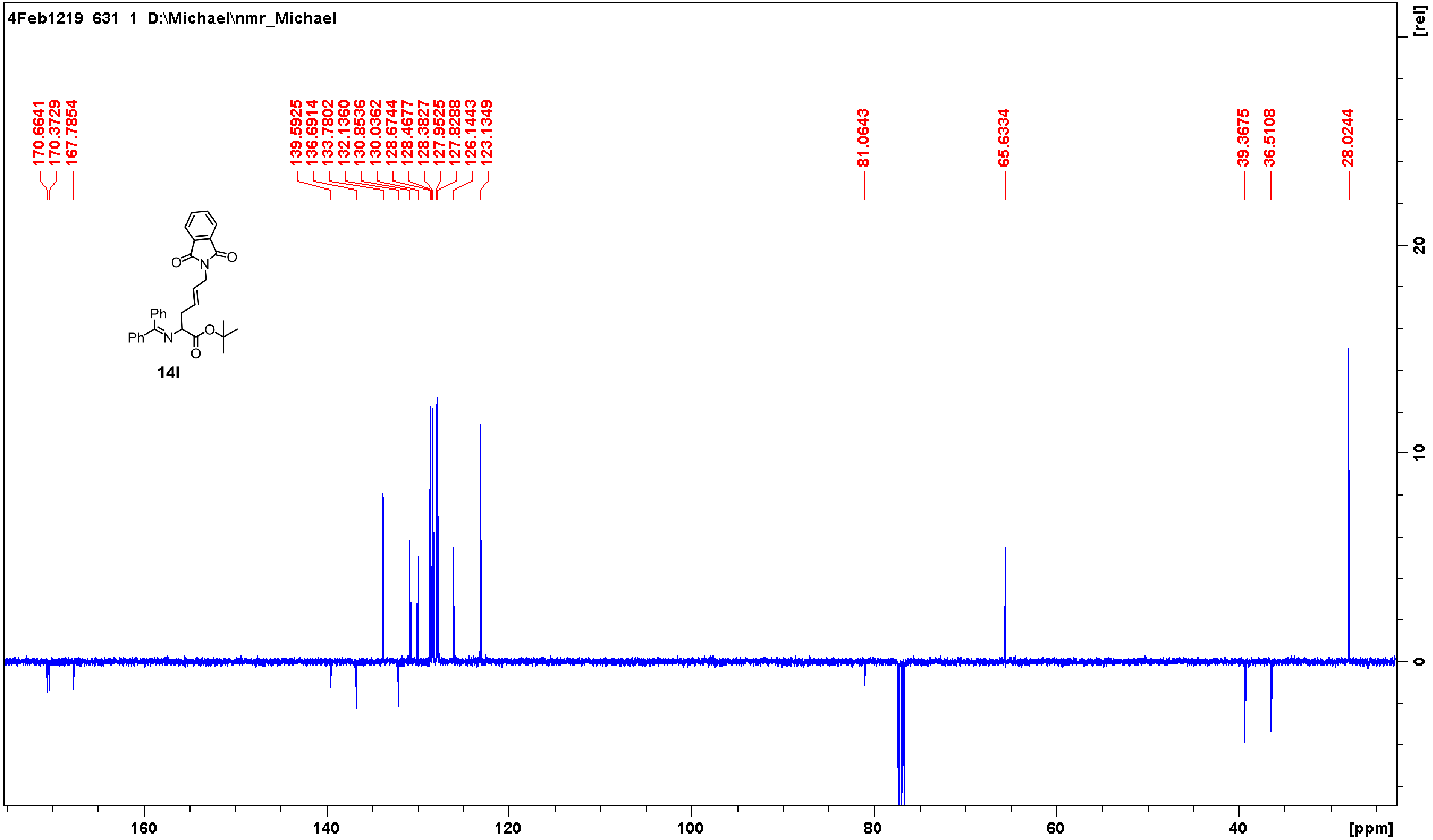
4Feb1219 631 1 D:\Michael\nmr_Michael
170.6641
170.3729
167.7854
139.5925
136.6914
133.7802
132.1360
130.8536
130.0362
128.6744
128.4677
128.3827
127.9525
127.8288
126.1443
123.1349
81.0643
65.6334
39.3675
36.5108
28.0244
Ph
Ph
N
O
O
N
O
O
14l
[rel]
20
10
0
160
140
120
100
80
60
40
[ppm]

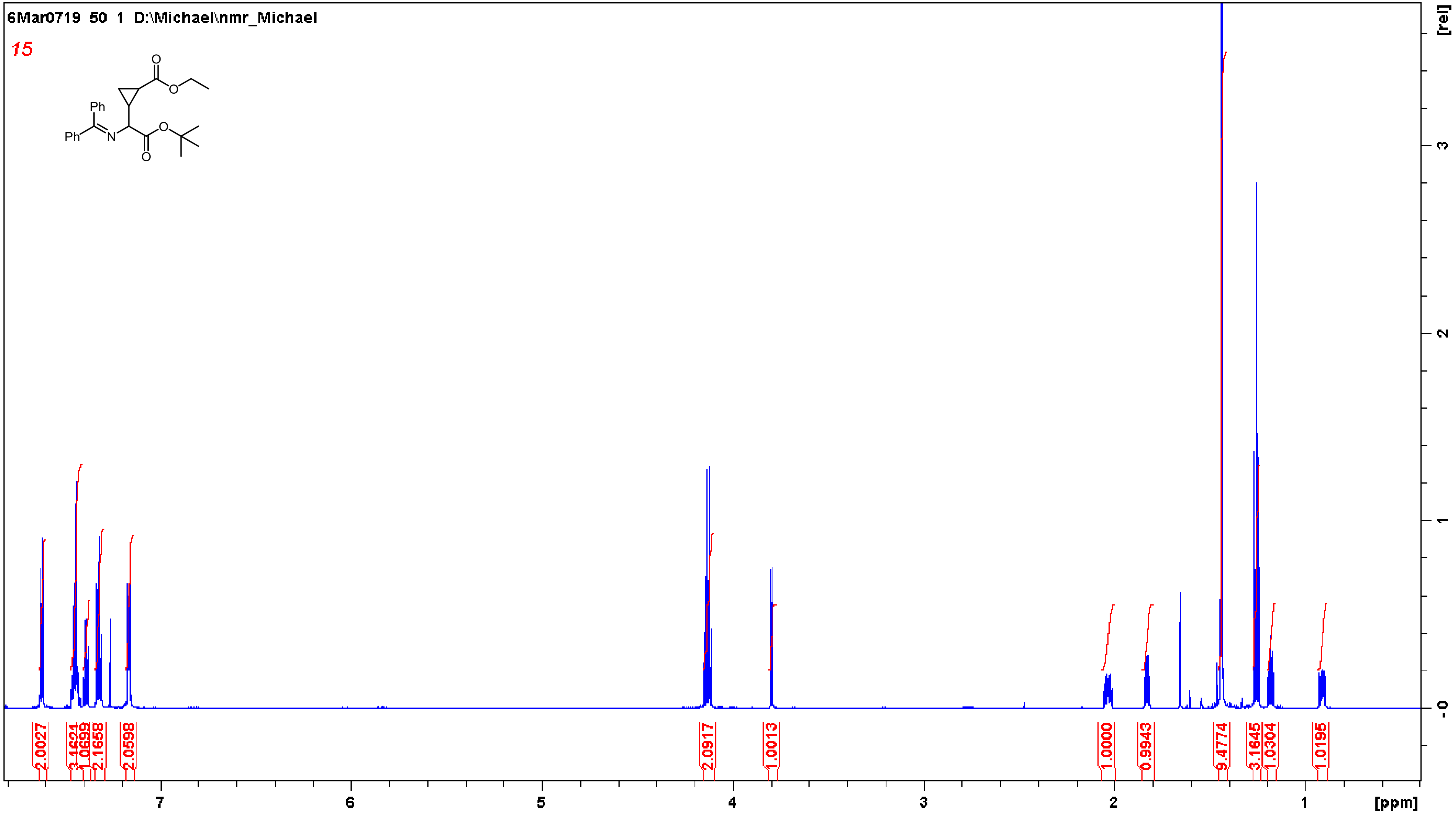
6Mar0719 50 1 D:\Michael\nmr_Michael
15
Ph
Ph
N
O
O
O
O
2.0027
3.1624
1.0699
2.1658
2.0598
2.0917
1.0013
1.0000
0.9943
9.4774
3.1645
1.0304
1.0195
7
6
5
4
3
2
1
[ppm]
[rel]
3
2
1
0

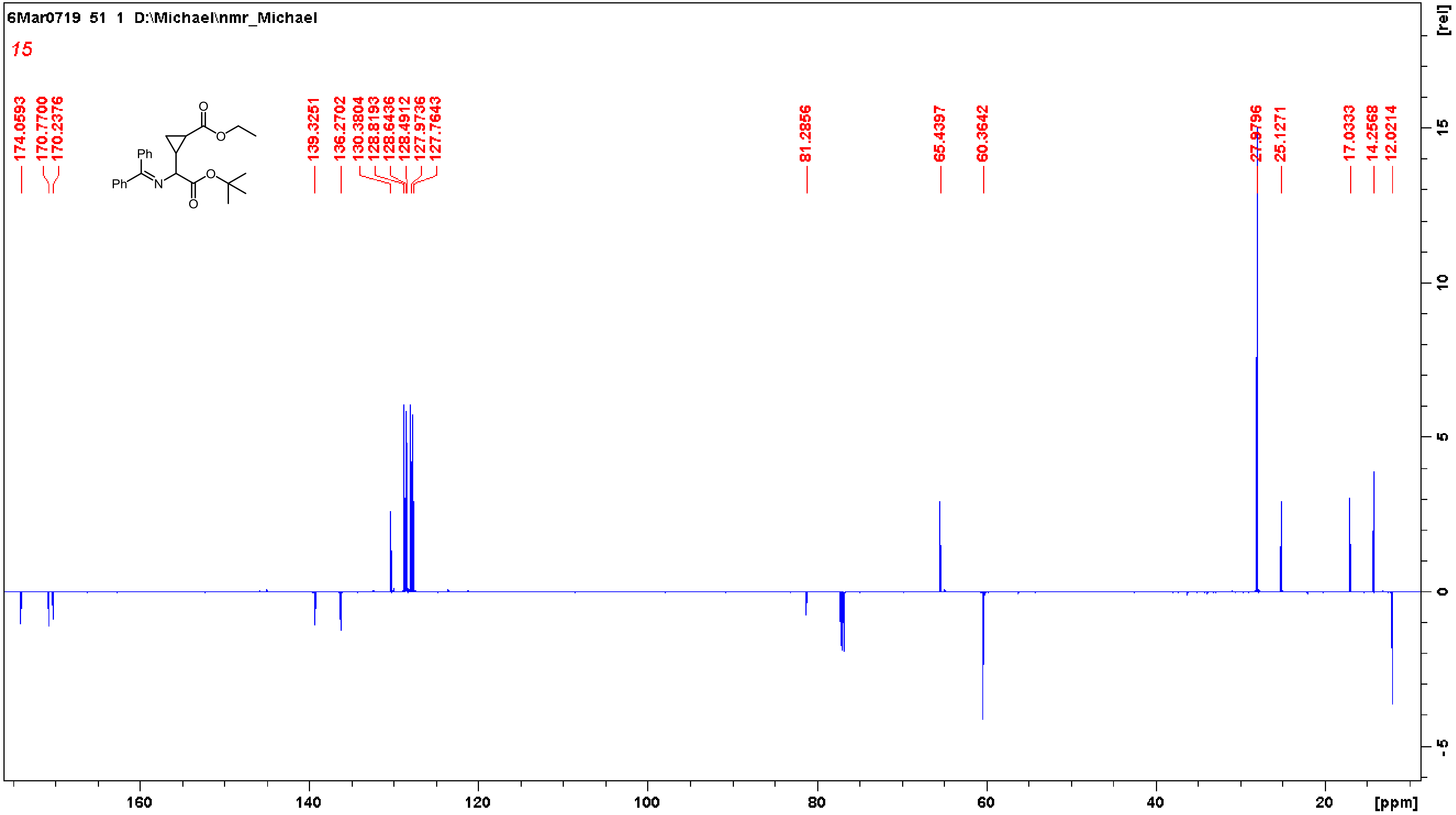
6Mar0719 51 1 D:\Michael\nmr_Michael
15
174.0593
170.7700
170.2376
139.3251
136.2702
130.3804
128.8193
128.6436
128.4912
127.9736
127.7643
81.2856
65.4397
60.3642
27.9796
25.1271
17.0333
14.2568
12.0214
Ph
Ph
N
O
O
O
O
[rel]
15
10
5
0
-5
160
140
120
100
80
60
40
20
[ppm]

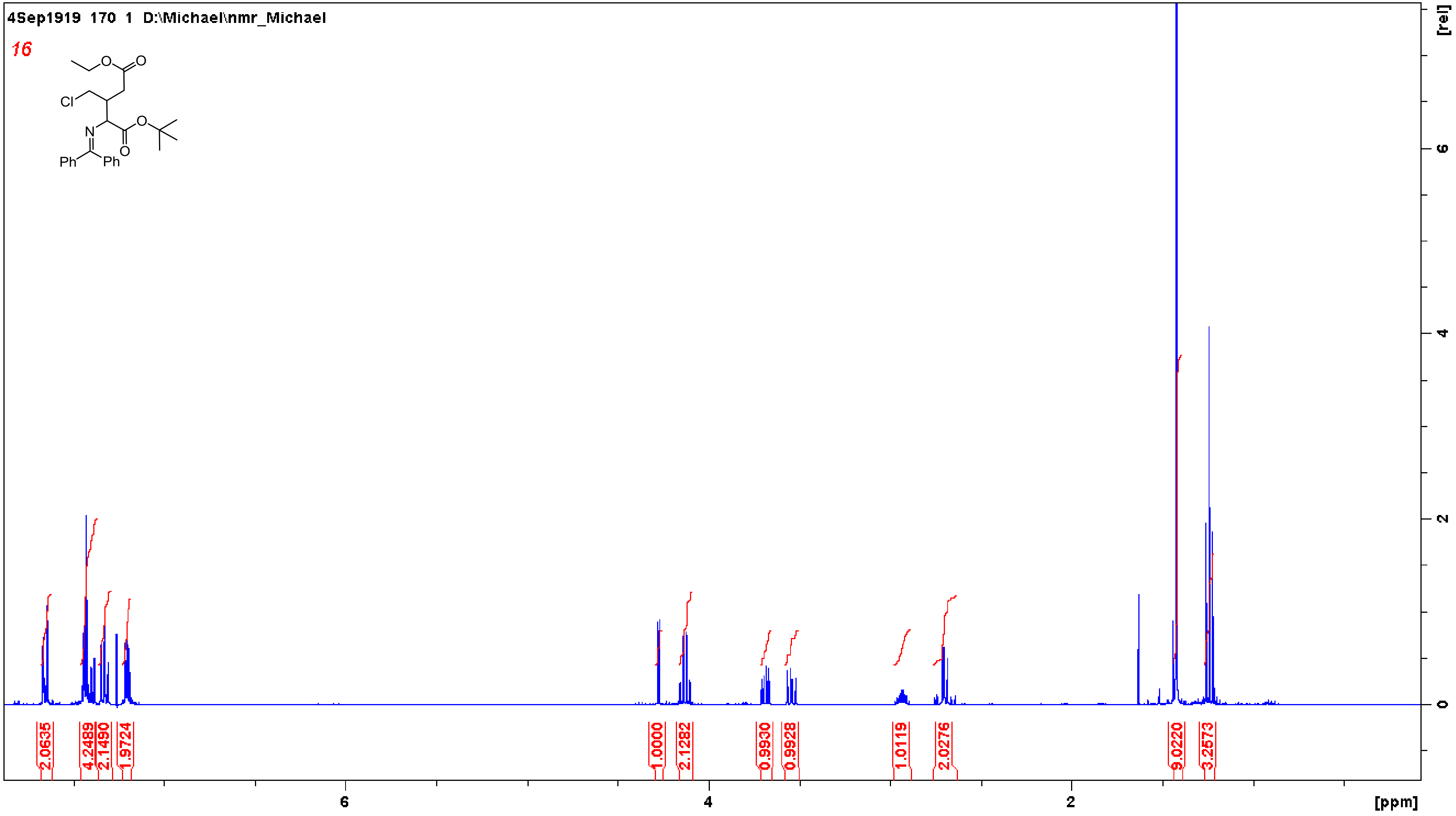
4Sep1919 170 1 D:\Michael\nmr_Michael
16
O
O
Cl
N
O
O
Ph
Ph
2.0635
4.2489
2.1490
1.9724
1.0000
2.1282
0.9930
0.9928
1.0119
2.0276
9.0220
3.2573
6
4
2
[ppm]
[rel]
0

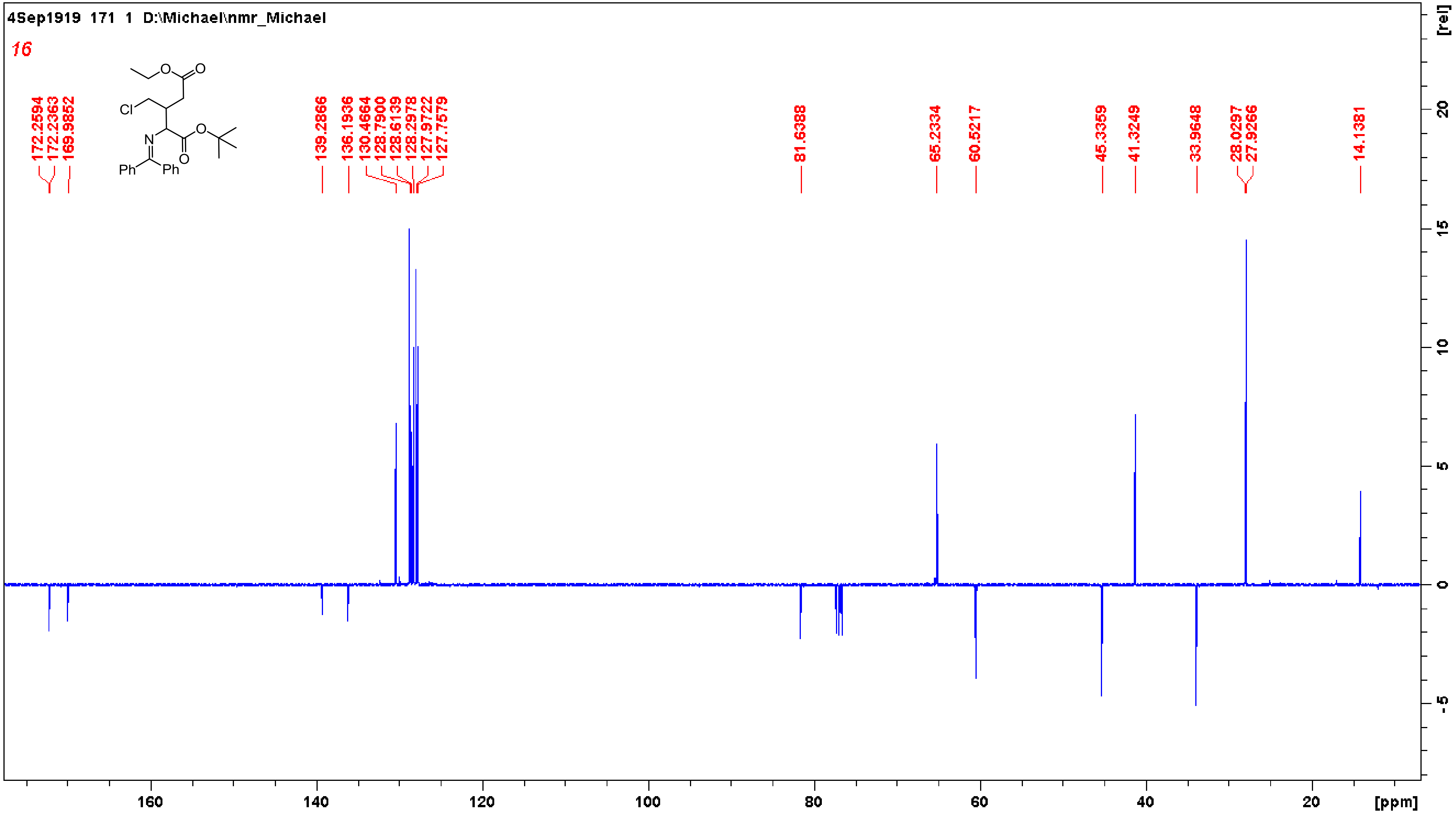
4Sep1919 171 1 D:\Michael\nmr_Michael
16
Cl
N
Ph
Ph
O
O
O
O
172.2594
172.2363
169.9852
139.2866
136.1936
130.4664
128.7900
128.6139
128.2978
127.9722
127.7579
81.6388
65.2334
60.5217
45.3359
41.3249
33.9648
28.0297
27.9266
14.1381
[rel]
20
15
10
5
0
-5
160
140
120
100
80
60
40
20
[ppm]

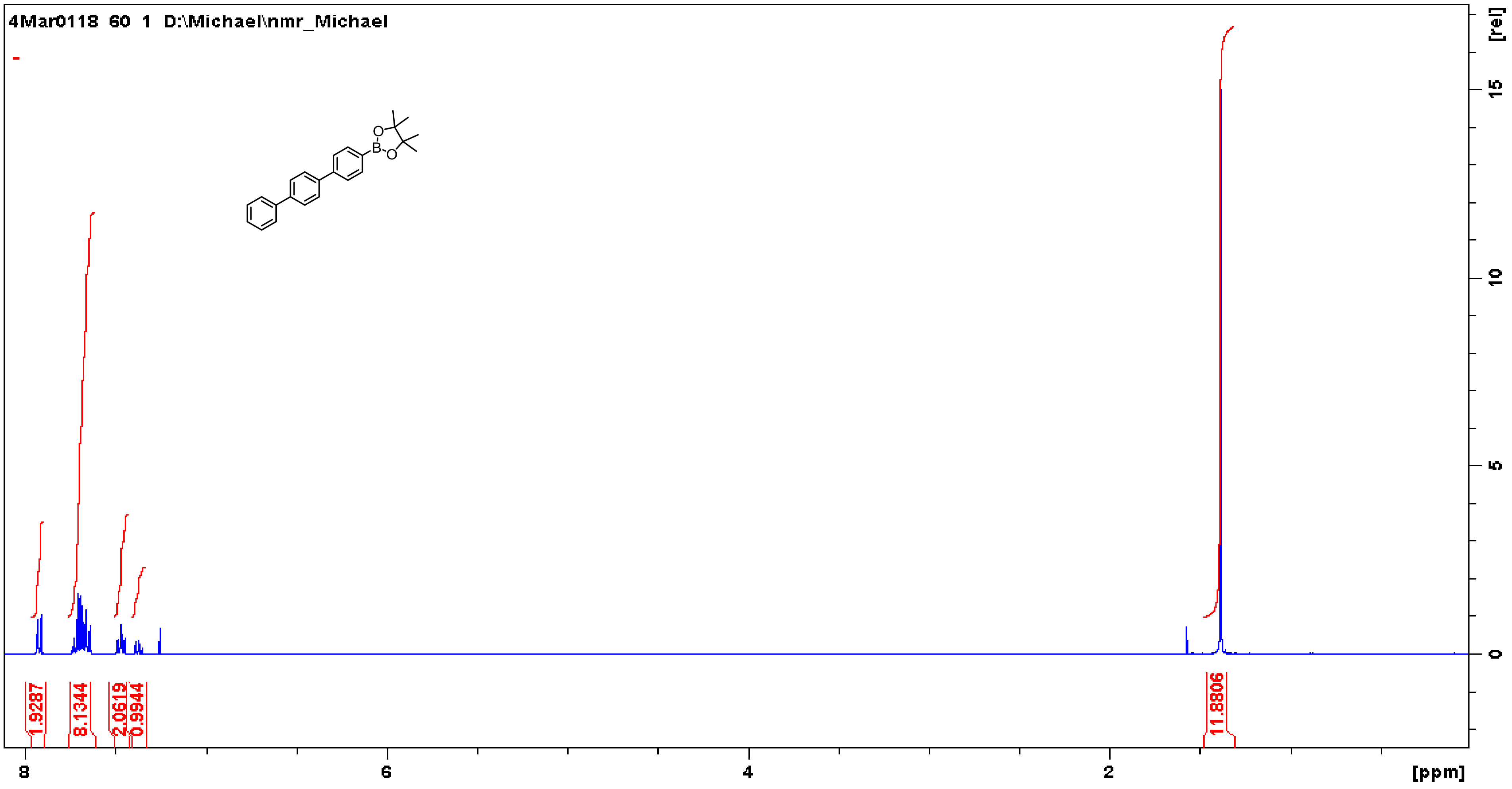
4Mar0118 60 1 D:\Michael\nmr_Michael
1.9287
8.1344
2.0619
0.9944
11.8806
[rel]
0
5
10
15
8
6
4
2
[ppm]
O
B
O

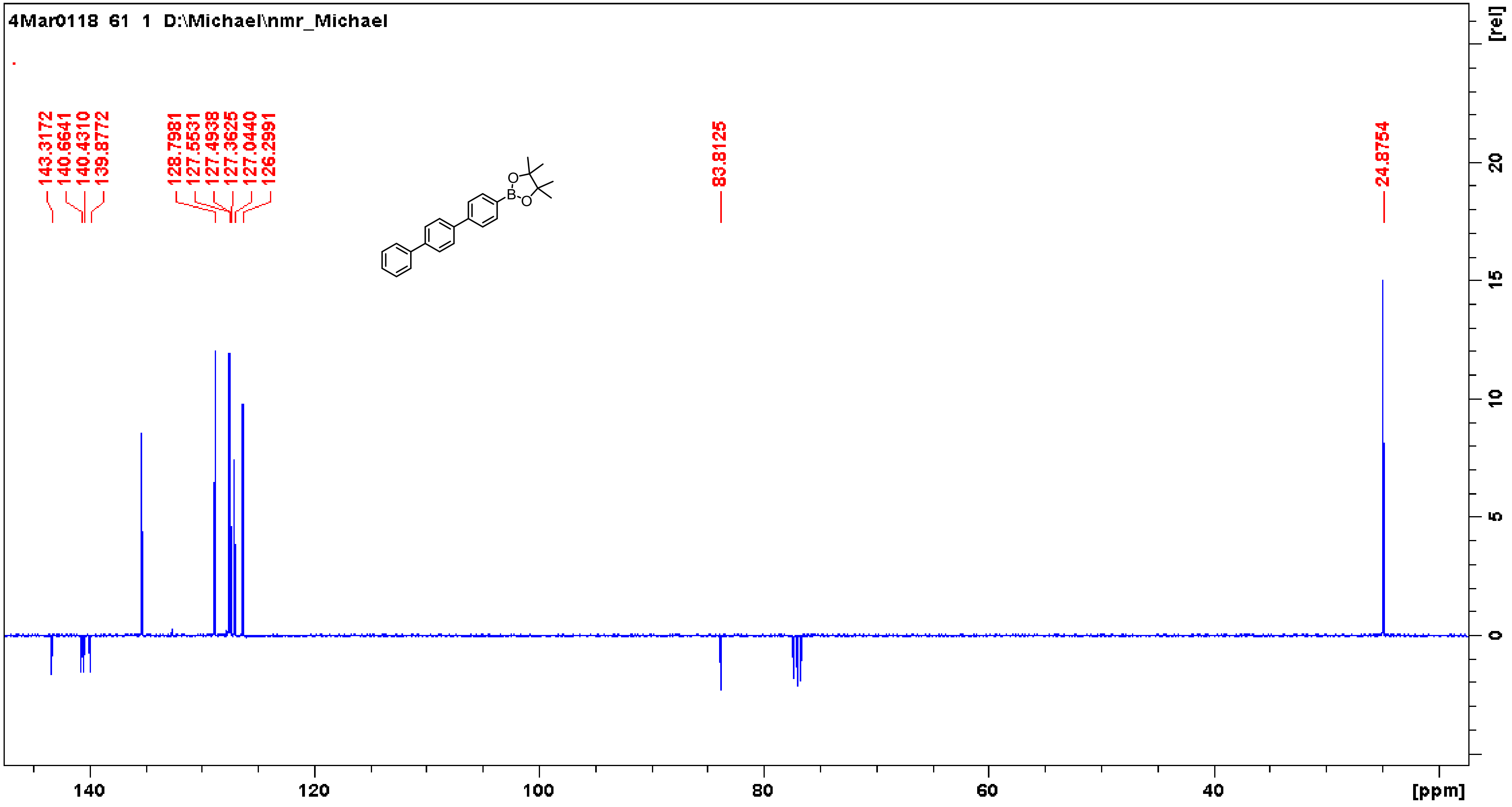
4Mar0118 61 1 D:\Michael\nmr_Michael
143.3172
140.6641
140.4310
139.8772
128.7981
127.5531
127.4938
127.3625
127.0440
126.2991
83.8125
24.8754
B
O
O
[rel]
20
15
10
5
0
140
120
100
80
60
40
[ppm]

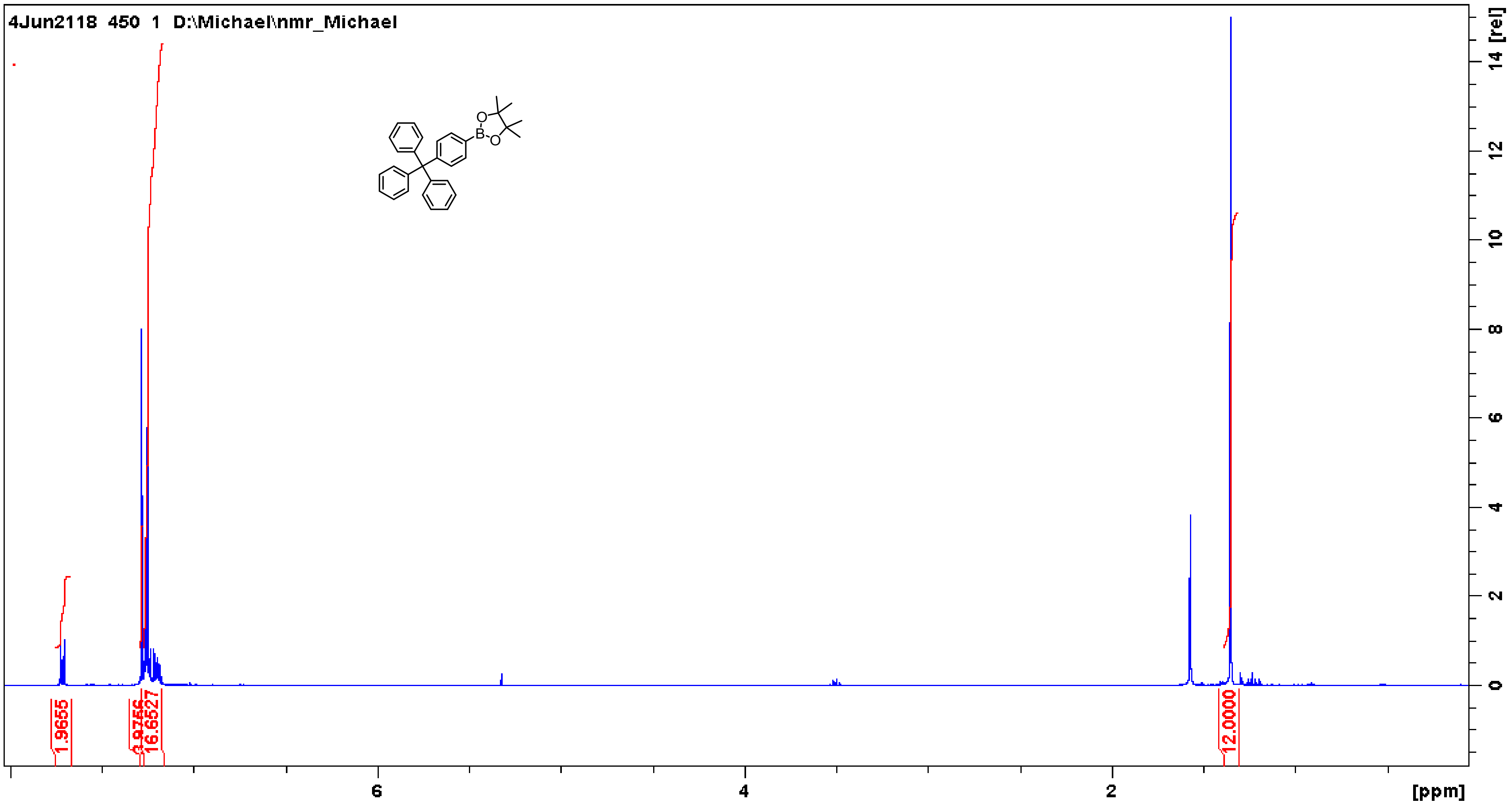

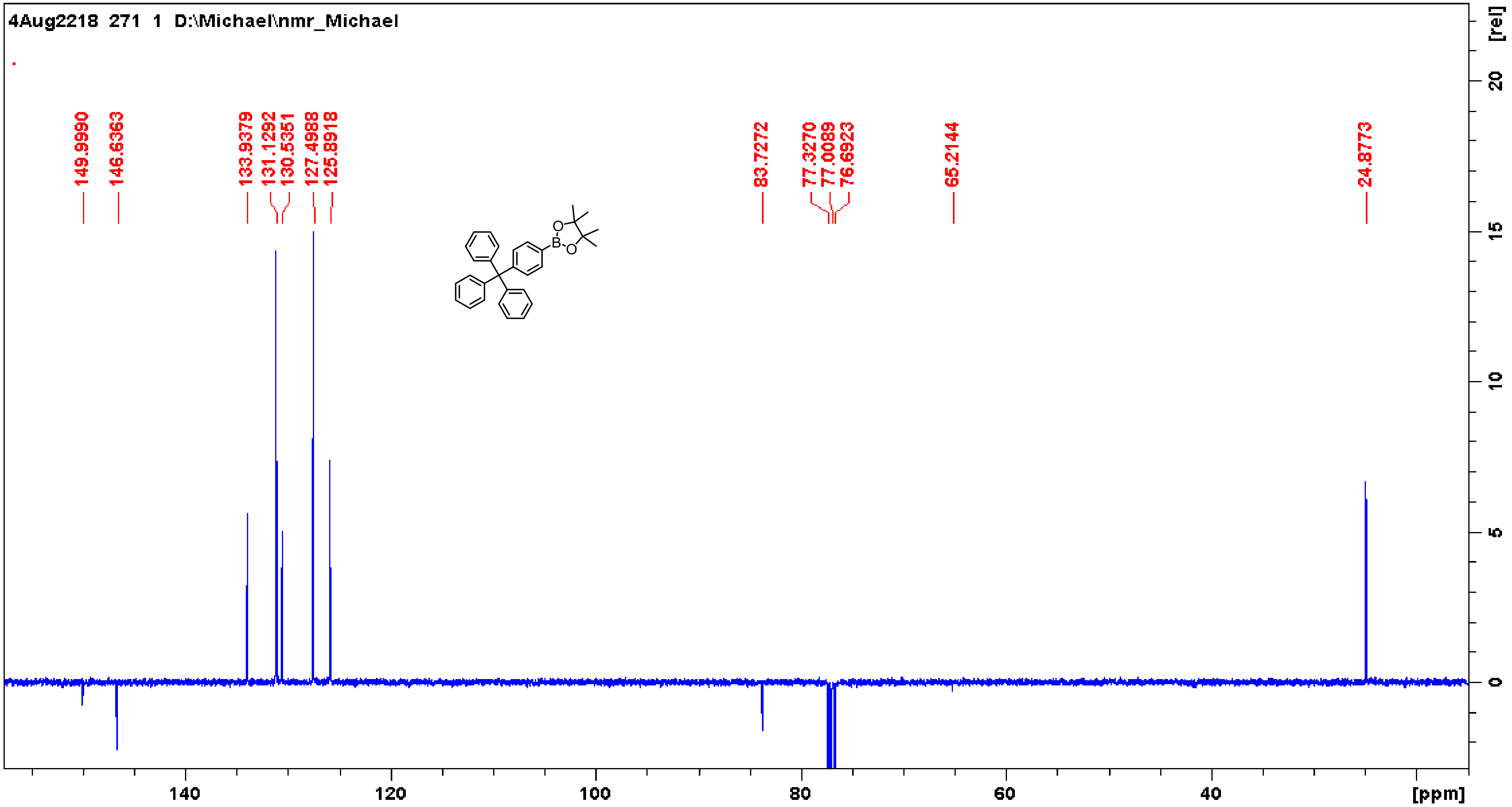
4Aug2218 271 1 D:\Michael\nmr_Michael
149.9990
146.6363
133.9379
131.1292
130.5351
127.4988
125.8918
83.7272
77.3270
77.0089
76.6923
65.2144
24.8773
O
B
O
[rel]
20
15
10
5
0
140
120
100
80
60
40
[ppm]

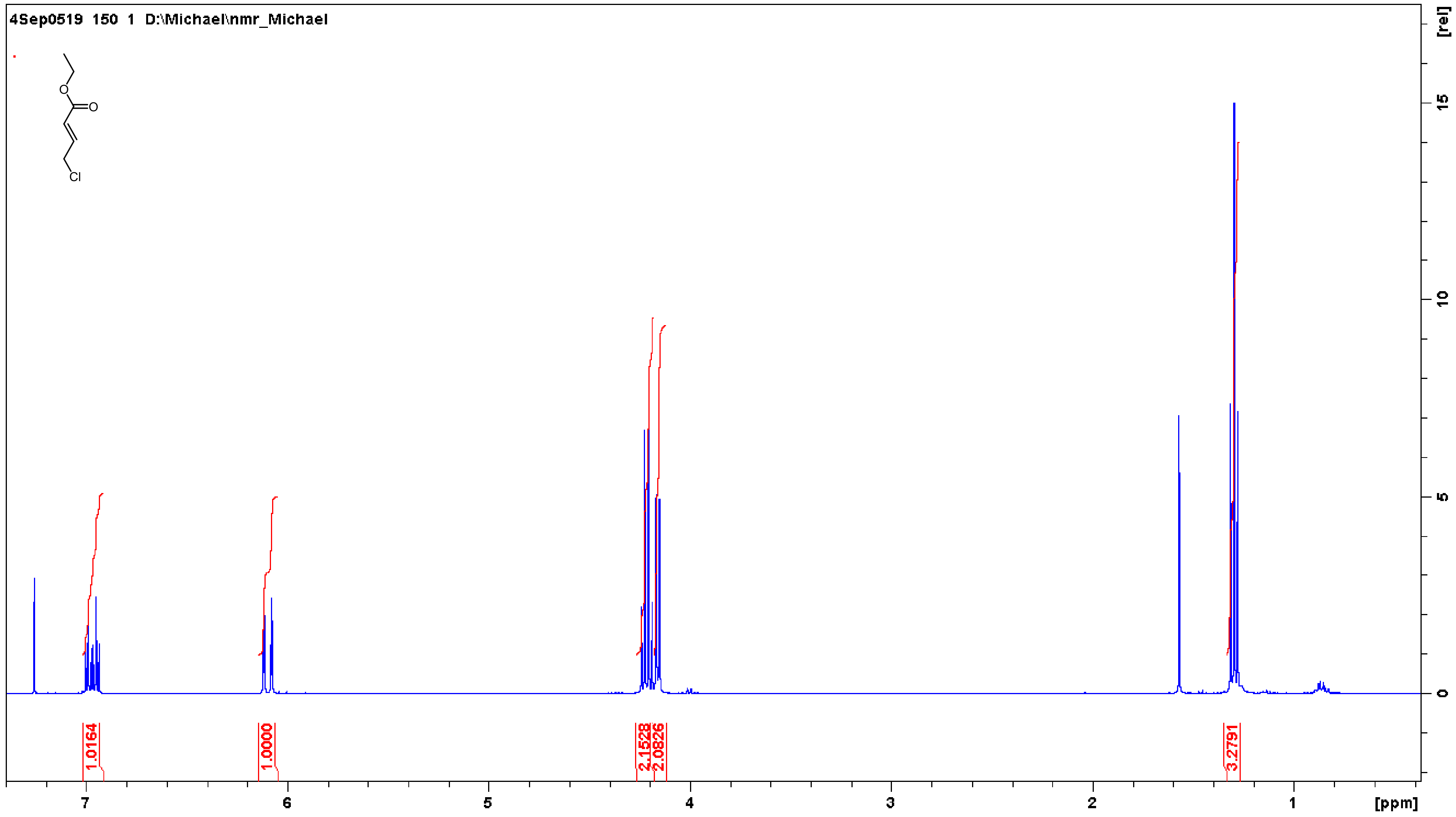
4Sep0519 150 1 D:\Michael\nmr_Michael
O
O
Cl
1.0164
1.0000
2.1528
2.0826
3.2791
7
6
5
4
3
2
1
[ppm]
[rel]
0
5
10
15

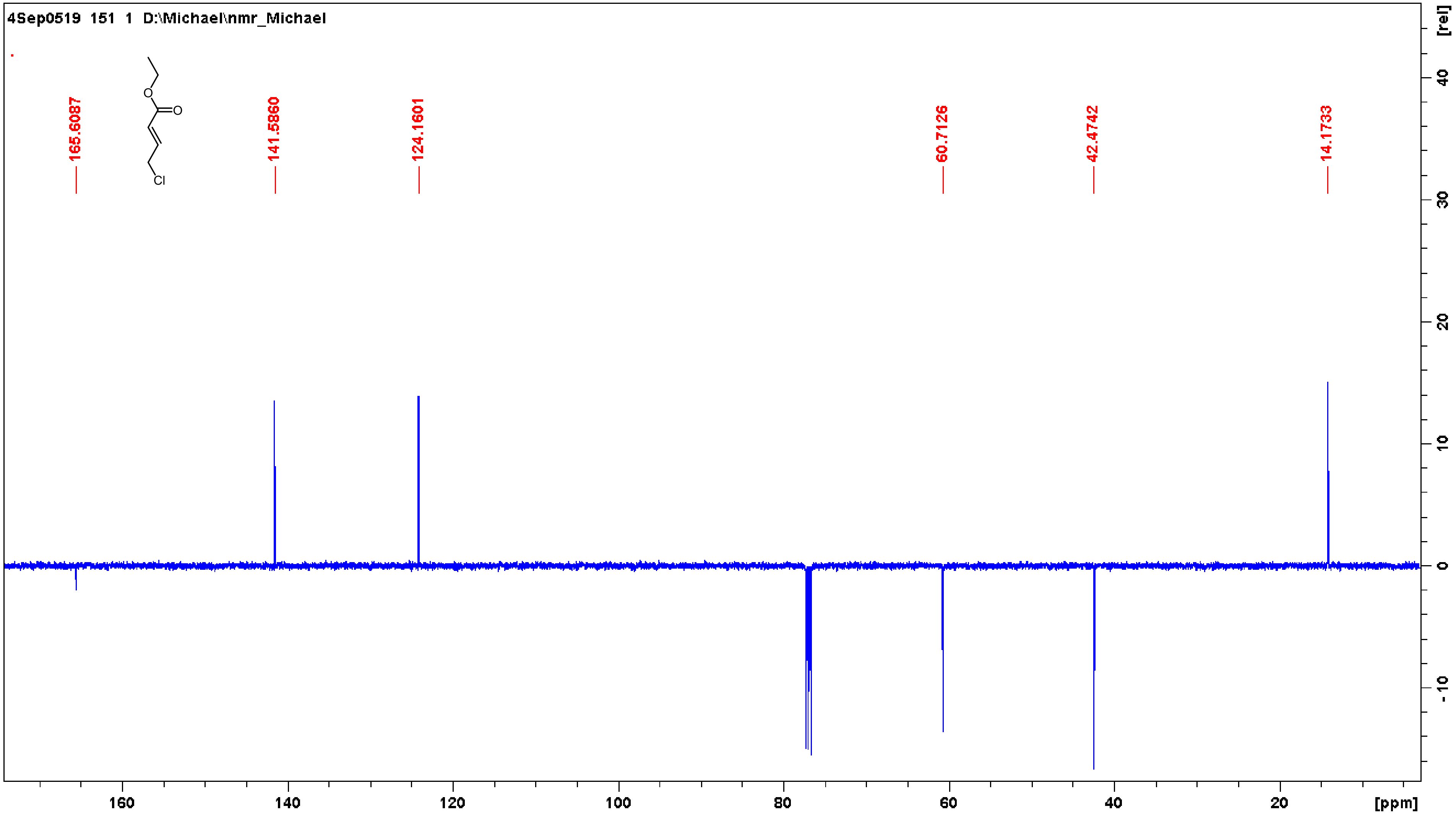
4Sep0519 151 1 D:\Michael\nmr_Michael
O
O
Cl
165.6087
141.5860
124.1601
60.7126
42.4742
14.1733
[rel]
40
30
20
10
0
-10
160
140
120
100
80
60
40
20
[ppm]

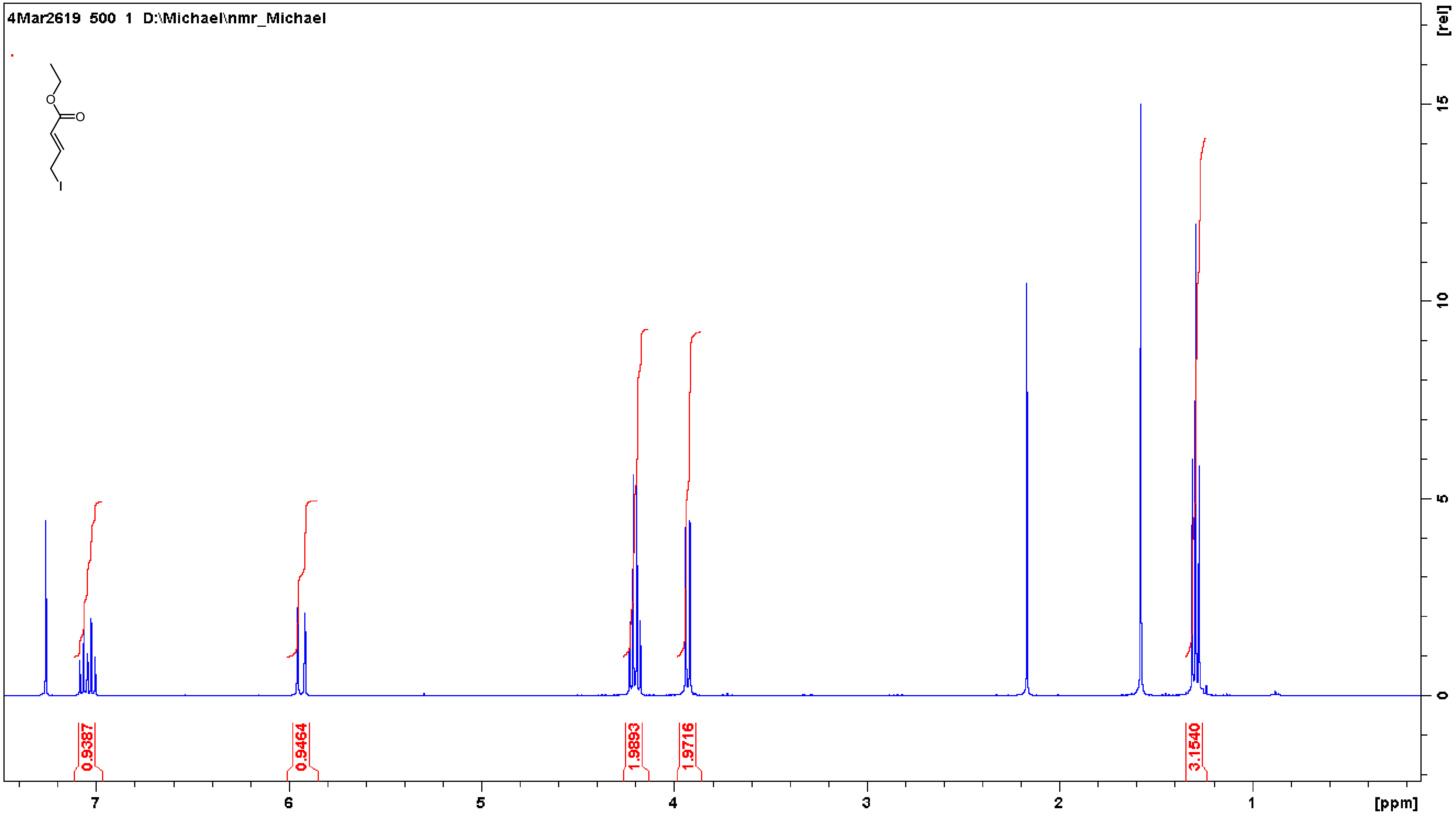
4Mar2619 500 1 D:\Michael\nmr_Michael
O
O
I
0.9387
0.9464
1.9893
1.9716
3.1540
7
6
5
4
3
2
1
[ppm]
[rel]
15
10
5
0

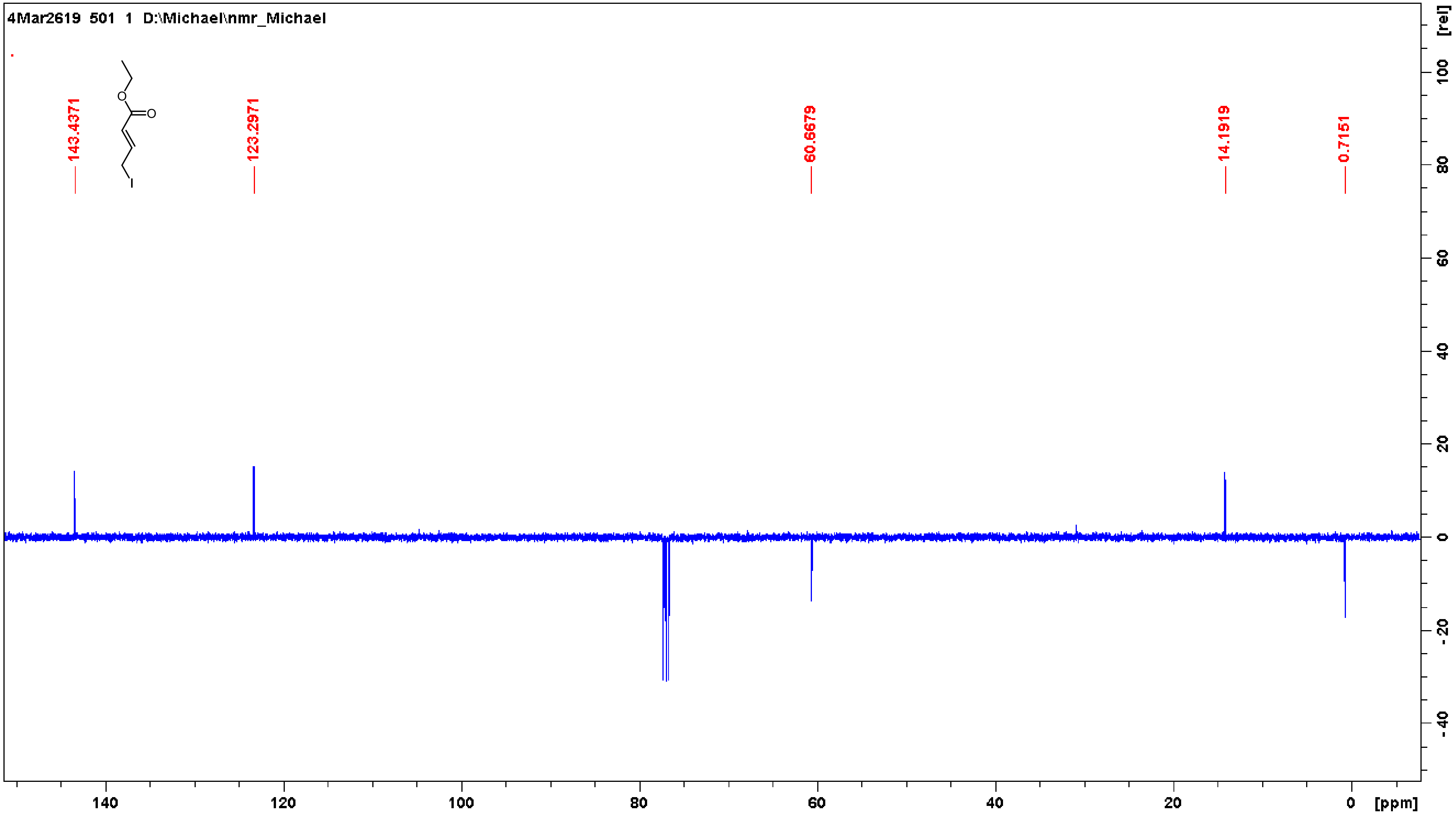
4Mar2619 501 1 D:\Michael\nmr_Michael
143.4371
123.2971
60.6679
14.1919
0.7151
[rel]
100
80
60
40
20
0
-20
-40
140
120
100
80
60
40
20
0
[ppm]
O
O
I

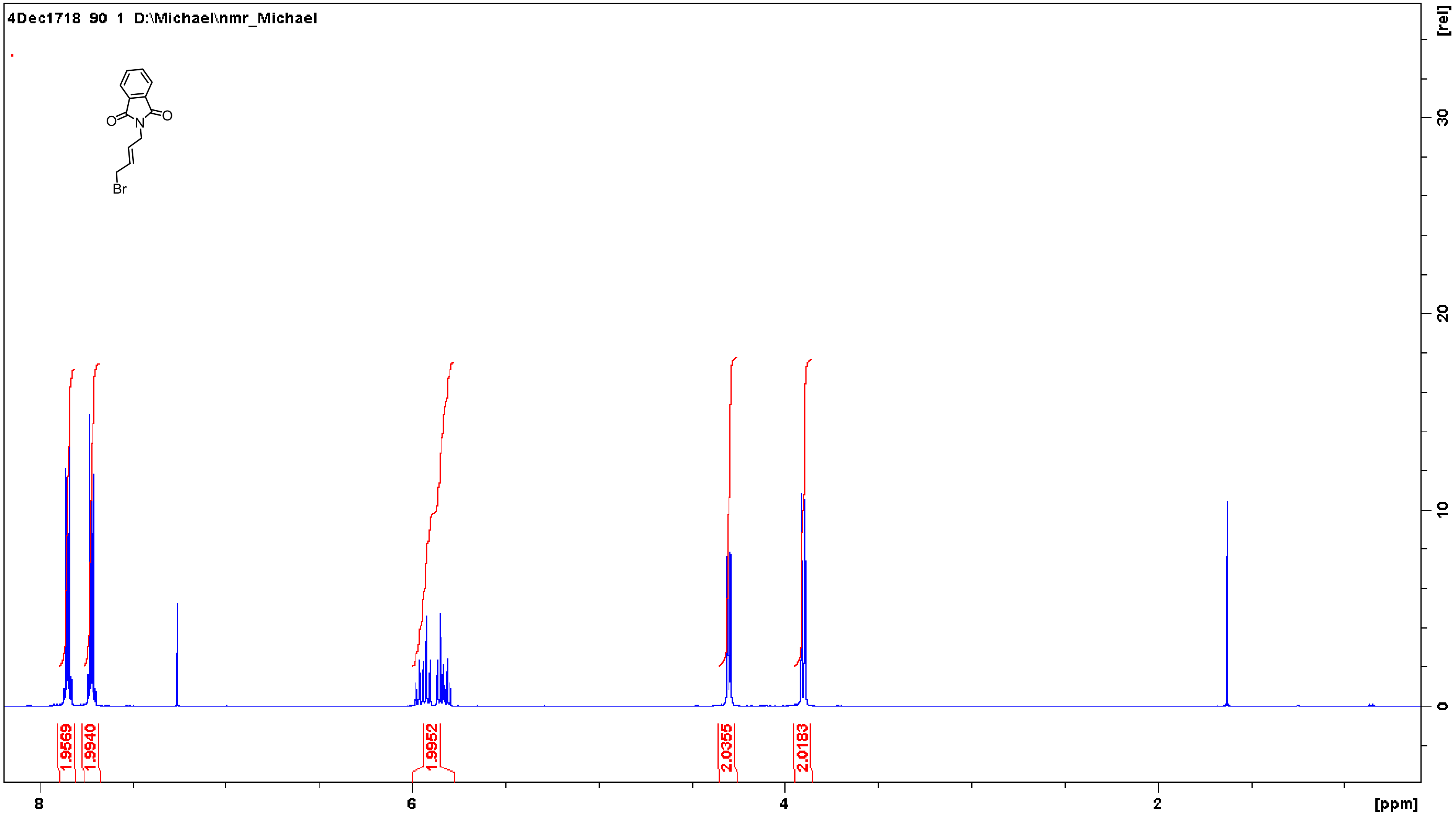
4Dec1718 90 1 D:\Michael\nmr_Michael
O
N
O
Br
1.9569
1.9940
1.9952
2.0355
2.0183
8
6
4
2
[ppm]
[rel]
30
20
10
0

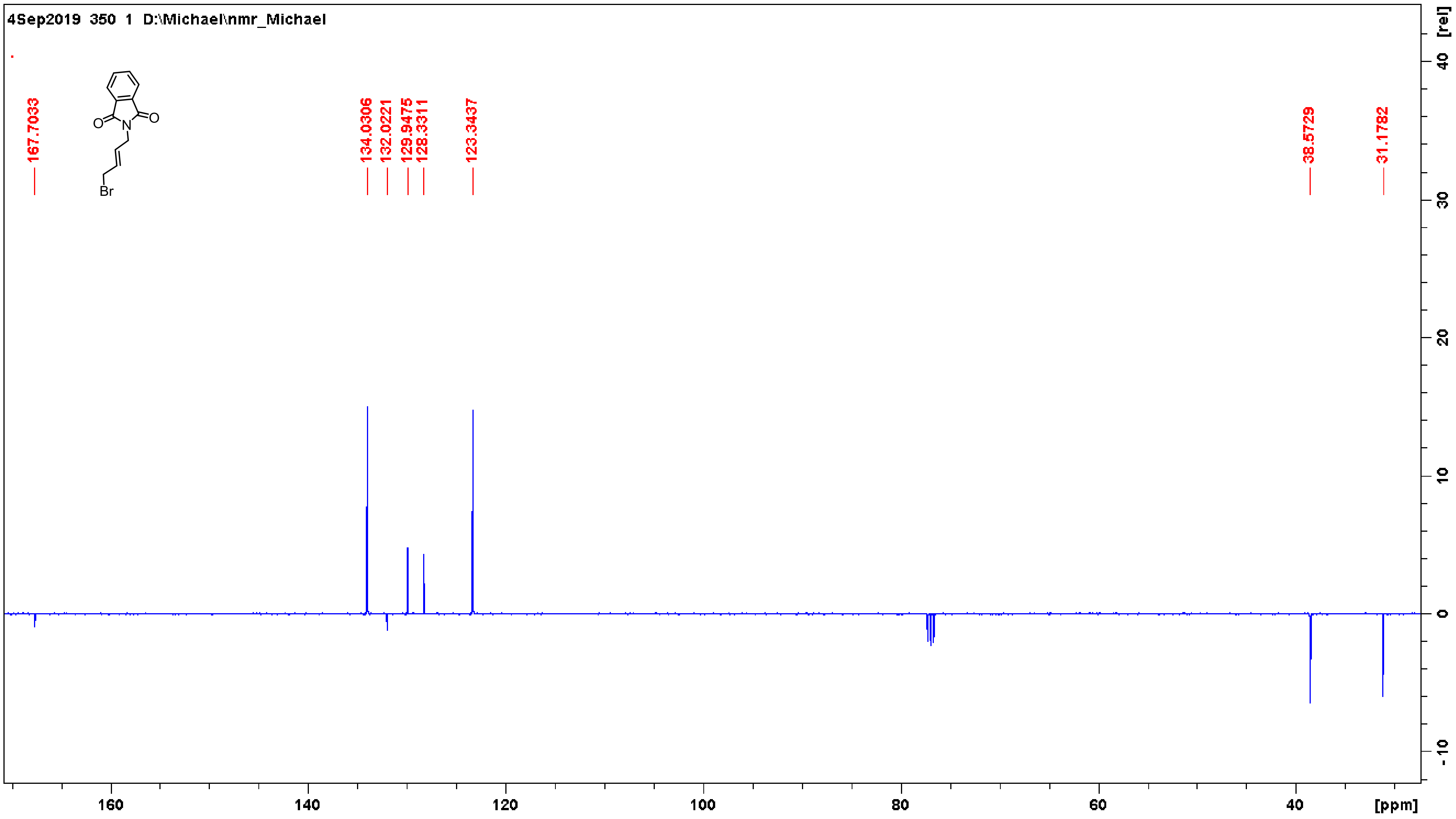

4Sep2019 350 1 D:\Michael\nmr_Michael
167.7033
134.0306
132.0221
129.9475
128.3311
123.3437
38.5729
31.1782
Br
N
O
O
160
140
120
100
80
60
40
[ppm]
40
30
20
10
0
-10
[rel]

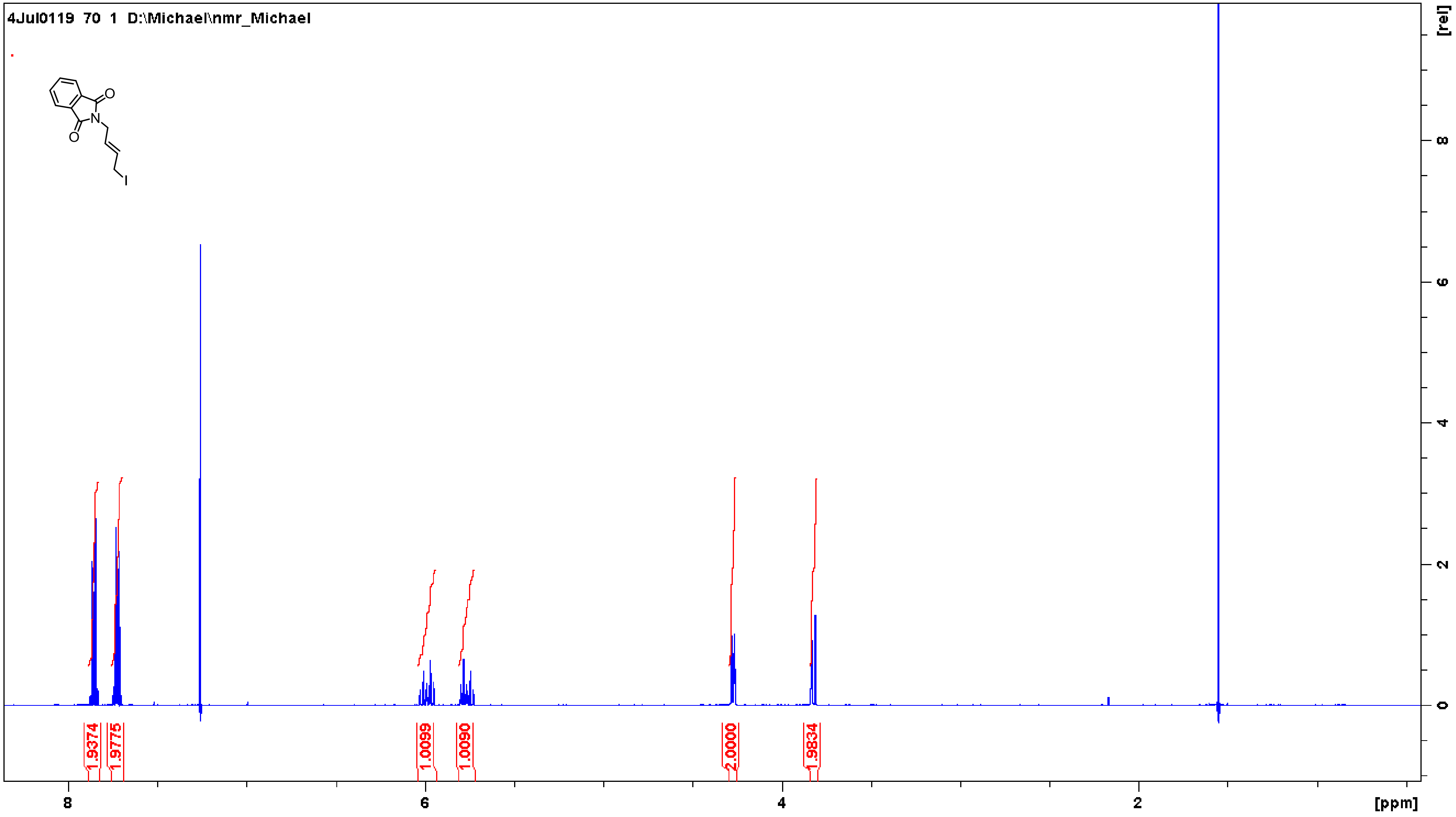
4Jul0119 70 1 D:\Michael\nmr_Michael
O
N
O
I
1.9374
1.9775
1.0099
1.0090
2.0000
1.9834
[rel]
[ppm]

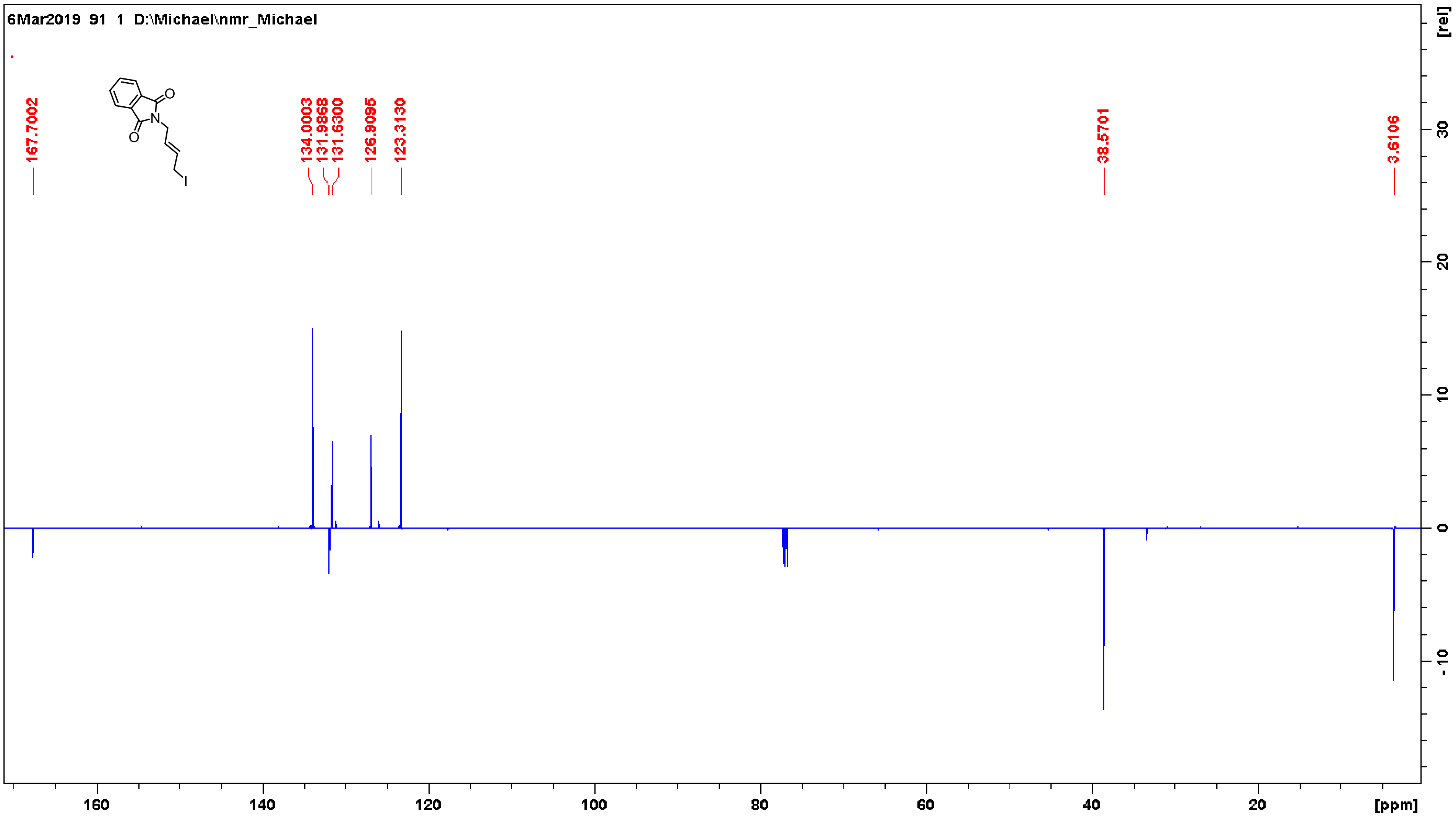

6Mar2019 91 1 D:\Michael\nmr_Michael
O
N
O
I
167.7002
134.0003
131.9868
131.6300
126.9095
123.3130
38.5701
3.6106
[rel]
30
20
10
0
-10
160
140
120
100
80
60
40
20
[ppm]

Comments on screening experiments in PTC – Practical aspects

For screening experiments it is essential to establish a standardized, reproducible procedure with minimum expenditure of time and resources. Typical key data of interest are conversion, regioselectivity, expressed in relative yields of regioisomers, formation of side and sequence products and dia- and enantioselectivity. Depending on complexity of the reaction mixture a column chromatography is usually performed to obtain "isolated yields". This step will give reproducible and reliable results if conducted on >100 mg scale but becomes increasingly error-prone on small scale. Moreover, this is the most time consuming part of the test reaction runs.

We therefore set out to develop a small scale screening procedure for our ligands, tested first in a prototypical reaction (**12** + **13A**, Scheme 2) which gives information on a) conversion (relativ to starting material), b) amount of product formed and isolated after extraction, and c) determination of enantiomeric excess, preferably without an extra step for purification or chromatographic separation. While a) can be often done by ^{1}H-NMR integration of proper, non-overlapping monitor protons, b) and c) might be more difficult to achieve.

We investigated 1,4-di(*tert*-butyl)benzene, 3,3'-dimethyl-1,1'-biphenyl and bibenzyl as internal standard (IS), added to the crude product mixture after extractive work-up, and recorded the ^{1}H-NMR spectrum in $CDCl_3$. Best results were obtained with bibenzyl. Integration of the well separated singlet of methylene protons (4H) relative to d×d of the methine (1H) or methylene group (2H) of the product gave %-conversion, amount of product formed and eventually side products (if extracted in the organic phase). After evaporation an aliquot was subjected to chiral HPLC measurement (ca. 5 mg dissolved in 1 mL of 2-PrOH/heptane, 5 µL injected). For reaction (1) using reported conditions (Chiralcel ODH, 250 × 4.6 mm, 2-PrOH/heptane, 1:99, 0.5 mL min^{-1}, 25 °C) well resolved peaks in the order of elution of internal standard, (*R*)-product, benzophenone, starting material, (*S*)-product were obtained.

Screening experiments (Details):

Table S1. Asymmetric substitution of *tert*-butyl 2-((diphenylmethylene)amino)acetate (**12**) with electrophiles **13B-13I** under PT conditions.[a]

entry	cat.	t/h	base	product	yield/%[b]	ee/%[c]	note
1	**1c**	20	KOH	**14B**	89	88	
2	**1d**	20	KOH	**14B**	**90**	88	
3	**1g**	20	KOH	**14B**	89	**98**	d
4	**1h**	20	KOH	**14B**	70	**98**	
5	**1c**	20	KOH	**14C**	80	91	
6	**1d**	20	KOH	**14C**	**86**	91	
7	**1g**	20	KOH	**14C**	71	**95**	e
8	**1h**	20	KOH	**14C**	45	52	
9	**1c**	20	KOH	**14D**	**90**	75	
10	**1d**	20	KOH	**14D**	**90**	74	
11	**1g**	20	KOH	**14D**	87	**93**	
12	**1h**	20	KOH	**14D**	61	84	

entry	cat.	t/h	base	product	yield/%[b]	ee/%[c]	note
13	**1c**	20	KOH	**14E**	51	83	
14	**1d**	20	KOH	**14E**	55	**88**	
15	**1g**	20	KOH	**14E**	**72**	68	
16	**1h**	20	KOH	**14E**	66	14	
17	**1c**	20	KOH	**14F**	**92**	89	
18	**1d**	20	KOH	**14F**	91	88	
19	**1g**	20	KOH	**14F**	90	**90**	f
20	**1h**	20	KOH	**14F**	30	90	
21	**1c**	20	KOH	**14G**	88	72	
22	**1d**	20	KOH	**14G**	**91**	71	
23	**1g**	20	KOH	**14G**	81	**83**	
24	**1h**	20	KOH	**14G**	83	73	
25	**1c**	20	CsOH	**14H**	62	51	g
26	**1d**	20	CsOH	**14H**	56	41	h
27	**1g**	4	KOH	**14H**	13	69	i
28	**1g**	4	KOH	**14H**	39	62	j
29	**1g**	4	CsOH	**14H**	54	74	
30	**1g**	20	CsOH	**14H**	**61**	79	k
31	**1h**	20	CsOH	**14H**	60	**80**	
32	**1c**	20	CsOH	**14H**	22	35	l
33	**1d**	20	CsOH	**14H**	<10	n.d.	l
34	**1g**	20	CsOH	**14H**	52	68	l
35	**1h**	20	CsOH	**14H**	51	67	l
36	**1g**	20	CsOH	**14H**	0	n.d.	m
37	**1c**	20	Cs_2CO_3	**14I**	<5	n.d.	
38	**1g**	20	Cs_2CO_3	**14I**	33	**88**	
39	**1h**	20	Cs_2CO_3	**14I**	<5	n.d.	
40	**1c**	20	Cs_2CO_3	**14I**	**33**	57	n
41	**1d**	20	Cs_2CO_3	**14I**	26	51	n
42	**1g**	20	Cs_2CO_3	**14I**	23	83	n
43	**1h**	20	Cs_2CO_3	**14I**	20	83	n

[a] 0.25 mmol of substrate, 0.30 mmol of electrophile, 1 mol % of catalyst with (*S,S*)-configuration, 1.5 mL toluene, 0.5 mL KOH or CsOH (50%) or solid Cs_2CO_3 (5 equiv.), vigorous stirring at 0 °C for the time indicated. [b] Yield after extractive work-up based on ^{1}H-NMR integration of signals of product and bibenzyl added as internal standard. [c] Determined by chiral HPLC (Chiralcel ODH or Chiralpak ADH), products with (*R*)-configuration predominating. [d] 99% ee, 80% isolated yield, 0 °C, 24 h [1]. [e] 99%ee 86% over two steps, 0 °C, 24 h [1]. [f] 99% ee 89% isolated yield, 0 °C, 15 h [1]. [g] 12% of **15** formed. [h] 13% of **15** formed. [i] 37% of **15** formed. [j] 10 equ. of KI added, 27% of **15** formed. [k] 8% of **15** formed. [l] Instead of **13H** the corresponding 4-iodocrotonate was used. [m] Instead of **13H** the corresponding 4-chlorocrotonate was used, resulting in formation of 53% of **15** and 27% of intermediate **16** (see structure below). [n] Instead of **13I** the corresponding iodo compound was used.

1-(tert-Butyl) 5-ethyl 3-(chloromethyl)-2-((diphenylmethylene)amino)pentanedioate (**16**): ^{1}H-NMR δ: 7.63–7.67 (m, 2H); 7.37–7.45 (m, 4H); 7.30–7.35 (m, 2H); 7.18–7.22 (m, 2H); 4.18 (d, *J* = 4.1 Hz, 1H); 4.12 (qm, *J* = 7.1 Hz, 2H); 3.69 (dd, *J* = 11.0, 5.0 Hz, 1H); 3.54 (dd, *J* = 10.9, 8.2 Hz, 1H); 2.90–2.97 (m, 1H); 2.73 (dd, *J* = 16.5, 4.8 Hz, 1H); 2.67 (dd, *J* = 16.5, 8.5 Hz, 1H); 1.42 (s, 9H); 1.24 (t, *J* = 7.1 Hz, 3H) ppm. ^{13}C-NMR δ: 172.26 (C); 172.24 (C); 169.99 (C); 139.29 (C); 136.19 (C); 130.47 (CH); 128.79 (CH); 128.61 (CH); 128.30 (CH); 127.97 (CH); 127.76 (CH); 81.64 (C); 65.23 (CH); 60.52 (CH_2); 45.34 (CH_2); 41.32 (CH); 33.96 (CH_2); 27.93 (CH_3); 14.14 (CH_3) ppm. HRMS (ESI) calcd for $C_{25}H_{30}ClNaNO_4$ $[M + Na]^+$: 466.1761; found. 466.1746.

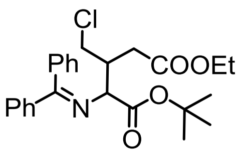

16

Ethyl (E)-4-chlorobut-2-enoate: To a stirred solution of ethyl 4-bromocrotonate (772 mg, 3.0 mmol 75% purity) in DMF (1 mL) was added dry LiCl (636 mg, 5 equiv.) at room temperature. After 24 h, the suspension was poured into water/ether (10 mL/30 mL) and the organic phase was washed with water (4 × 10 mL) and dried ($MgSO_4$). The crude product was purified by chromatography (EtOAc (0→15%)/heptane) to afford the product as a colorless oil; yield 80–90%. ^{1}H-NMR δ: 6.97 (dt, *J* = 15.4, 6.1 Hz, 1H); 6.10 (dt, *J* = 15.3, 1.6 Hz, 1H); 4.22 (q, *J* = 7.1 Hz, 2H); 4.16 (dd, *J* = 6.1, 1.6 Hz, 2H); 1.30 (t, *J* = 7.1 Hz, 3H) ppm. ^{13}C-NMR δ: 165.61 (C); 141.59 (CH); 124.16 (CH); 60.71 (CH_2); 42.47 (CH_2); 14.17 (CH_3) ppm. HRMS (ESI) calculated for C_4H_4ClO $[M - OC_2H_5]^+$: 102.9951, found 102.9941.

UV traces of chiral HPLC separations

Sample Name: W STD 14

```
=====================================================================
Acq. Operator   : SYSTEM                          Seq. Line :    1
Acq. Instrument : LC1200                           Location :    1
Injection Date  : 04.09.2018 18:39:02                   Inj :    1
                                                Inj Volume : 5.000 µl
Method          : C:\Chem32\1\Data\MW\MW-1 2018-09-04 18-37-58\CHIR_ODH.M (Sequence Method)
Last changed    : 04.09.2018 18:37:59 by SYSTEM
Sample Info     : ODH, 1% iPrOH, 25°C, 0.5 mL min-1

Additional Info : Peak(s) manually integrated
```

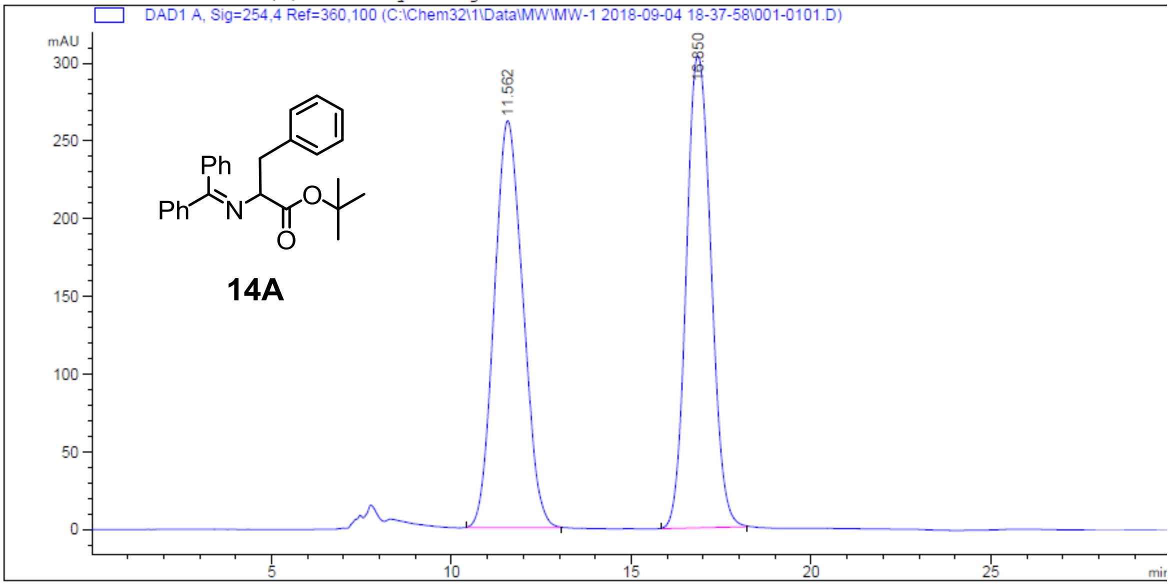


```
=====================================================================
                         Area Percent Report
=====================================================================

Sorted By             :      Signal
Multiplier            :      1.0000
Dilution              :      1.0000
Use Multiplier & Dilution Factor with ISTDs


Signal 1: DAD1 A, Sig=254,4 Ref=360,100

Peak RetTime Type  Width     Area      Height     Area
  #   [min]        [min]   [mAU*s]     [mAU]        %
----|-------|----|-------|----------|----------|--------|
   1  11.562 BB    0.8486 1.45102e4  261.82501  50.0590
   2  16.850 BB    0.7523 1.44760e4  303.57013  49.9410

Totals :                  2.89863e4  565.39514
```

Sample Name: STD-D1

```
=====================================================================
Acq. Operator   : SYSTEM                          Seq. Line :    3
Acq. Instrument : LC1200                           Location :    3
Injection Date  : 25.10.2018 17:48:21                   Inj :    1
                                                 Inj Volume : 5.000 µl
Acq. Method     : C:\Chem32\1\Data\MW\MW-2 2018-10-25 16-41-46\CHIR_ADH.M
Last changed    : 25.10.2018 17:46:20 by SYSTEM
Analysis Method : C:\Chem32\1\Data\MW\MW-2 2018-10-25 16-41-46\CHIR_ADH.M (Sequence Method)
Last changed    : 25.10.2018 17:47:16 by SYSTEM
Sample Info     : W STD-D1  Chiralpak ADH 2-PrOH/heptane  (0.5:99.5) 0.5 mL min-1, 25 °C

Additional Info : Peak(s) manually integrated
```

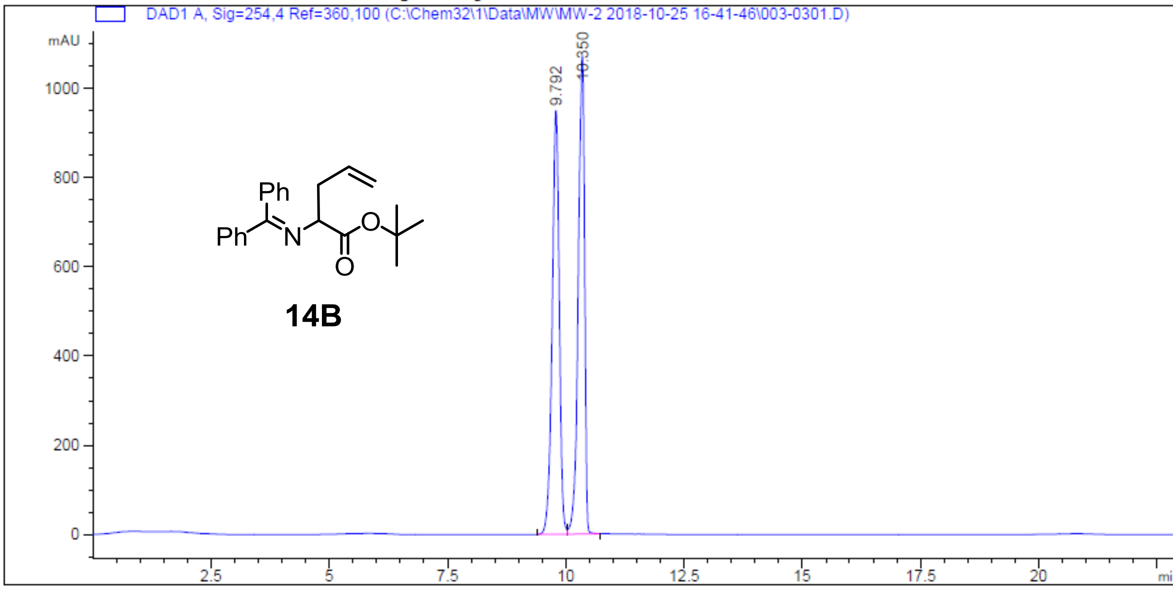


```
=====================================================================
                          Area Percent Report
=====================================================================

Sorted By             :      Signal
Multiplier            :      1.0000
Dilution              :      1.0000
Do not use Multiplier & Dilution Factor with ISTDs


Signal 1: DAD1 A, Sig=254,4 Ref=360,100
```

Peak #	RetTime [min]	Type	Width [min]	Area [mAU*s]	Height [mAU]	Area %
1	9.792	BV	0.1596	9905.24414	949.96033	49.8037
2	10.350	VB	0.1506	9983.34277	1072.17236	50.1963

Totals : 1.98886e4 2022.13269

Sample Name: STD-C1

```
=====================================================================
Acq. Operator   : SYSTEM                          Seq. Line :   2
Acq. Instrument : LC1200                           Location :   2
Injection Date  : 25.10.2018 15:01:57                   Inj :   1
                                                 Inj Volume : 5.000 µl
Acq. Method     : C:\Chem32\1\Data\MW\MW-2 2018-10-25 13-59-36\CHIR_ADH.M
Last changed    : 25.10.2018 15:44:49 by SYSTEM
                  (modified after loading)
Analysis Method : C:\Chem32\1\Data\MW\MW-2 2018-10-25 13-59-36\CHIR_ADH.M (Sequence Method)
Last changed    : 20.08.2019 17:30:28 by SYSTEM
                  (modified after loading)
Sample Info     : W STD-C1  Chiralpak ADH 2-PrOH/heptane (1:99) 0.5 mL min-1, 25 °C

Additional Info : Peak(s) manually integrated
```

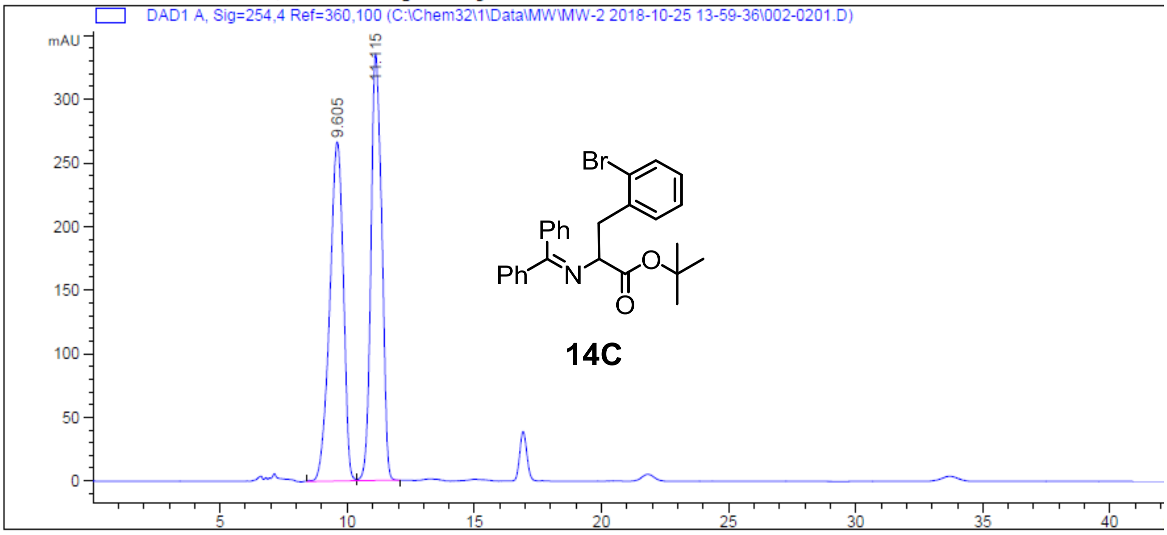

14C

```
=====================================================================
                         Area Percent Report
=====================================================================

Sorted By             :      Signal
Multiplier            :      1.0000
Dilution              :      1.0000
Do not use Multiplier & Dilution Factor with ISTDs


Signal 1: DAD1 A, Sig=254,4 Ref=360,100
```

Peak #	RetTime [min]	Type	Width [min]	Area [mAU*s]	Height [mAU]	Area %
1	9.605	BV	0.5879	9920.35547	266.72058	50.0998
2	11.115	VB	0.4896	9880.82813	335.50867	49.9002

Totals : 1.98012e4 602.22925

Sample Name: W STD K01

===

Acq. Operator : SYSTEM   Seq. Line : 1
Acq. Instrument : LC1200   Location : 1
Injection Date : 14.06.2019 16:21:31   Inj : 1
Inj Volume : 5.000 µl
Method : C:\Chem32\1\Data\MW\MW-2 2019-06-14 16-20-25\CHIR_ADH.M (Sequence Method)
Last changed : 14.06.2019 16:20:27 by SYSTEM
Sample Info : W STD K01/5-6 (RACEMAT) Chiralpak ADH 2-PrOH/heptane (5:95) 0.5 mL min-1, 25 °C

Additional Info : Peak(s) manually integrated

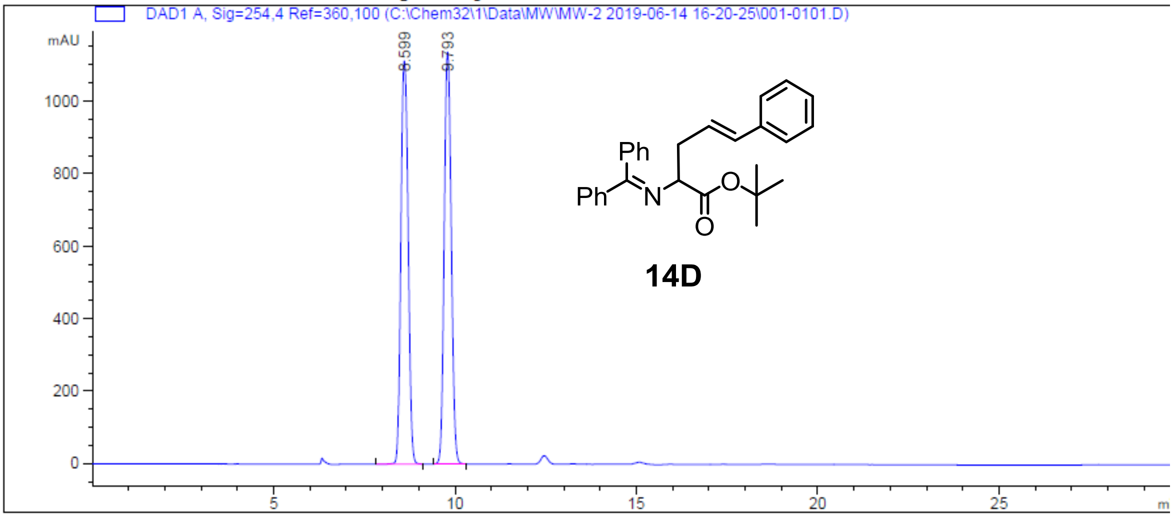

===

Area Percent Report

===

Sorted By : Signal
Multiplier : 1.0000
Dilution : 1.0000
Do not use Multiplier & Dilution Factor with ISTDs

Signal 1: DAD1 A, Sig=254,4 Ref=360,100

Peak #	RetTime [min]	Type	Width [min]	Area [mAU*s]	Height [mAU]	Area %
1	8.599	BB	0.2245	1.54999e4	1112.48364	50.0858
2	9.793	BB	0.2165	1.54468e4	1136.89917	49.9142
Totals :				3.09466e4	2249.38281	

===

*** End of Report ***

Sample Name: STD-B1

===

Acq. Operator : SYSTEM Seq. Line : 1
Acq. Instrument : LC1200 Location : 1
Injection Date : 25.10.2018 14:00:43 Inj : 1
Inj Volume : 5.000 µl
Acq. Method : C:\Chem32\1\Data\MW\MW-2 2018-10-25 13-59-36\CHIR_ADH.M
Last changed : 25.10.2018 14:01:05 by SYSTEM
(modified after loading)
Analysis Method : C:\Chem32\1\Data\MW\MW-2 2018-10-25 13-59-36\CHIR_ADH.M (Sequence Method)
Last changed : 25.10.2018 15:00:53 by SYSTEM
Sample Info : W STD-B1 Chiralpak ADH 2-PrOH/heptane (1:99) 0.5 mL min-1, 25 °C

Additional Info : Peak(s) manually integrated

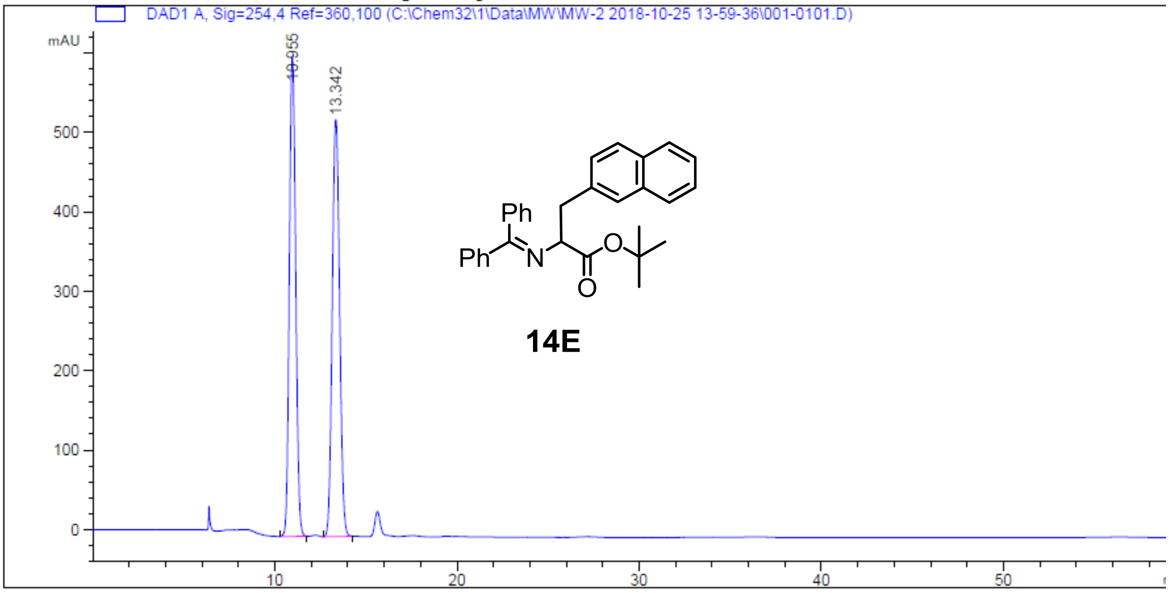

===

Area Percent Report

===

Sorted By : Signal
Multiplier : 1.0000
Dilution : 1.0000
Do not use Multiplier & Dilution Factor with ISTDs

Signal 1: DAD1 A, Sig=254,4 Ref=360,100

Peak #	RetTime [min]	Type	Width [min]	Area [mAU*s]	Height [mAU]	Area %
1	10.955	BB	0.3839	1.47045e4	604.30414	50.1185
2	13.342	VB	0.4392	1.46350e4	524.99420	49.8815

Totals : 2.93395e4 1129.29834

```
Sample Name: CHSC3/2

=====================================================================
Acq. Operator   : SYSTEM                           Seq. Line :   2
Acq. Instrument : LC1200                            Location :   2
Injection Date  : 15.02.2019 10:57:09                    Inj :   1
                                                  Inj Volume : 5.000 µl
Acq. Method     : C:\Chem32\1\Data\MW\BB-1_part 2019-02-15 10-55-53\CHIR_ADH.M
Last changed    : 15.02.2019 11:19:19 by SYSTEM
                  (modified after loading)
Analysis Method : C:\Chem32\1\Data\MW\BB-1_part 2019-02-15 10-55-53\CHIR_ADH.M (Sequence Method)
Last changed    : 15.02.2019 11:23:11 by SYSTEM
                  (modified after loading)
Sample Info     : CHSC 03 racem Prod, Chiralpak ADH, 5% 2-PrOH, 95% Heptan, 0.5 mlmin-1, 25 °C

Additional Info : Peak(s) manually integrated
```

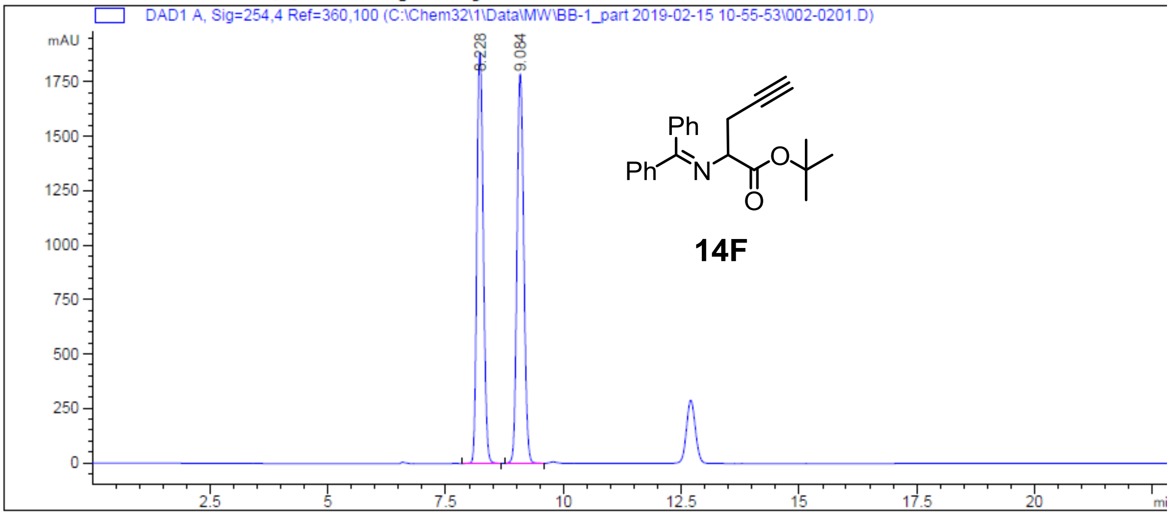


```
=====================================================================
                         Area Percent Report
=====================================================================

Sorted By             :      Signal
Multiplier            :      1.0000
Dilution              :      1.0000
Do not use Multiplier & Dilution Factor with ISTDs


Signal 1: DAD1 A, Sig=254,4 Ref=360,100

Peak RetTime Type  Width     Area      Height     Area
  #   [min]        [min]   [mAU*s]     [mAU]       %
----|-------|----|-------|----------|----------|--------|
   1   8.228 VB    0.1505 1.78747e4  1887.15088  49.8157
   2   9.084 BV    0.1574 1.80070e4  1790.12805  50.1843

Totals :                  3.58817e4  3677.27893
```

Sample Name: W STD H01

===

Acq. Operator : SYSTEM Seq. Line : 3
Acq. Instrument : LC1200 Location : 3
Injection Date : 29.05.2019 17:42:18 Inj : 1
Inj Volume : 5.000 µl
Acq. Method : C:\Chem32\1\Data\MW\MW-2 2019-05-29 16-38-47\CHIR_ADH.M
Last changed : 29.05.2019 17:43:15 by SYSTEM
(modified after loading)
Analysis Method : C:\Chem32\1\Data\MW\MW-2 2019-05-29 16-38-47\CHIR_ADH.M (Sequence Method)
Last changed : 29.05.2019 16:38:48 by SYSTEM
Sample Info : W STD H01/8-9 RACEMAT Chiralpak ADH 2-PrOH/heptane (5:95) 0.5 L min-1, 25 °C

Additional Info : Peak(s) manually integrated

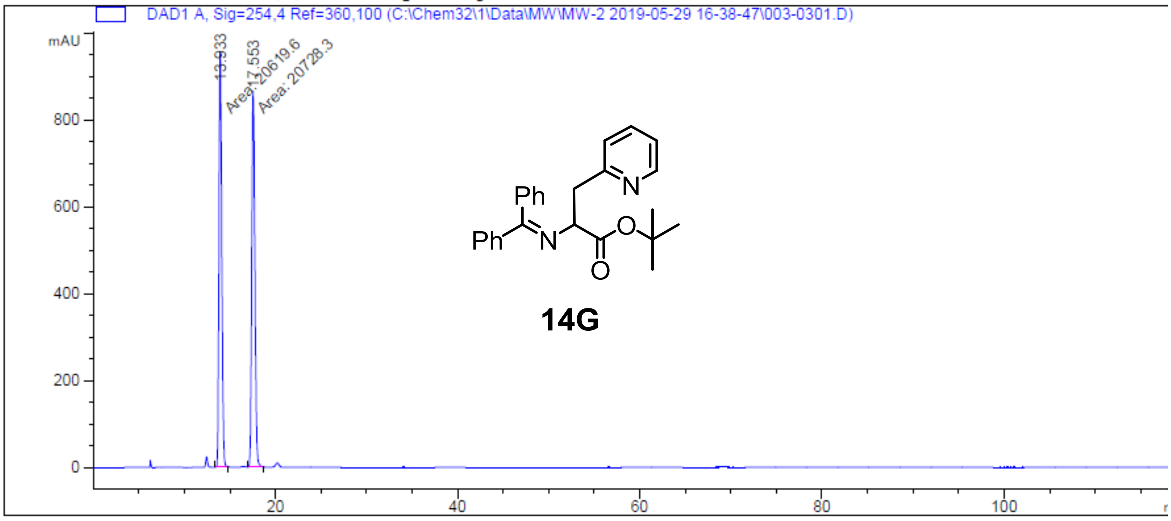

===

Area Percent Report

===

Sorted By : Signal
Multiplier : 1.0000
Dilution : 1.0000
Do not use Multiplier & Dilution Factor with ISTDs

Signal 1: DAD1 A, Sig=254,4 Ref=360,100

Peak #	RetTime [min]	Type	Width [min]	Area [mAU*s]	Height [mAU]	Area %
1	13.933	MF	0.3603	2.06196e4	953.83917	49.8685
2	17.553	MF	0.4002	2.07283e4	863.33826	50.1315

Totals : 4.13479e4 1817.17743

Sample Name: CHSC 1/3C/8-10

===

Acq. Operator : SYSTEM Seq. Line : 1
Acq. Instrument : LC1200 Location : 1
Injection Date : 15.02.2019 11:29:05 Inj : 1
Inj Volume : 5.000 µl
Acq. Method : C:\Chem32\1\Data\MW\BB-1_part 2019-02-15 11-28-01\CHIR_ADH.M
Last changed : 15.02.2019 11:54:50 by SYSTEM
(modified after loading)
Analysis Method : C:\Chem32\1\Data\MW\BB-1_part 2019-02-15 11-28-01\CHIR_ADH.M (Sequence Method)
Last changed : 15.02.2019 12:07:25 by SYSTEM
(modified after loading)
Sample Info : CHSC 01 racem Prod, Chiralpak ADH, 5% 2-PrOH, 95% Heptan, 0.5 mlmin-1, 25 °C

Additional Info : Peak(s) manually integrated

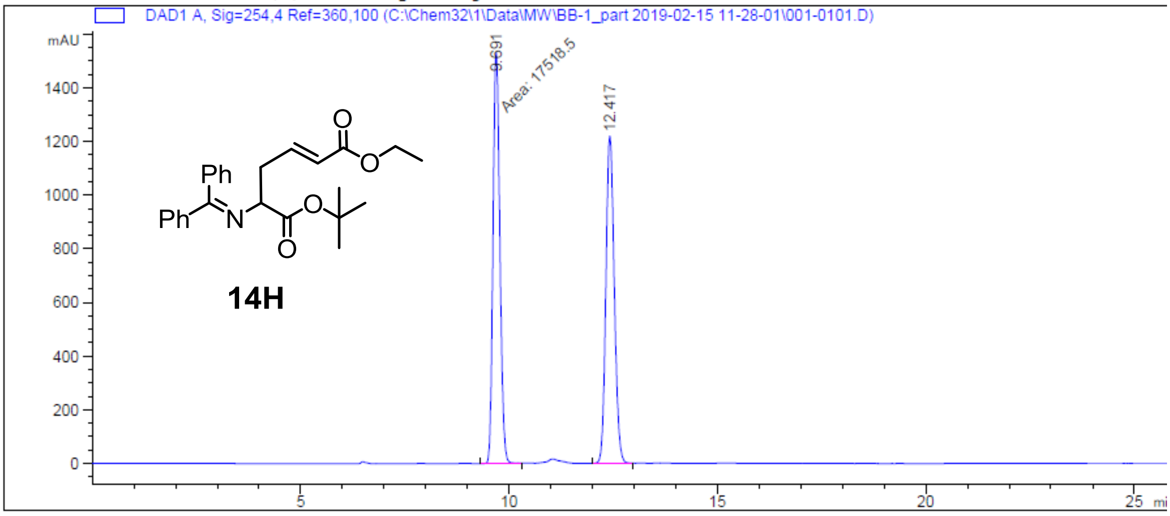

===
Area Percent Report
===

Sorted By : Signal
Multiplier : 1.0000
Dilution : 1.0000
Do not use Multiplier & Dilution Factor with ISTDs

Signal 1: DAD1 A, Sig=254,4 Ref=360,100

Peak #	RetTime [min]	Type	Width [min]	Area [mAU*s]	Height [mAU]	Area %
1	9.691	MF	0.1906	1.75185e4	1531.91235	50.6581
2	12.417	BB	0.2192	1.70633e4	1220.04663	49.3419

Totals : 3.45818e4 2751.95898

Sample Name: CHSC4/8-9

```
=====================================================================
Acq. Operator   : SYSTEM                          Seq. Line :    3
Acq. Instrument : LC1200                           Location :    3
Injection Date  : 14.02.2019 19:35:09                   Inj :    1
                                                 Inj Volume : 20.000 µl
Method          : C:\Chem32\1\Data\MW\BB-1_part 2019-02-14 19-33-25\CHIR_ADH.M (Sequence Method)
Last changed    : 14.02.2019 19:33:26 by SYSTEM
Sample Info     : CHSC 04 racem Prod, Chiralpak ADH, 5% 2-PrOH, 95% Heptan, 0.5 mlmin-1, 25 °C

Additional Info : Peak(s) manually integrated
```

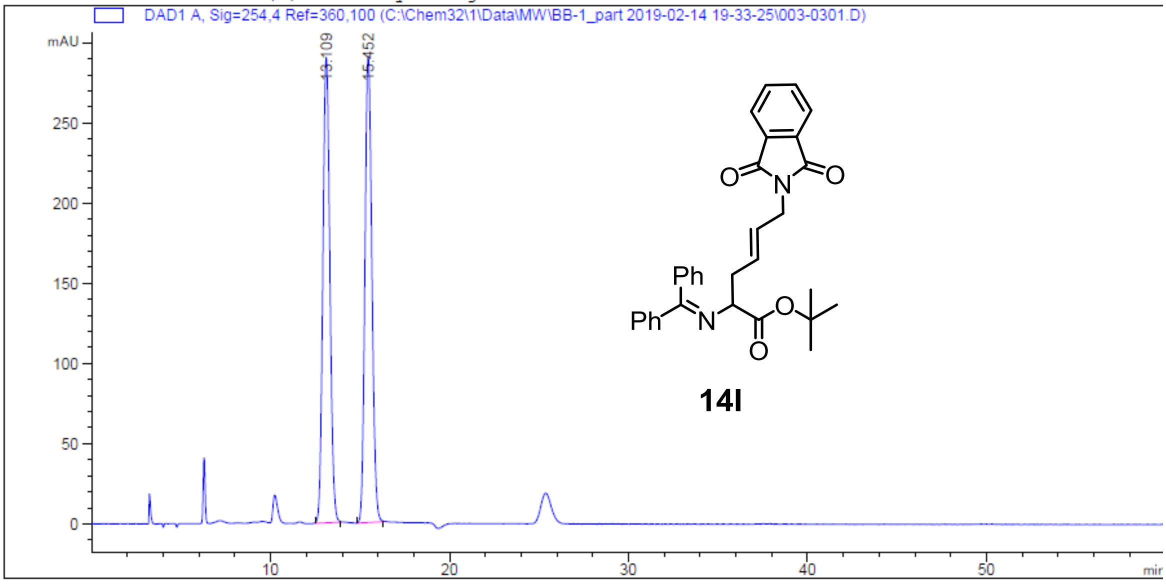

14l

```
=====================================================================
                          Area Percent Report
=====================================================================

Sorted By             :      Signal
Multiplier            :      1.0000
Dilution              :      1.0000
Do not use Multiplier & Dilution Factor with ISTDs


Signal 1: DAD1 A, Sig=254,4 Ref=360,100
```

Peak #	RetTime [min]	Type	Width [min]	Area [mAU*s]	Height [mAU]	Area %
1	13.109	BB	0.4288	7931.43066	290.25415	50.4199
2	15.452	BB	0.4187	7799.31787	290.97043	49.5801

Totals : 1.57307e4 581.22458

X-ray Structure Analyses

The X-ray intensity data were measured on Bruker D8 Venture and X8 APEX2 diffractometer equipped with multilayer monochromators, Mo K/α INCOATEC micro focus sealed tubes and Oxford and Cryoflex2 cooling systems. The structure was solved by *direct methods.* Non-hydrogen atoms were refined with *anisotropic displacement parameters*. Hydrogen atoms were inserted at calculated positions and refined with riding model. The following software was used: *Bruker SAINT software package* [2] using a narrow-frame algorithm for frame integration, *SADABS* [3] for absorption correction, *OLEX2* [4] for structure solution, refinement, molecular diagrams and graphical user-interface, *Shelxle* [5] for refinement and graphical user-interface *SHELXS-2015* [6] for structure solution, *SHELXL-2015* [7] for refinement*, Platon* [8] for symmetry check. Experimental data and CCDC-Codes (Available online: http://www.ccdc.cam.ac.uk/conts/retrieving.html) can be found in Table S2. Crystal data, data collection parameters, and structure refinement details are given in Tables S3 to S8. Crystal structures are visualized in Figures S1 to S5.

Table S2. Experimental parameter and CCDC-Codes.

**Sample**	**Machine**	**Source**	**Temp.**	**Detector Distance**	**Time/ Frame**	**#Frames**	**Frame width**	**CCDC**
	Bruker		[K]	[mm]	[s]		[°]	
(*R*)(*R*)-**1d**	X8	Mo	150	30	21	2236	0.700	1956062
(*R*)$_{bina}$(*S*)$_{biphe}$-**1e'**	X8	Mo	150	30	60	1049	0.700	1956063
[(*R*)-**7**]$_2$(*S*,*S*)-Dibenzoyltartrate	D8	Mo	100	30	30	1891	0.500	1956064

*(11bR,11b'R)-2,6-Di([1,1':4',1''-terphenyl]-4-yl)-3,3',5,5'-tetrahydro-4,4'-spirobi[dinaphtho[2,1-c:1',2'-e]azepin]-4-ium bromide (R)(R)-***1d**

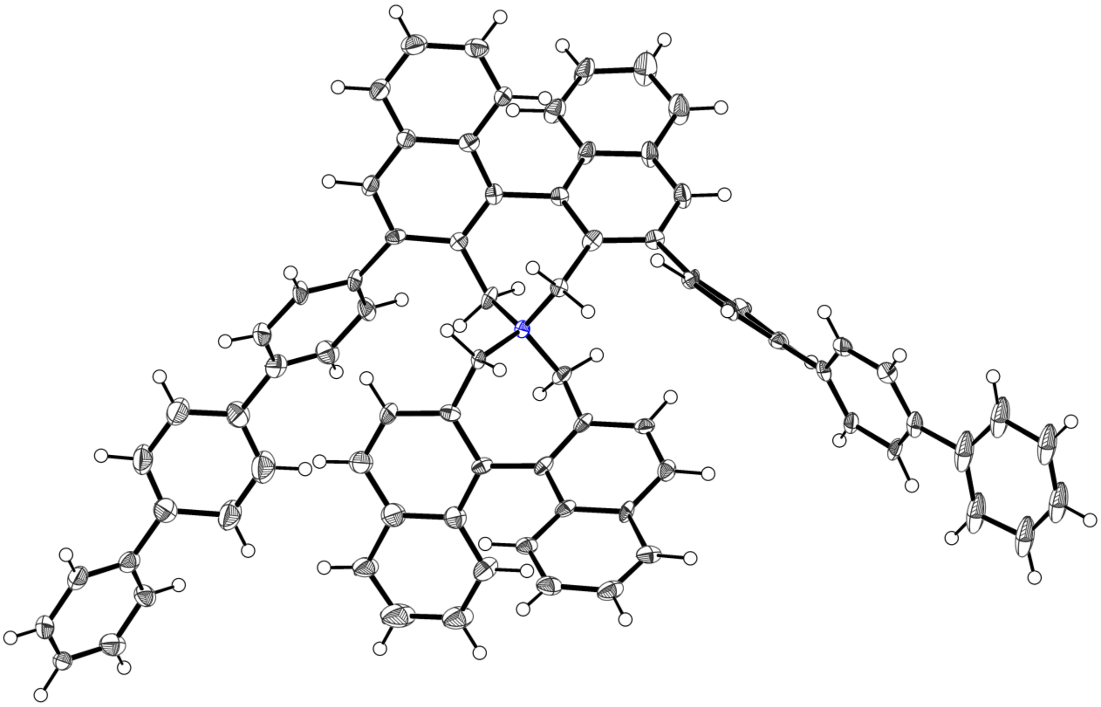

Figure S1. Crystal structure of (*R*)(*R*)-**1d**. C-C- Bond precision: 0.0121 Å. Solvent and counter ion omitted for clarity. Squeeze was used to cut the volume and the corresponding electron densities, because small electron densities in excluded volumes could not be matched. Details see cif-code.

Table S3. Sample and crystal data.

**Chemical formula**	$C_{82.5}H_{61}BrCl_5N$	**Crystal system**	orthorhombic	
**Formula weight [g/mol]**	1323.48	**Space group**	*P212121*	
**Temperature [K]**	150	**Z**	4	
**Measurement method**	\f and \w scans	**Volume [Å³]**	7229.8(11)	
**Radiation (Wavelength [Å])**	MoKα (λ = 0.71073)	**Unit cell dimensions [Å] and [°]**	9.1716(8)	90
**Crystal size / [mm³]**	0.3 × 0.14 × 0.11		26.018(2)	90
**Crystal habit**	clear colourless block		30.298(3)	90
**Density (calculated) / [g/cm³]**	1.216	**Absorption coefficient / [mm⁻¹]**	0.797	
**Abs. correction Tmin**	0.5297	**Abs. correction Tmax**	0.746	
**Abs. correction type**	multiscan	**F(000) [e⁻]**	2732	

Table S4. Data collection and structure refinement.

**Index ranges**	-11 ≤ h ≤ 11, -31 ≤ k ≤ 31, -36 ≤ l ≤ 36	**Theta range for data collection [°]**	2.064 to 50.7	
**Reflections number**	217019	**Data / restraints / parameters**	13222/0/838	
**Refinement method**	Least squares	**Final R indices**	all data	R1 = 0.0955, wR2 = 0.2433
**Function minimized**	$\Sigma\ w(F_o^2 - F_c^2)^2$		I>2σ(I)	R1 = 0.0812, wR2 = 0.2278
**Goodness-of-fit on F^2**	1.064	**Weighting scheme**	w=1/[σ2(Fo2)+(0.1579P)2 +14.4721P]	
**Largest diff. peak and hole [e Å^{-3}]**	1.91/-1.02		where $P=(F_o^2+2F_c^2)/3$	

*(R)-2',6'-Bis(4-tritylphenyl)-3',5,5',7-tetrahydrospiro[dibenzo[c,e]azepine-6,4'-dinaphtho[2,1-c:1',2'-e]azepin]-6-ium bromide (R)-**1e‘***

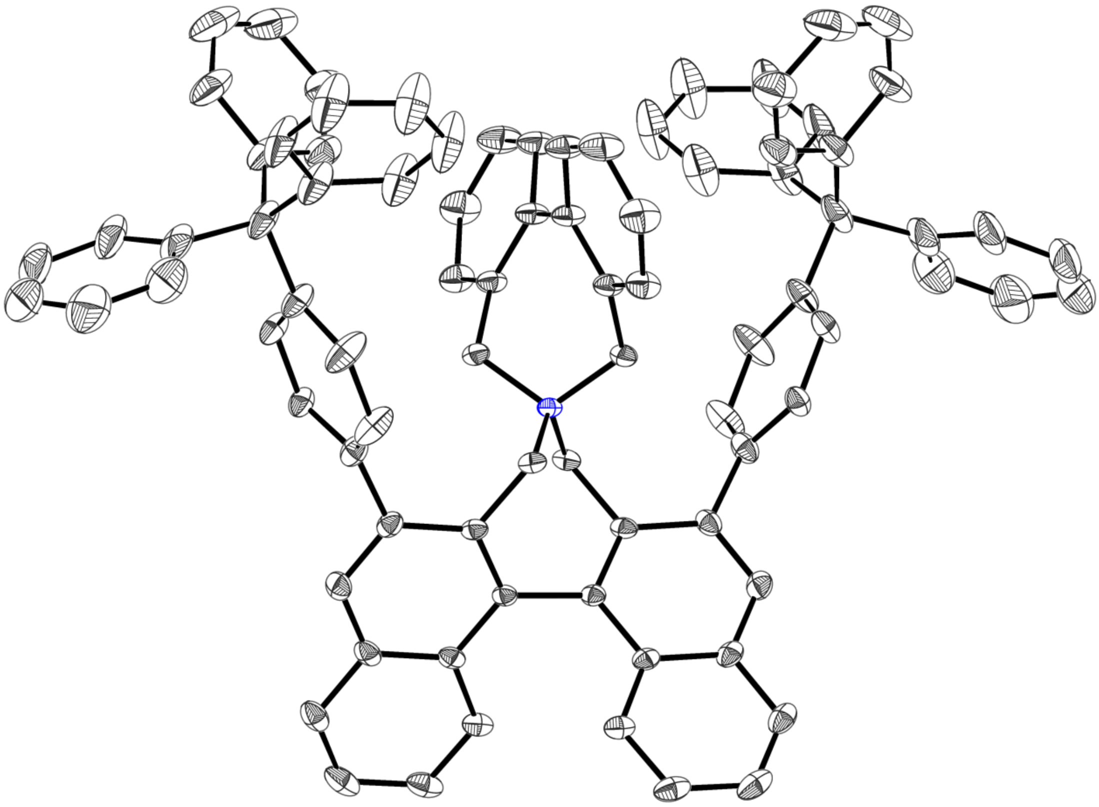

Figure S2. Crystal structure of (*R*)-**1e’**. C-C- Bond precision: 0.0074 Å. Solvent, counter ion and hydrogens omitted for clarity.

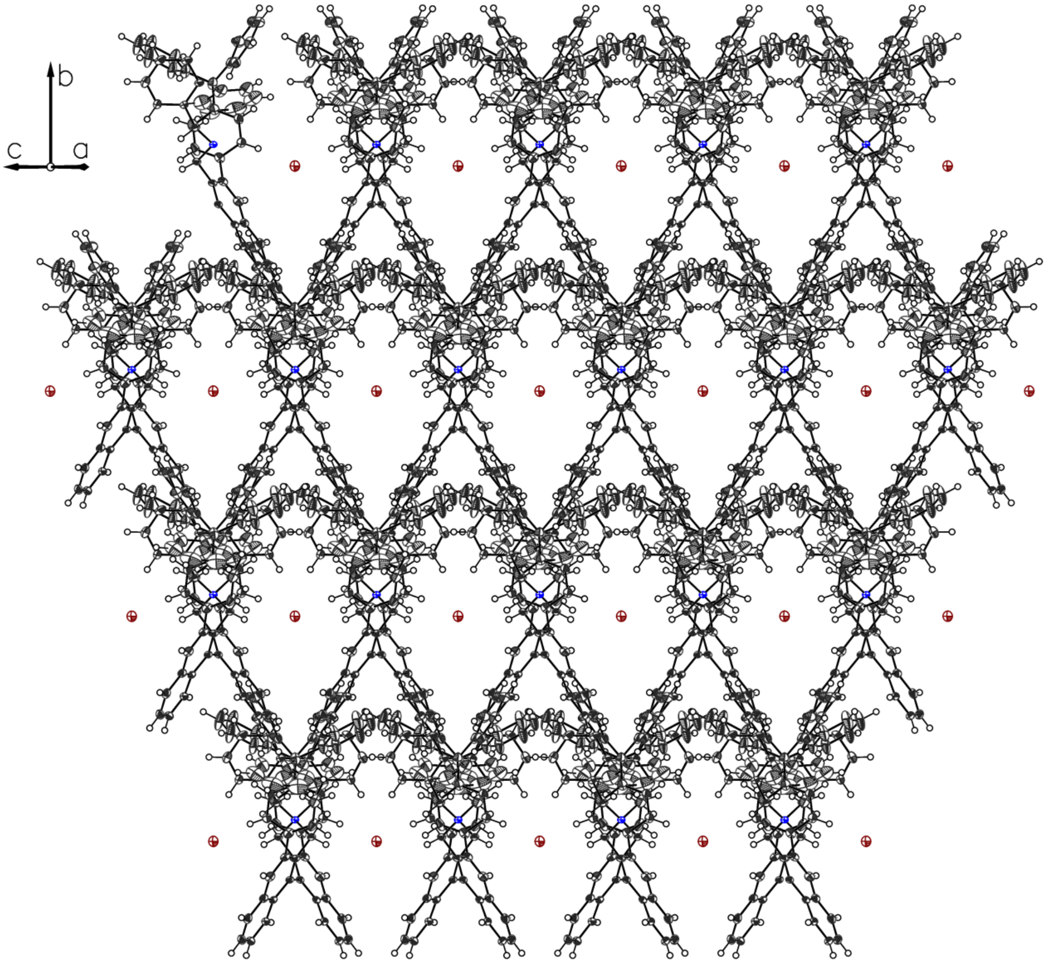

Figure S3. Packing view. Solvent omitted for clarity.

Table S5. Sample and crystal data.

**Chemical formula**	$C_{58}H_{48}Br_{0.5}N_{0.5}$	**Crystal system**	monoclinic	
**Formula weight [g/mol]**	791.92	**Space group**	*C2*	
**Temperature [K]**	150	**Z**	4	
**Measurement method**	\f and \w scans	**Volume [Å³]**	4478.3(5)	
**Radiation (Wavelength [Å])**	MoKα (λ = 0.71073)	**Unit cell dimensions [Å] and [°]**	21.7990(14)	90
**Crystal size / [mm³]**	0.25 × 0.23 × 0.1		23.2291(13)	94.251(2)
**Crystal habit**	clear colourless block		8.8684(5)	90
**Density (calculated) / [g/cm³]**	1.175	**Absorption coefficient / [mm⁻¹]**	0.51	
**Abs. correction Tmin**	0.5749	**Abs. correction Tmax**	0.746	
**Abs. correction type**	multiscan	**F(000) [e⁻]**	1668	

Table S6. Data collection and structure refinement.

**Index ranges**	$-30 \le h \le 29$, $-32 \le k \le 32$, $-12 \le l \le 12$	**Theta range for data collection [°]**	2.566 to 60.592	
**Reflections number**	43599	**Data / restraints / parameters**	13255/32/450	
**Refinement method**	Least squares	**Final R indices**	all data	R1 = 0.0876, wR2 = 0.2295
**Function minimized**	$\Sigma\ w(F_o^2 - F_c^2)^2$		I>2σ(I)	R1 = 0.0802, wR2 = 0.2193
**Goodness-of-fit on F^2**	1.048	**Weighting scheme**	w=1/[σ2(Fo2)+(0.1825P)2 +2.6917P]	
**Largest diff. peak and hole [e Å^{-3}]**	1.49/-0.65		where $P=(F_o^2+2F_c^2)/3$	

(*R*)-*2,6-Diiodo-4,5-dihydro-3H-dinaphtho[2,1-c:1',2'-e]azepin-4-ium ion* [(*R*)-**7**]$^+$

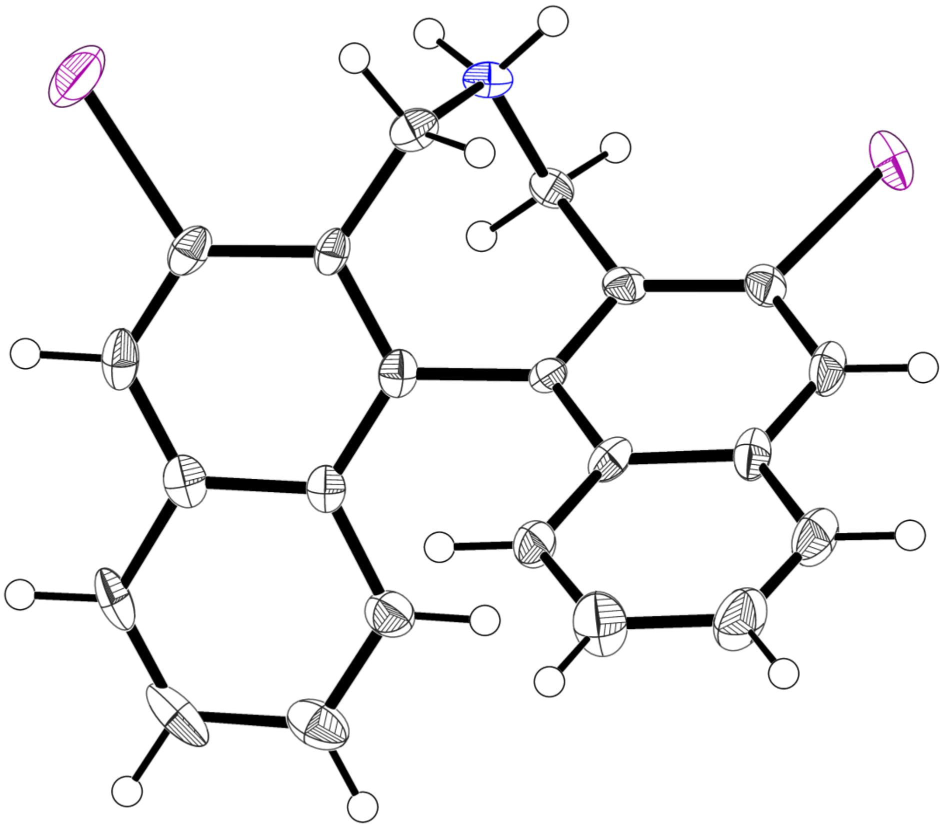

Figure S4. Crystal structure of [(*R*)-**7**]$_2$(*S,S*)-Dibenzoyltartrate. C-C- Bond precision: 0.0129 Å. Solvent and counter ion omitted for clarity.

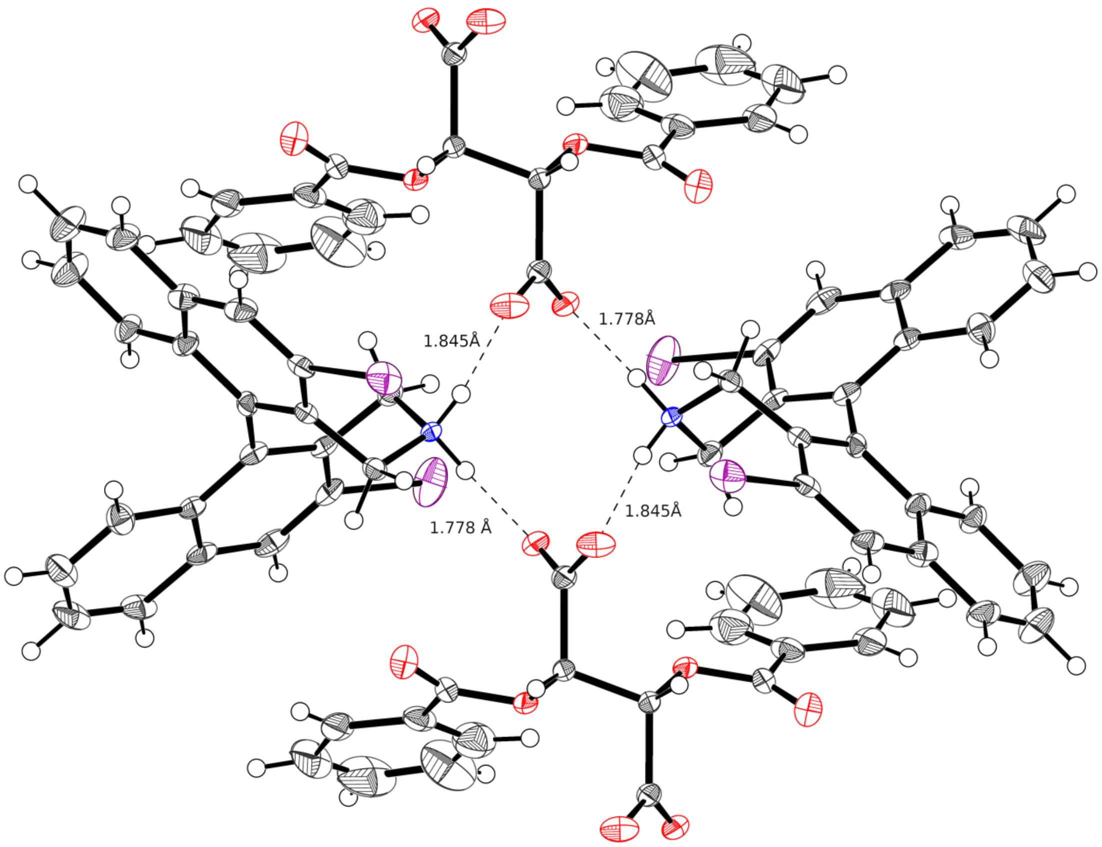

Figure S5. [(*R*)-**7**]$^+$ and counter ion form two independent hydrogen bonds because of symmetry for (two each because of symmetry reasons) construct a rhombus. Bond length and rhombus visualized.

Table S7. Sample and crystal data.

**Chemical formula**	$C_{32}H_{24}Cl_2I_2NO_4$	**Crystal system**	monoclinic	
**Formula weight [g/mol]**	811.22	**Space group**	*C2*	
**Temperature [K]**	100	**Z**	4	
**Measurement method**	\f and \w scans	**Volume [Å^3]**	3028.14(18)	
**Radiation (Wavelength [Å])**	MoKα (λ = 0.71073)	**Unit cell dimensions [Å] and [°]**	28.7403(11)	90
**Crystal size / [mm^3]**	0.07 × 0.07 × 0.05		12.0555(4)	101.095(2)
**Crystal habit**	clear colourless block		8.9062(3)	90
**Density (calculated) / [g/cm^3]**	1.779	**Absorption coefficient / [mm^{-1}]**	2.292	
**Abs. correction Tmin**	0.2263	**Abs. correction Tmax**	0.265	
**Abs. correction type**	multiscan	**F(000) [e$^-$]**	1580	

Table S8. Data collection and structure refinement.

**Index ranges**	$-34 \le h \le 34$, $-14 \le k \le 14$, $-10 \le l \le 10$	**Theta range for data collection [°]**	4.66 to 50.694	
**Reflections number**	47080	**Data / restraints / parameters**	5534/7/364	
**Refinement method**	Least squares	**Final R indices**	all data	R1 = 0.0367, wR2 = 0.0897
**Function minimized**	$\Sigma\ w(F_o^2 - F_c^2)^2$		$I>2\sigma(I)$	R1 = 0.0362, wR2 = 0.0894
**Goodness-of-fit on F^2**	1.129	**Weighting scheme**	w=1/[σ2(Fo2)+(0.0383P)2 +21.9719P]	
**Largest diff. peak and hole [e Å^{-3}]**	0.85/-1.24		where $P=(F_o^2+2F_c^2)/3$	

References

1. Ooi, T.; Kameda, M.; Maruoka, K. Design of *N*-Spiro C_2-Symmetric Chiral Quaternary Ammonium Bromides as Novel Chiral Phase-Transfer Catalysts: Synthesis and Application to Practical Asymmetric Synthesis of α Amino Acids. *J. Am. Chem. Soc.* **2003**, *125*, 5139–5151.
2. *Bruker SAINT* v8.38A/B & SAINT v7.56/7.68A; Copyright © 2005–2019 Bruker AXS: Madison, WI, USA.
3. Sheldrick, G.M. *SADABS;* University of Göttingen: Göttingen, Germany, 1996.
4. Dolomanov, O.V.; Bourhis, L.J.; Gildea, R.J.; Howard, J.A.K.; Puschmann, H. OLEX2: A complete structure solution, refinement and analysis program. *J. Appl. Crystallogr.* **2009**, *42,* 339–341.
5. Huebschle, C.B.; Sheldrick, G.M.; Dittrich, B. ShelXle: A Qt graphical user interface for SHELXL. *J. Appl. Crystallogr.* **2011**, *44*, 1281–1284.
6. Sheldrick, G.M. *SHELXS v 2016/4;* University of Göttingen: Göttingen, Germany, 2015.
7. Sheldrick, G.M. *SHELXL v 2016/4;* University of Göttingen: Göttingen, Germany, 2015.
8. Spek, A.L. Structure validation in chemical crystallography. *Acta Crystallogr.* **2009**, *D65*, 148–155.
